# Supplementary material for: Climate policy portfolios that accelerate emission reductions
Source: Nat Commun. 2026 Jan 23;17:1989. doi: 10.1038/s41467-026-68577-z (PMC12932820; doi:10.1038/s41467-026-68577-z)
Supplement: Supplementary file 1 — Supplementary Information [file 41467_2026_68577_MOESM1_ESM.pdf]

Climate policy portfolios that accelerate emission reductions

Theodoros Arvanitopoulos [§ 1,2]
Simon Bulian [§ 3,4]
Charlie Wilson [§ * 5,6]
Andrew J. Jordan [7]
Jale Tosun [3,4,8]
Nicholas Vasilakos [9]

[§] equally contributing authors
* corresponding author (charlie.wilson@eci.ox.ac.uk)

[1] Cardiff University, Cardiff Business School
[2] London School of Economics, Hellenic Observatory
[3] Heidelberg University, Institute of Political Science
[4] Heidelberg University, Heidelberg Center for the Environment
[5] University of Oxford, Environmental Change Institute
[6] International Institute for Applied Systems Analysis (IIASA)
[7] University of East Anglia (UEA), Tyndall Centre for Climate Change Research
[8] University of Oslo, Department of Political Science
[9] University of East Anglia (UEA), Norwich Business School

SUPPLEMENTARY INFORMATION 1 (SI1): DATA & MODELS

1 VARIABLES, DATA & DESCRIPTIVE STATISTICS

1.1 Fossil CO_2 emissions and emission intensities

1.2 Climate policy portfolio and (inter-)governmental organisation variables

1.3 Measuring climate policy density: IEA PMD vs CCLW datasets

1.4 Policy density

1.5 Policy dismantling

2 DUMMY VARIABLES & INTERACTION EFFECTS: TARGETS AND GOVERNMENTAL ORGANISATIONS

2.1 Interpreting coefficients

2.2 Roles of (inter)governmental organisations

3 CONTROL VARIABLES: SELECTION & JUSTIFICATION

3.1 Directed Acyclic Graphs (DAGs): confounders & backdoor paths

3.2 Bad controls & colliders

3.3 Secondary control variables & potential backdoor paths: business cycles & rule of law

3.4 Secondary control variables: temperature variation

3.5 Secondary control variables & fixed effects

4 POLICY DENSITY MODELS: INTERPRETING EFFECT SIZES

5 POLICY DENSITY MODELS: ROBUSTNESS CHECKS

5.1 Replicating Eskander and Fankhauser (2020)

5.2 Pre-2000 policies

5.3 Excluding China

5.4 Excluding Costa Rica

5.5 One year lagged policy density

5.6 Shannon Index diversity measures and 2020 Covid impact

5.7 Absolute emissions as dependent variable

5.8 Policy density by instrument type

5.9 Emission intensity by sector

5.10 Combinations of individual variables

5.11 Comparison with policy stringency models

6 ENDOGENEITY & CAUSALITY

6.1 Lagged models

6.2 Endogeneity tests

6.3 Granger non-causality tests

6.4 Fixed effects

6.5 Weak correlation between policy density & control variables

6.6 Supporting literature and policy vignettes

6.7 Our choice of econometric model

7 **SUPPLEMENTARY REFERENCES**

1 Variables, data & descriptive statistics

Table S1 summarises variables and data sources. Subsequent sections provide explanations and descriptive statistics.

Table S1. Summary of Data Sources.

Dependent variable	
CO_2 emission intensity ($kgCO_2$ per 2015 USD PPP)	IEA CO_2 Emissions From Fuel Combustion Database
Independent variables	
Cumulative policy density	IEA Policies and Measures Database (PMD)
Policy instrument type diversity (HHI* 3 categories)	IEA PMD + own coding
Policy instrument type diversity (HHI* 6 types)	
Policy sectoral coverage diversity (HHI*)	IEA PMD + own coding
Policy sectoral coverage diversity weighted (HHI*)	
Absolute 1990 emission-reduction target (dummy variable)	various inc. UNFCCC (Kyoto Protocol & Copenhagen Accord annexes, Paris Agreement INDCs & NDCs), Climate Action Tracker, Net Zero Tracker
Relative emission-reduction target (dummy variable)	
Energy and/or climate ministry (dummy variable)	Tosun [1]
Independent advisory body (dummy variable)	various inc. Evans and Duwe [2],Averchenkova, et al. [3],Weaver, et al. [4]
Membership of Clean Energy Ministerial (dummy variable)	Tosun and Mišić [5]
Control variables	
Rule of law (World Bank indicator)	World Bank, Worldwide Governance Indicators
Hodrick-Prescott GDP filter	Own estimation using World Bank, World Development Indicators
GDP per capita, PPP (constant 2021 international USD) and GDP per capita squared	World Bank, World Development Indicators
Imports of goods and services (% of GDP)	World Bank, World Development Indicators
Services, value added (% of GDP)	World Bank, World Development Indicators
Temperature variation (annual difference from long-term average 1990-2022)	World Bank, Climate Change Knowledge Portal

*HHI=Herfindahl-Hirschman Index

1.1 Fossil CO_2 emissions and emission intensities

Overall, fossil CO_2 emissions for the BRIICS countries have been trending upwards from 2000 to roughly 2015, after which growth rates have slowed [Figures S1 A-C]. In contrast, European Union (EU) countries (with Poland as an exception), along with the UK and USA, have seen downward trends in fossil CO_2 emissions, particularly over the last decade. Among the rest of the OECD countries, Colombia, Korea, and Turkey have all seen clear upward trends.

For the dependent variable in our analysis, we use emission intensity ($kgCO_2$ per unit of GDP) rather than absolute emissions in order to account for the large variation in country size and levels of economic activity in our sample [6]. After controlling for GDP, almost all countries in our sample experience declining trends in emission intensity over the last two decades [Figures S2 A-C].

1.2 Climate policy portfolio and (inter-)governmental organisation variables

Our climate policy portfolio variables measure policy density (cumulative numbers), sectoral coverage, instrument type, and long-term emission-reduction targets. In addition, we include a set of variables on governmental and intergovernmental organisations. Table S2 defines each and provides examples. Subsequent sections explain variable construction and provide descriptive statistics.

Table S2. Definition of terms: Climate policies and (inter-)governmental organisations.

Term	Definition	Example
Climate policy	Law (legislated act), executive order, programme, intervention or initiative relevant to mitigation.	*National Climate Change Law (Brazil).* Flagship 2009 legislation establishing emission reduction and sectoral targets including on efficiency, renewables, biofuels [7].
Climate policy sectoral coverage	GHG-emitting sector(s) targeted by a policy (energy, land use, buildings, transport, industry) or cross-sectoral, economy-wide.	*Clean Power Plan (US).* Federal 2015 policy limiting emissions in the energy supply sector [8].
Climate policy instrument	Type of instrument specified by a policy to reduce GHG emissions (i.e. the means to achieve mitigation).	*Carbon Tax (Estonia).* Market-based policy instrument introduced in 2000 setting a price on emissions [9].
Emission-reduction target	Absolute targets (compared to 1990 or other fixed reference year emission levels).<br>Relative targets (compared to business-as-usual, or reductions in emission intensity).<br>Net-zero targets.	Absolute: *NDC Target (USA).* The US committed to achieving 26-28% GHG emission reductions below 2005 levels by 2025 in its first NDC in 2016.<br>Relative: *NDC Target (Chile)*. Chile committed to reduce GHG intensity of GDP by 30% below 2007 levels by 2030 in its first NDC in 2017.
Climate policy-relevant governmental organisation	Advisory body: formal organisation involved in policy design, implementation, monitoring.	*Committee on Climate Change (UK).* Independent advisory body established in 2008 to monitor and hold government to account on progress towards legislated targets [10].
Climate policy-relevant intergovernmental organisation	Climate forum: formal international organisation enabling climate relevant knowledge exchange and policy diffusion among governmental bodies.	*Clean Energy Ministerial.* Global forum for advancing and promoting clean energy technology diffusion among its member countries and companies [11].

Data on climate policy variables (density, sectoral coverage and instruments) is derived from the IEA Policies and Measures Database (PMD), see next section. Since a comprehensive overview on types of national emission reduction targets is not available, we had to collect the data from various public sources, including UNFCCC (Kyoto Protocol & Copenhagen Accord annexes, Paris Agreement INDCs & NDCs), Climate Action Tracker, and Net Zero Tracker.

Data on the existence of an energy ministry and/or a climate ministry is taken from Tosun [1] and updated to 2022 based on various public sources. Information on independent advisory bodies was collected by the authors from various sources (see Table S1)[12]. Membership in the Clean Energy Ministerial (CEM) is derived from data provided by Tosun and Mišić [5].

1.3 Measuring climate policy density: IEA PMD vs CCLW datasets

There are two main datasets relevant to climate policies: the International Energy Agency (IEA) Policies and Measures Database (PMD), and the Climate Change Laws of the World (Schaub et al., 2022).

The IEA PMD compiles past and existing energy-related climate policies and measures globally using data supplied by governments and IEA partner organisations [13]. The data we use covers the period from 2000 to 2022 and provides detailed information on the year a policy was adopted, the policy instrument types it employs (including carbon taxes, renewable energy incentives, energy efficiency standards, and information-based initiatives), and the sectors it targets for GHG emission reduction. There is a strong overlap between energy policy and climate mitigation policy classifications; typically climate mitigation policy has emission reductions as at least one of its objectives [14].

Eskander & Fankhauser (2020) use the Climate Change Laws of the World Database (CCLW) to construct their long-term and short-term policy density variables. They include a total of 1,092 climate laws and policies. Managed by the Grantham Research Institute, the CCLW dataset compiles parliamentary acts, executive orders, and policies of equivalent importance relevant to GHG emission reductions (https://climate-laws.org/) [15]. These range from broad framework laws establishing long-term objectives and an overarching legal basis for mitigation to specific sectoral policies on energy, transport, and land use [16].

Selection of policies for the CCLW database is based on legal documents, so it is particularly useful for assessing climate policy portfolios at a global level (Schaub et al., 2022). However, the CCLW database only captures active climate laws and not those that have been repealed (Eskander & Fankhauser, 2020) and thus has shortcomings for historical analysis. In contrast, the IEA PMD has more extensive coverage of climate policies and provides in-depth information on policy instrument types. As a result, the IEA PMD reports a larger number of climate policies and is more suitable for assessing policy portfolios for individual countries as it includes detailed information on policy instrument types and sectoral coverage.

1.4 Policy density

We derive our policy density measure from the IEA PMD (for details see the section on Data in Methods). Figures S3 A-C show line plots of total policy density by country over time, showing clear upward trends for all countries in our dataset. Larger numbers of new climate policies can be observed in the years following 2006. The BRIICS economies along with certain OECD countries (Chile, Israel, Italy, and Lithuania) have been slow to start, but experienced a rapid increase in the overall number of climate policies from 2007 and onwards. The rest of the OECD countries have displayed more gradual adoption of climate policies over time. Focusing specifically on BRIICS, we observe that they experience an even steeper upward trend for total policy density from 2010 onwards. China is an important outlier given it has legislated annually a very large number of climate policies but mainly since 2011: e.g., 22 policies in 2011, 26 in 2014, and 31 in 2016. By 2018, countries with the largest numbers of climate policies are China, USA, Australia, UK, Canada, and Spain.

China is the only country from the block of BRIICS economies that closely follows the larger OECD countries in terms of total policy density.

1.5 Policy dismantling

The IEA PMD does not consistently report when policies end, so we were unable to capture discontinued or ‘dismantled’ policies in our policy density measure. However, this does not bias our analysis for two reasons. First, the dismantling of policies is relatively infrequent. Schaub, et al. [17] show that only a small fraction of climate policies terminate over time. Second, the dismantling of climate policies correlates with low adoption of new climate policies. So even some policies are dismantled by a government with low climate ambition over a certain period, the effect of dismantling is included in the slow or negligible increase in policy density during this same period.

As an example, in Australia a change in government in 2013 ended the carbon pricing scheme. This dismantling of policies also coincided with few newly adopted climate policies. Over the period 2000-2022, there is a clear inverse correlation between policy adoption and dismantling (see Figure S14 in the Supplementary File of Schaub, et al. [18]). So even if we could incorporate the dismantling of policies in our density measure, we would not expect it to affect our model results.

Figure S1A. Fossil CO_2 emissions (natural log) per country from 2000 to 2022 for OECD countries in the EU. Vertical axis rescaled by country to visualize trends in fossil CO_2 emissions.

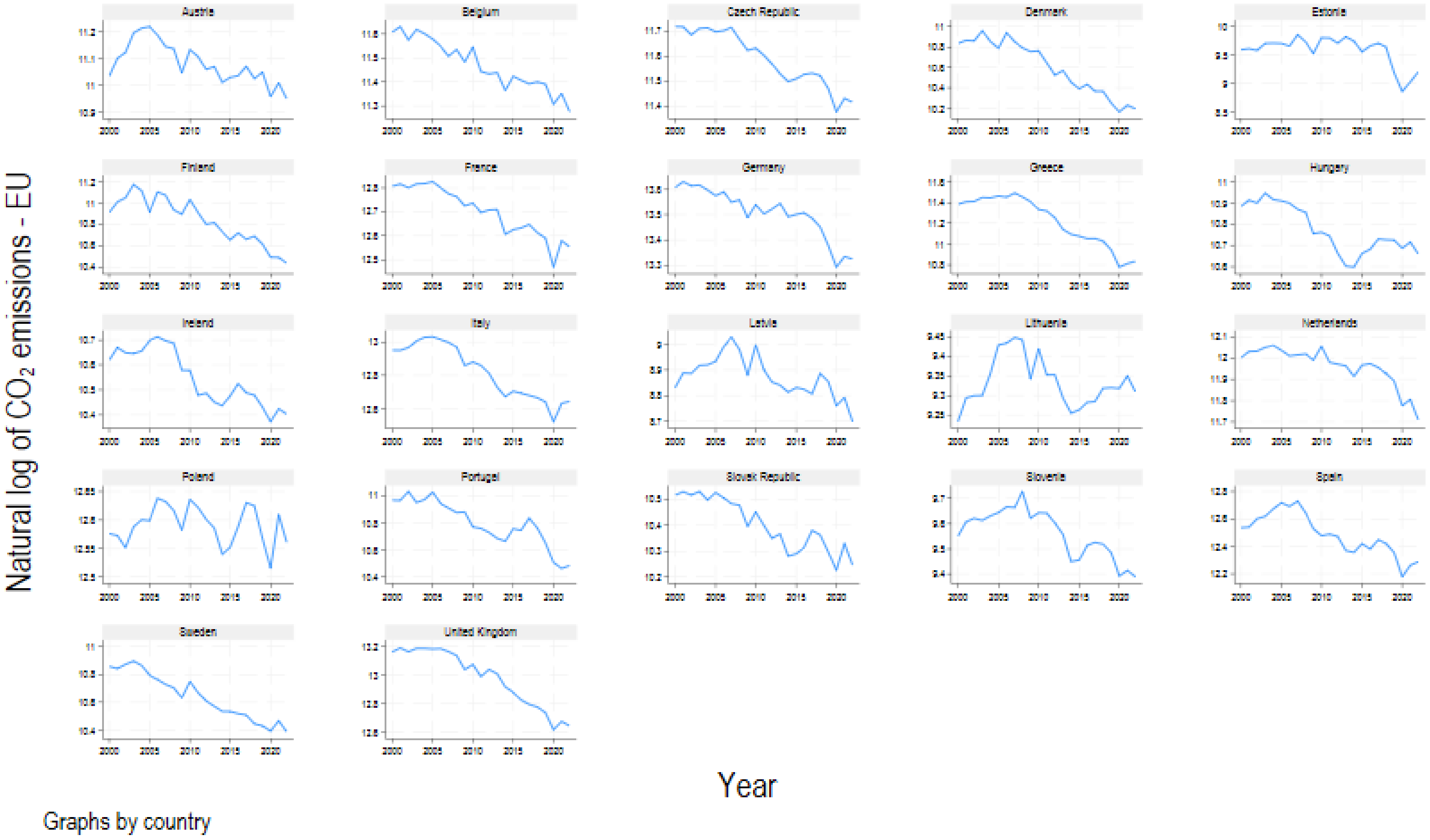

Figure S1B. Fossil CO_2 emissions (natural log) per country from 2000 to 2022 for non-EU OECD countries. Vertical axis rescaled by country to visualize trends in fossil CO_2 emissions.

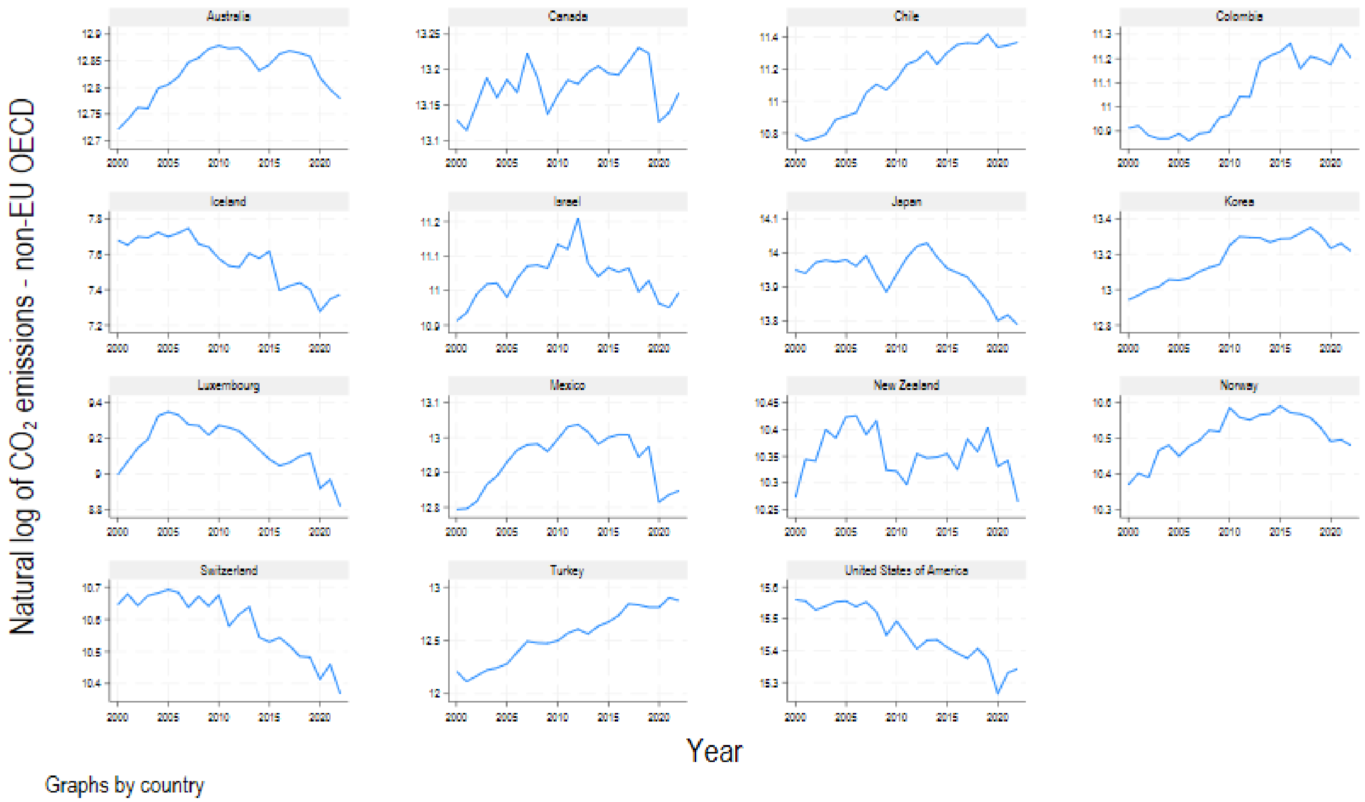

Figure S1C. Fossil CO_2 emissions (natural log) per country from 2000 to 2022 for BRIICS. Vertical axis rescaled by country to visualize trends in fossil CO_2 emissions.

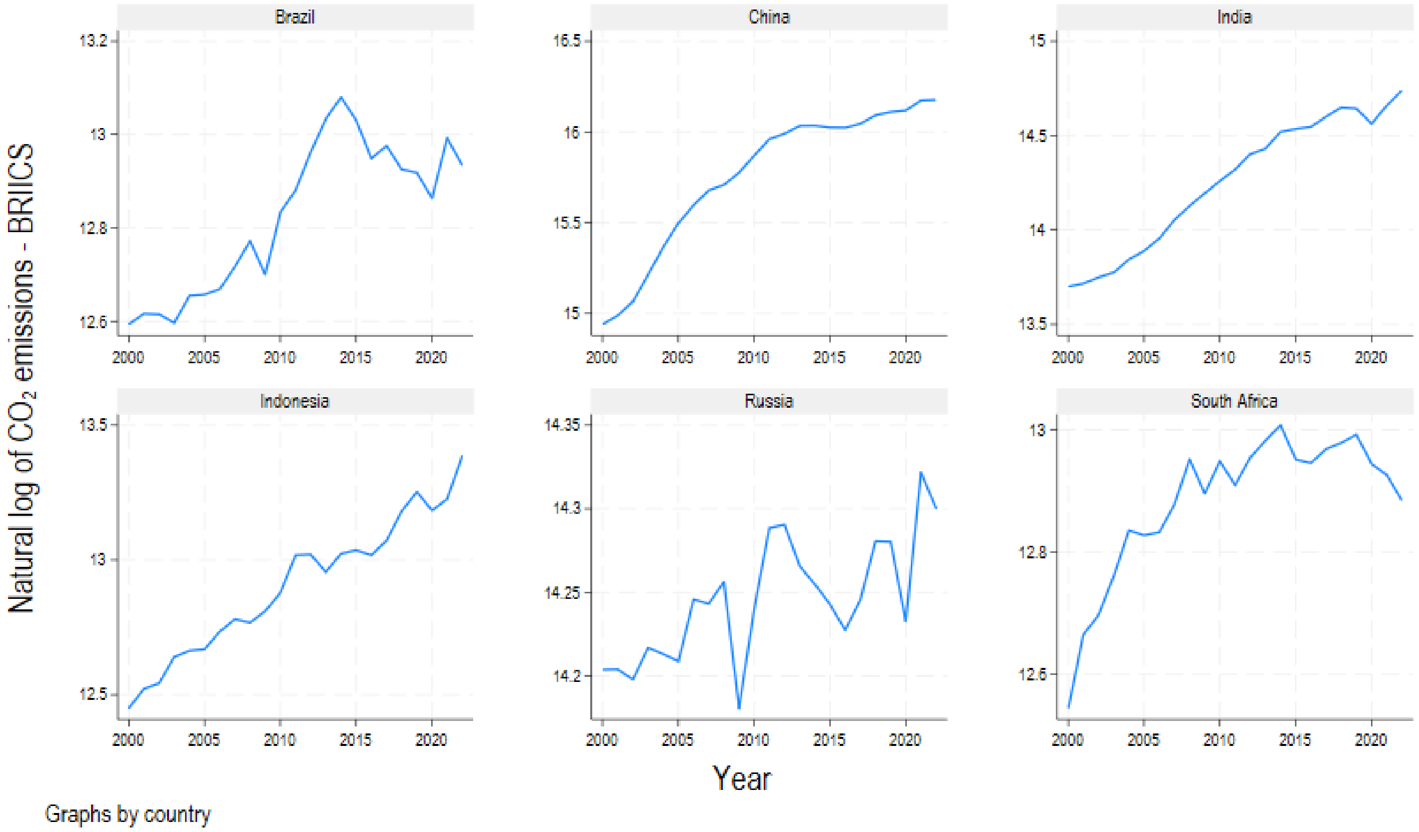

Figure S2A. Fossil CO_2 emissions intensity (CO_2/GDP) per country from 2000 to 2022 for OECD countries in the EU. Vertical axis rescaled by country group min max range to visualize country differences in the level and speed of reduction of emissions intensity.

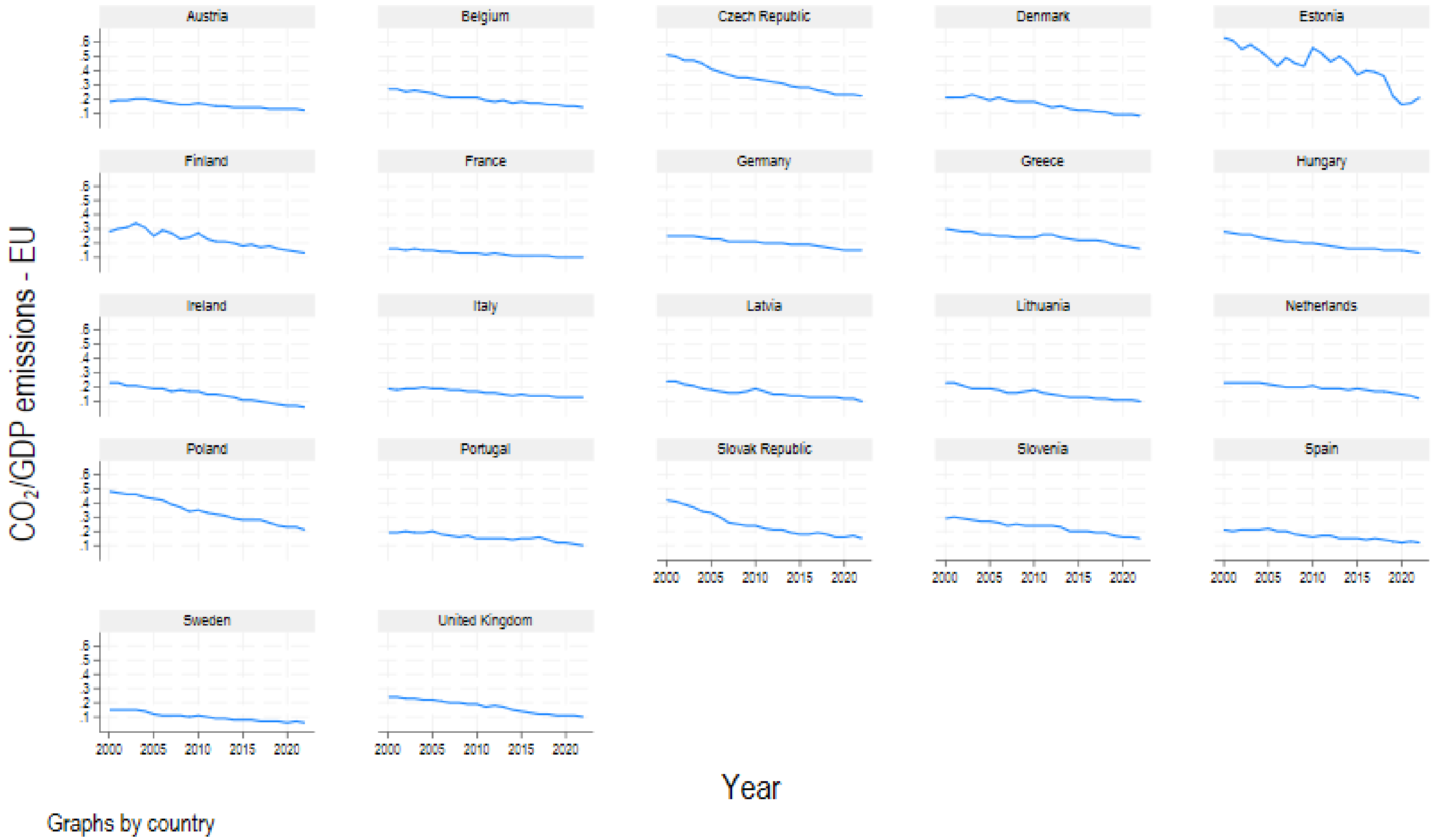

Figure S2B. Fossil CO_2 emissions intensity (CO_2/GDP) per country from 2000 to 2022 for non-EU OECD countries. Vertical axis rescaled by country group min max range to visualize country differences in the level and speed of reduction of emissions intensity.

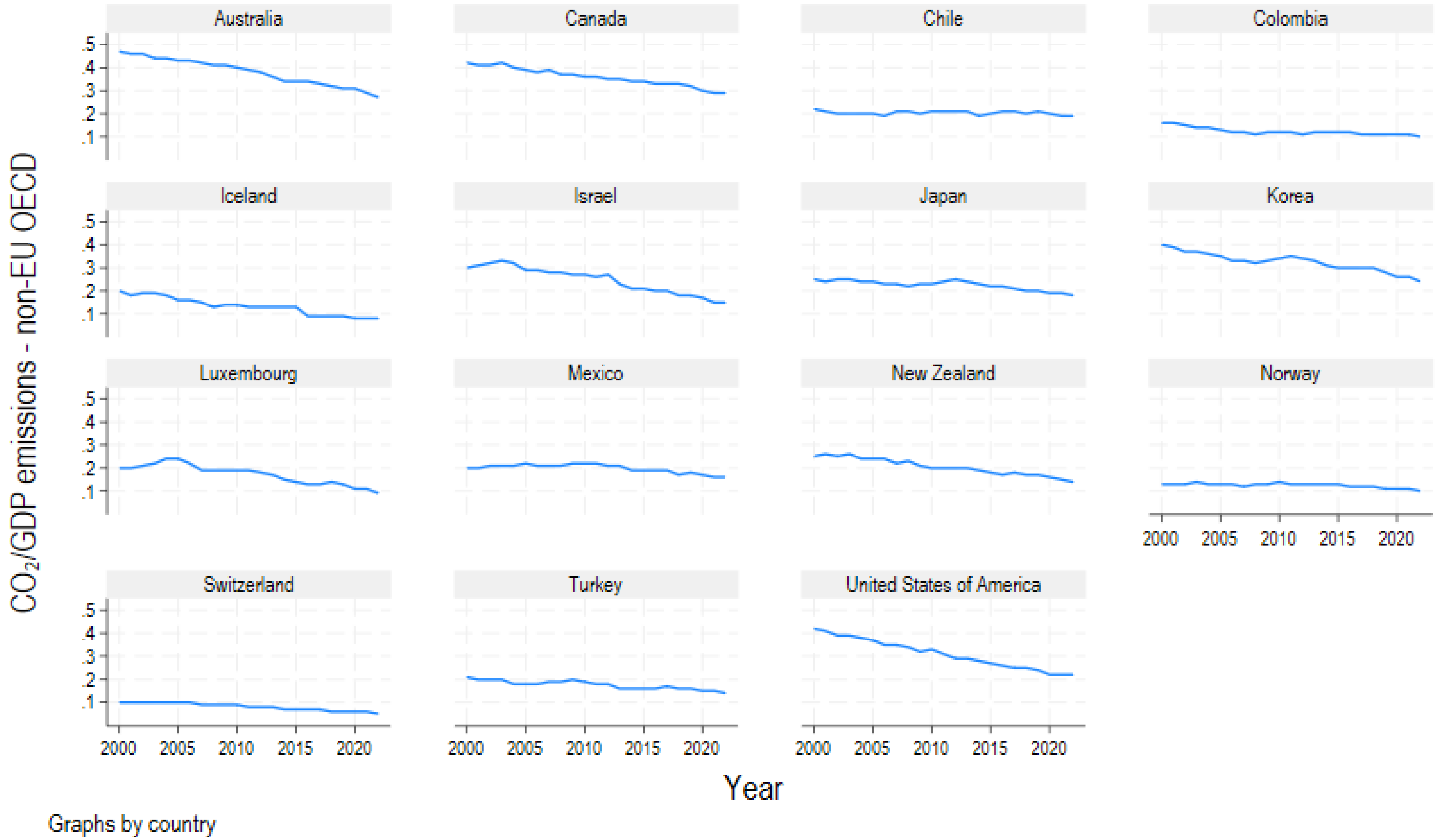

Figure S2C. Fossil CO_2 emissions intensity (CO_2/GDP) per country from 2000 to 2022 for BRIICS. Vertical axis rescaled by country group min max range to visualize country differences in the level and speed of reduction of emissions intensity.

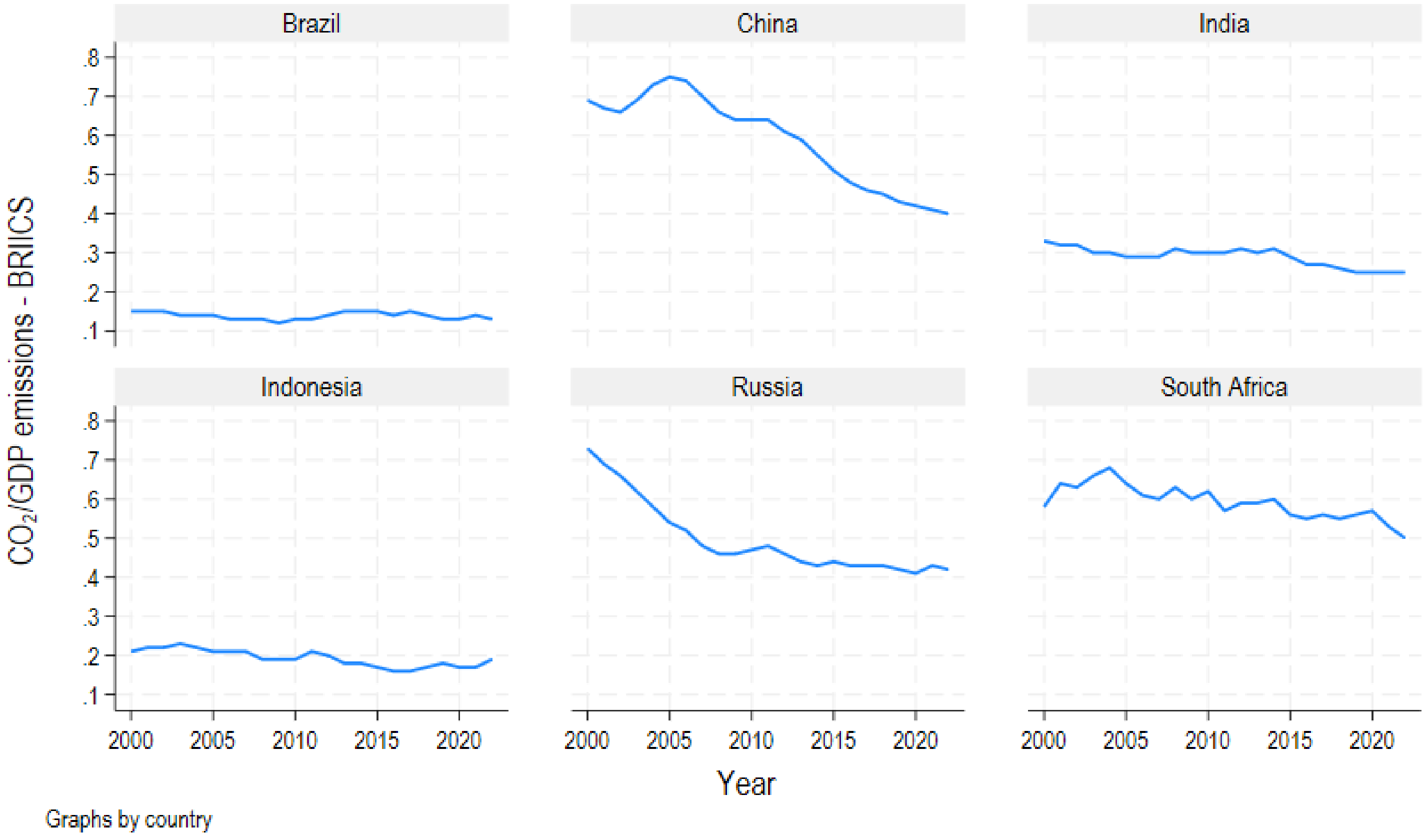

Figure S3A. Cumulative number of climate policies per country adopted from 2000 to 2022 for OECD countries in the EU. Vertical axis rescaled by country group min max range to visualize country differences in the level and speed of expansion of climate policies.

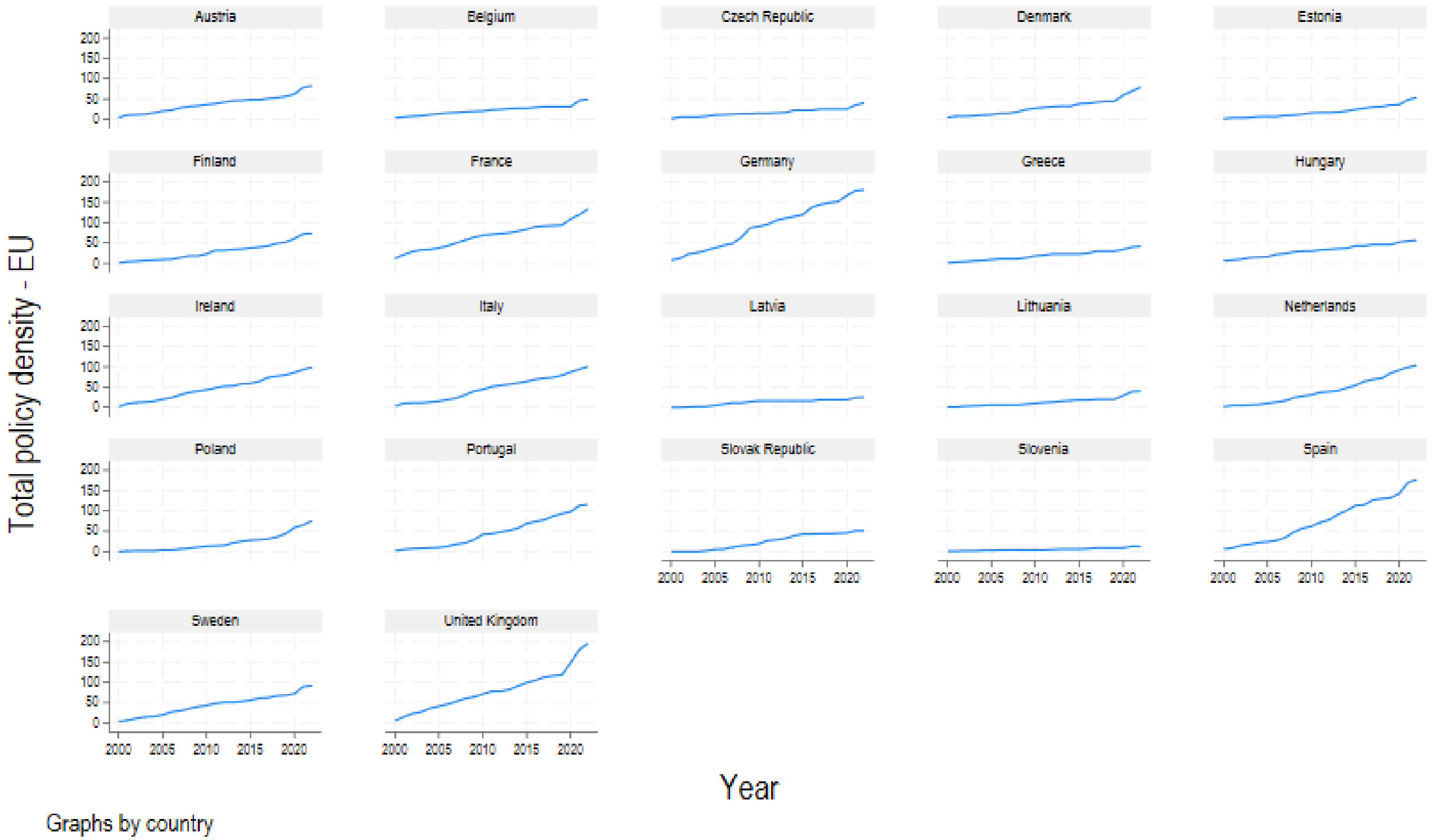

Figure S3B. Cumulative number of climate policies per country adopted from 2000 to 2022 for non-EU OECD. Vertical axis rescaled by country group min max range to visualize country differences in the level and speed of expansion of climate policies.

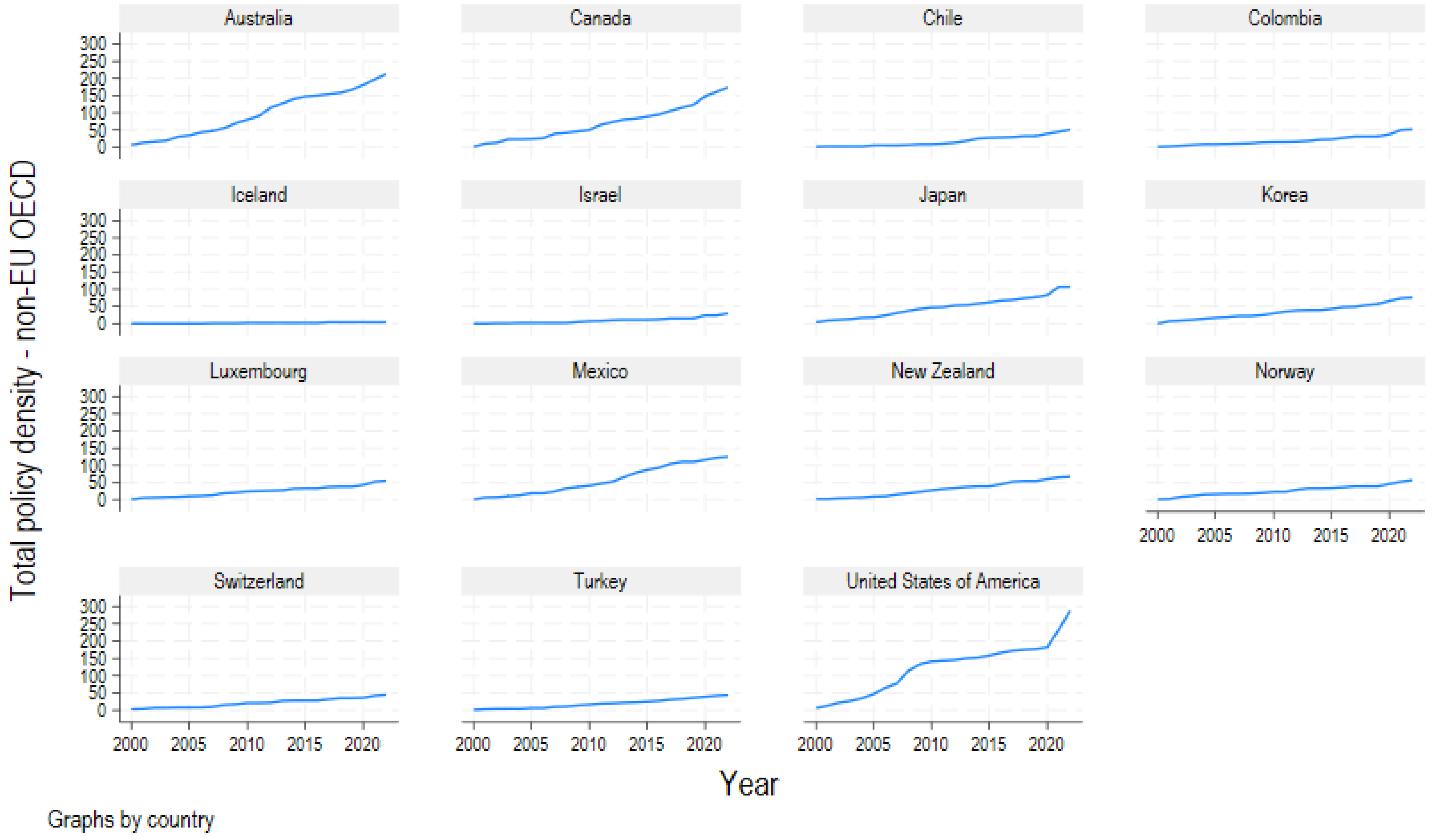

Figure S3C. Cumulative number of climate policies per country adopted from 2000 to 2022 for BRIICS. Vertical axis rescaled by country group min max range to visualize country differences in the level and speed of expansion of climate policies.

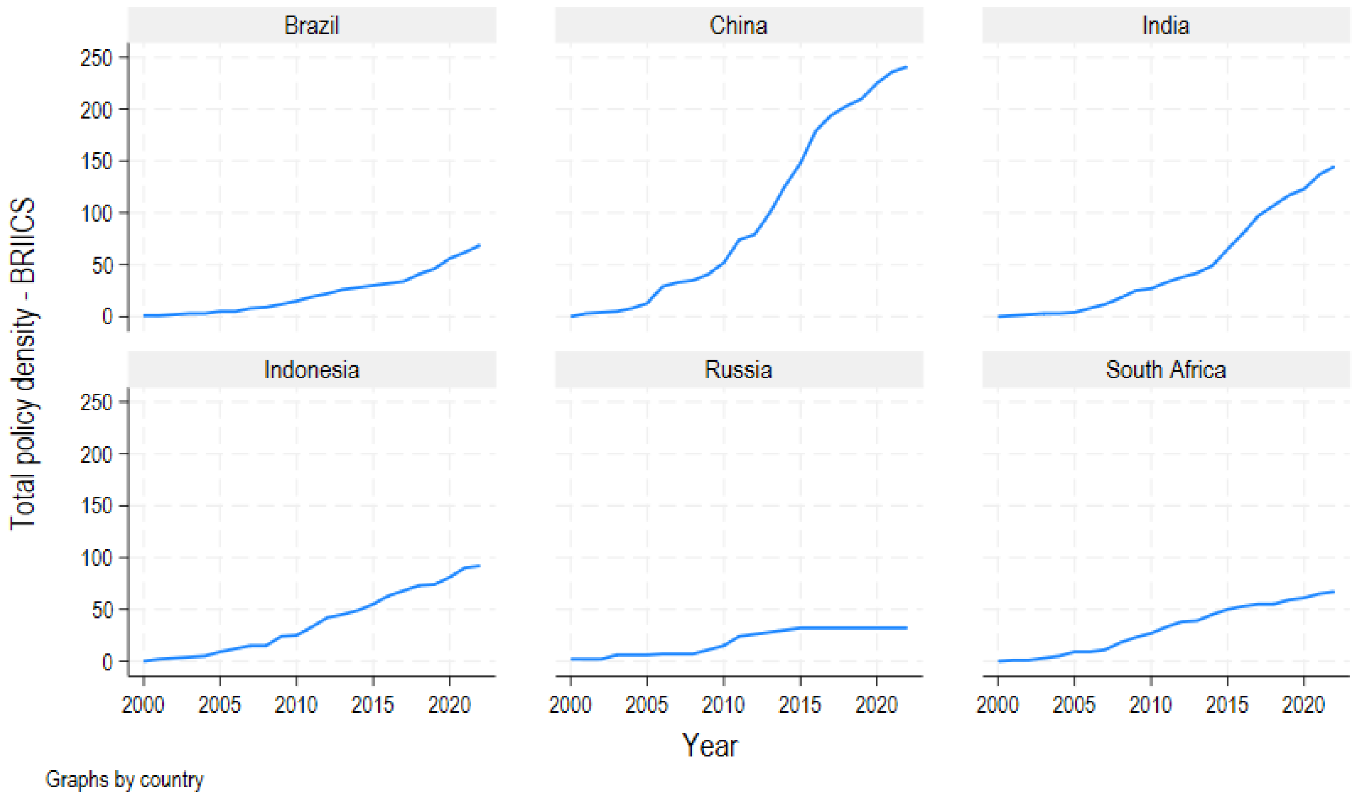

2 Dummy variables & interaction effects: targets and governmental organisations

2.1 Interpreting coefficients

As noted in Methods, our policy density models with interaction effect models are specified as:

$$\ln\left(\frac{CO2}{GDP}\right)_{it} = \alpha + \beta_1 Pdens_{it} + \beta_2 X_{it} + \delta_0 dummy_{it} + \delta_1 Pdens * dummy_{it} + \theta_\iota + \eta_\tau + \varepsilon_{it} \quad (1)$$

where $\ln\left(\frac{CO2}{GDP}\right)_{it}$ stands for the natural log of emissions intensity per country *i* and year *t*; α is the constant parameter; $Pdens_{it}$ is the total policy density measuring the cumulative number of climate policies per country *i* for year *t*; X_{it} stands for all controls; $dummy_{it}$ accounts for the presence/absence of climate governance institutions; $Pdens * dummy_{it}$ is the interaction term between policy density and the dummy; θ_ι are the country fixed effects; η_τ are year fixed effects; and residuals ε_{it}.

The coefficients δ_0 and δ_1 represent the intercept and slope parameters, respectively, for the effect of total policy density on emissions intensity when dummy=1, while α and β_1 represent the intercept and slope parameters for the effect of policy density on emissions intensity when dummy=0. More specifically, the coefficient δ_0 captures the emission intensity differential, when total policy density is equal to 0, between *i* and *t* when dummy=0 and *i* and *t* when dummy=1. The coefficient β_1 measures the effect of total policy density on historical emissions intensity when dummy=0, while the coefficient δ_1 (interaction term) measures the difference in the effect of total policy density on emission intensity between *i* and *t* when dummy=1 and *i* and *t* when dummy=0. Therefore, the overall estimated effect of total policy density on historical emissions intensity when dummy=1 can be acquired by summing coefficients β_1 and δ_1 (on the condition that both coefficients reject the null hypothesis).

If the coefficient δ_1 of the interaction term does not reject the null hypothesis but the joint hypothesis (*F* test with H_0: $\delta_0 = 0$ and $\delta_1 = 0$) does reject the null, then the two slope terms (β_1 and δ_1) are effectively parallel to each other, and constantly differ by δ_0. This essentially indicates no difference in the effect of policy density between *i* and *t* when dummy=1 and *i* and *t* when dummy=0, except in the case that total policy density=0 (in which case they differ by δ_0). However, this case has no practical meaning in our analysis as we are interested in the difference in the effect of total policy density when climate policies exist.

If both δ_0 and δ_1 parameters are zero (this holds if we cannot reject the null hypothesis of the joint *F* test), then the coefficient β_1 captures the effect of total policy density on emissions intensity for all cases. This means that the effect of total policy density on emissions intensity reduction is identical between *i* and *t* when dummy=1 and *i* and *t* when dummy=0.

Finally, focusing on the interpretation of the regression model coefficients, in our base model (model 1, Table 2 in main text) the coefficient for policy density is -0.000554. This is an order of magnitude smaller than in Eskander and Fankhauser [6] for which the coefficient for current policy stock (t-1) is -0.0078 and for older policy stock (t-3) is -0.0179. Accounting for the fact that there are various differences in the samples used in the two studies (countries, time-period, and policy data), the difference in magnitude can be explained by the fact that we include ‘policies’ (including, for example, informational and voluntary measures) which can be weaker than ‘laws’.

Indeed, once we control for the interaction between policies and absolute emission-reduction targets, the corresponding interaction coefficients are of the same order of magnitude to those for policy density in Eskander and Fankhauser [6]. See Table S3 for full results. This is specifically true for the case of EU OECD countries included in Appendix Table S2 in Eskander and Fankhauser [6], which is more closely comparable to our analysis. This suggests that under the presence of absolute targets the effectiveness of policies increases to a comparable magnitude to that observed for 'laws' in Eskander and Fankhauser [6].

Similar results are found for membership of the IEA, CEM, and EU-EFTA (see Table S4). The coefficient for the interaction term for countries that are members of IEA is statistically significant, negative, and smaller in magnitude (in absolute terms) than the coefficient for the interaction term for members of the EU-EFTA, which is also negative and statistically significant.

The negative and statistically significant dummy coefficient for energy and/or climate ministries and the negative and weakly significant dummy coefficient for membership of the IEA indicate that these countries start off with lower emission intensities than the rest. In comparison, the dummy coefficient for membership of the CEM is statistically significant and positive, indicating that these countries start off with higher emission intensities than the rest, accounting for the fact that non-IEA are members of the CEM.

Table S3. Long-term emission reduction targets

VARIABLES	base<br>(1)<br>Log(CO2/GDP)	absolute target<br>(2)<br>Log(CO2/GDP)	absolute target check<br>(3)<br>Log(CO2/GDP)	relative target<br>(4)<br>Log(CO2/GDP)
Policy density	-0.000554***	-9.47e-05	-0.000634***	-0.000320
	(0.000169)	(0.000178)	(0.000171)	(0.000197)
Absolute with 1990 baseline emission targets (dummy)		0.0157	-0.0660***	
		(0.0212)	(0.0169)	
Policy density * Absolute with 1990 baseline emission targets		-0.00154***		
		(0.000222)		
Relative emissions targets (dummy)				0.0248
				(0.0303)
Policy density * Relative emissions targets				-0.000770***
				(0.000245)
**CONTROLS**				
Rule of law	-0.0374	-0.0472	-0.0413	-0.0342
	(0.0301)	(0.0291)	(0.0303)	(0.0303)
Hodrick-Prescott GDP filter	-0.354	-0.284	-0.334	-0.382
	(0.249)	(0.242)	(0.247)	(0.248)
GDP per capita log	3.735***	3.245***	3.632***	4.012***
	(0.288)	(0.269)	(0.300)	(0.325)
GDP per capita log squared	-0.200***	-0.178***	-0.195***	-0.212***
	(0.0153)	(0.0143)	(0.0159)	(0.0167)
Imports share to GDP	-0.00410***	-0.00339***	-0.00409***	-0.00401***
	(0.000647)	(0.000642)	(0.000647)	(0.000650)
Services share to GDP	-0.00782***	-0.00781***	-0.00756***	-0.00674***
	(0.00220)	(0.00214)	(0.00220)	(0.00221)
Temperature variation	-0.0176**	-0.0154**	-0.0199**	-0.0176**
	(0.00792)	(0.00767)	(0.00779)	(0.00790)
Constant	-17.94***	-15.25***	-17.44***	-19.58***
	(1.318)	(1.233)	(1.388)	(1.560)
Observations	941	941	941	941
Years	2000-2022	2000-2022	2000-2022	2000-2022
R-squared	0.968	0.970	0.968	0.968
F test (for dummy and interaction term)		30.95		6.58
F test *P* value		0.000		0.0015
within R-squared	0.27	0.3175	0.2819	0.2785
RMSE	0.09	0.0891	0.0914	0.0916

Notes: Dependent variable is the log of emission intensity. Robust standard errors are reported in parentheses. *** p<0.01, ** p<0.05, * p<0.1

2.2 Roles of (inter)governmental organisations

Our models show the interaction effect between national climate policy portfolio effectiveness and membership of international organisations like the IEA, EU-EFTA, and CEM. Results are shown in full in Table S4, showing a significant and beneficial association in all three cases. We interpret these results with caution as the organisations do not play a direct role in domestic policy, and reverse causality cannot be ruled out (i.e., that countries with effective policy portfolios and progress in reducing emissions become members of intergovernmental organisations).

The 32 current member countries of the IEA are all in the OECD, so membership is an indirect measure of country development stage and income level, controlled for in our models by the GDP and GDP-squared terms. Originally the IEA was establish to represent the interests of oil-importing countries (following the 1970s OPEC embargo) but has more recently been active in promoting pathways, policies, and clean energy innovation programmes in line with net-zero goals [19]. The IEA has an explicit policy evaluation function in energy efficiency and demand, in clean energy innovation, in renewable energy and electrification (e.g., in the annual Energy Efficiency and Energy Technology Perspectives reports). The IEA's work also supports diffusion of policy learning and insights among its member countries.

The EU-EFTA dummy captures the interdependence between EU countries (and EFTA trading partners) through their transposition of EU legislation into national laws. This includes climate policies which we only consider at the national level in our measure of policy density.

Countries choose to join the CEM because they are already actively adopting and implementing ambitious climate policies or because they recognise the need to do so [11]. Becoming and remaining a member of the CEM requires a certain level of commitment through participation in workstreams considered relevant to the country's clean energy transition [20]. Some of the workstreams aim at improving the implementation (not the formulation) of domestic policies, but most of them are designed to facilitate public-private collaboration in the diffusion of clean energy technologies that can contribute to reducing GHG emissions. This effect may be mediated through domestic climate or clean energy policies, but it may also be a direct effect through technology diffusion. By testing the interaction between CEM membership and policy accumulation we capture a pathway to GHG emission intensity reduction that exists in addition to the policy pathway based on the diffusion of clean energy technology.

Table S4. (Inter)governmental organisations

VARIABLES	energy ministry (1) Log(CO2/GDP)	energy and/or climate ministry (2) Log(CO2/GDP)	CEM (3) Log(CO2/GDP)	IEA (4) Log(CO2/GDP)	EU-EFTA (5) Log(CO2/GDP)
Policy density	-0.000554***	-0.00110***	-0.000183	0.000354	-0.000232
	(0.000169)	(0.000206)	(0.000333)	(0.000328)	(0.000207)
Energy ministry (dummy)	-0.0447***				
	(0.0149)				
Policy density*Energy ministry	0.000690***				
	(0.000173)				
Energy and/or climate ministry (dummy)		-0.0471***			
		(0.0146)			
Policy density*Energy and/or climate ministry		0.000847***			
		(0.000178)			
Clean energy ministerial			0.0974***		
			(0.0183)		
Policy density*clean energy ministerial			-0.000719**		
			(0.000281)		
IEA (dummy)				-0.0670*	
				(0.0346)	
Policy density*IEA				-0.00109***	
				(0.000338)	
EU-EFTA (dummy)					-0.00659
					(0.0281)
Policy density*EU-EFTA					-0.00144***
					(0.000317)
**CONTROLS**					
Rule of law	-0.0383	-0.0363	-0.0352	-0.0372	-0.0437
	(0.0299)	(0.0299)	(0.0288)	(0.0291)	(0.0291)
Hodrick-Prescott GDP filter	-0.382	-0.376	-0.464*	-0.382	-0.258
	(0.248)	(0.247)	(0.246)	(0.249)	(0.249)
GDP per capita log	3.601***	3.679***	3.681***	2.989***	3.327***
	(0.289)	(0.302)	(0.296)	(0.353)	(0.290)
GDP per capita log squared	-0.193***	-0.196***	-0.197***	-0.165***	-0.181***
	(0.0153)	(0.0159)	(0.0156)	(0.0181)	(0.0153)
Imports share to GDP	-0.00392***	-0.00397***	-0.00365***	-0.00359***	-0.00311***
	(0.000644)	(0.000650)	(0.000643)	(0.000650)	(0.000694)
Services share to GDP	-0.00575**	-0.00541**	-0.00808***	-0.00984***	-0.00758***
	(0.00228)	(0.00227)	(0.00212)	(0.00234)	(0.00215)
Temperature variation	-0.0158**	-0.0149*	-0.0141*	-0.0153**	-0.0169**
	(0.00801)	(0.00791)	(0.00786)	(0.00760)	(0.00770)
Constant	-17.48***	-17.90***	-17.78***	-13.87***	-15.76***

	(1.321)	(1.389)	(1.371)	(1.734)	(1.352)
Observations	941	941	941	941	941
Years	2000-2022	2000-2022	2000-2022	2000-2022	2000-2022
R-squared	0.968	0.968	0.969	0.969	0.969
F test (for dummy and interaction term)	8.02	11.37	16.32	8.324	11.7
F test *P* value	0.0004	0	0.000	0.000	0.000
within R-squared	0.2855	0.2895	0.2988	0.2972	0.3087
RMSE	0.0912	0.0909	0.0903	0.0904	0.0897

Notes: Dependent variable is the log of emission intensity. Robust standard errors are reported in parentheses. *** p<0.01, ** p<0.05, * p<0.1

3 Control variables: selection & justification

In our models, we control for the effect of structural shifts in economic activity to the service sector while offshoring emission-intensive industrial production (carbon leakage). (All our models have imports share of GDP and services share of GDP as control variables, in addition to GDP per capita and its squared term).

From an econometric perspective, the inclusion of GDP per capita, services share, and import share aligns with standard practices in applied panel data analysis to block confounding pathways. In a two-way fixed-effects framework, controlling for observed time-varying confounders is necessary when the treatment is also time-varying and potentially correlated with omitted variables that affect the outcome [21,22]. As emphasised by Cinelli and Hazlett [23], even in high-dimensional settings, control strategies grounded in causal assumptions help prevent omitted variable bias and enhance identification of causal effects. We use directed acyclic graphs (DAGs) to explain and justify the role of our control variables [24-26].

3.1 Directed Acyclic Graphs (DAGs): confounders & backdoor paths

We present the basic DAG in Figure S4a and then discuss the causal assumptions and roles of each variable.

Figure S4. Directed acyclic graph (DAGs) of our research design: left panel [a] shows confounders; right panel [b] shows secondary controls.

[a]

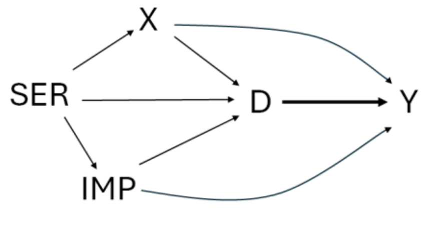

[b]

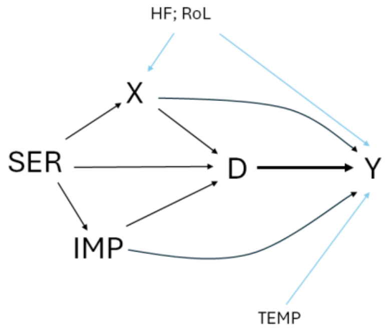

In our model, Y denotes the outcome (dependent) variable i.e., emission intensity; D is the treatment (independent) variable capturing policy density; X represents GDP per capita; SER indicates the share of the services sector in the economy; and IMP denotes the share of imports; HF represents the Hodrick-Prescott filter; RoL denotes the Rule of Law index; and TEMP denotes the temperature variation from the long-term mean.

The direct path from D to Y captures the causal effect of interest: the impact of policy density (D) on emission intensity (Y):

(D) Policy Density → (Y) Emission Intensity

To obtain an unbiased estimate of the causal effect, it is necessary to block the following backdoor paths using appropriate controls pp99-102 in [26,]pp79-85 in [27]. These paths (Figure S4a) represent non-causal associations that could bias the estimation of the treatment effect:

1. D ← X → Y
2. D ← SER → X → Y
3. D ← SER → IMP → X → Y
4. D ← SER → IMP → Y

Backdoor path 1:

1. D ← X → Y, in which X is GDP per capita (including its square term).

This backdoor path introduces spurious correlations between D and Y due to variation in the confounder X that affects both D and Y. Here the confounder X is GDP per capita and its squared term, a proxy for a country's development stage. Including the GDP squared term captures the non-linear (U-shaped) effect of development stage on emission intensity. In our study, climate policy density serves as a proxy for this transition so we expect a similar nonlinear relationship as for emission intensity. GDP per capita is a confounder as it correlates with the both the treatment and the outcome although in a non-causal way (see also Table S6).

Backdoor path 2:

2. D ← SER → X → Y, in which SER is the share of services in economic activity.

Controlling for GDP per capita closes one backdoor path, but introduces a second backdoor path from D to Y that arises due to variation in the share of economic activity in the services sector (SER) which is therefore a potential confounder. A higher services share is closely associated with a country's level of development. More developed economies tend to have larger services sectors that yield greater value-added activity than manufacturing or agriculture [28]. In addition, specialisation in services such as real estate, finance, and insurance prompts regulatory oversight that contributes to higher policy density. The delivery of services typically involves complex transactions and institutional arrangements, requiring more sophisticated regulatory environments to ensure efficiency, stability, and consumer protection [29]. This is also shown in Table S6.

To close this second backdoor path and avoid omitted variable bias, we control for the share of the services sector in the economy. Structural change within the services sector could also directly reduce emission intensity, although to a lesser extent than the backdoor path shown in the DAG that we account for in our approach.

Backdoor paths 3 and 4:

3. D ← SER → IMP → X → Y, in which IMP is the share of imports in a country's economy.
4. D ← SER → IMP → Y

Controlling for services introduces two additional backdoor paths that must be addressed to avoid bias. First, specialisation in services can generate comparative trade advantages for these types of exports, by extension increasing the share of imports of goods, specifically of manufactured products. Policy density may then be jointly influenced by rising services-

sector specialisation and increased goods imports as governments respond to trade practices (e.g. dumping) by introducing protective regulations to safeguard domestic industries. As Rodrik [30] argues, trade agreements become more about domestic rules and regulations than tariffs and non-tariff barriers.

Second, increased reliance on services-based economic activity and corresponding imports of manufactured goods is associated with reduced domestic emission intensity. This reflects a form of carbon leakage through which more emission-intensive production is offshored to developing countries. In recent work Eskander and Fankhauser [31] use two-way fixed effect models on both production-based and consumption-based emissions to show climate policy has not resulted in trade-related carbon leakage from importing to exporting countries.

In our model, controlling for imports closes two backdoor paths: between services and policy density, and between services and emission intensity.

3.2 Bad controls & colliders

According to Cunningham [26], it is common for a collider to entirely flip the sign of the coefficient of interest when introduced in the model: "*Angrist and Pischke (2009) talk about this problem in a different way using language called "bad controls". Bad controls are not merely conditioning on outcomes. Rather, they are any situation in which the outcome had been a collider linking the treatment to the outcome of interest.*"

Starting with a model specification without fixed effects and including only our treatment variable, we can see in column1-Table S5 that the coefficient for policy density is negative and significant as we expect. Controlling for country effects in column 2-Table S5, the coefficient remains negative and significant. These first two models are called 'biased unconditional' in a relevant example used in Cunningham [26], p110.

Once we introduce year fixed effects in column 3-Table S5 the coefficient flips its sign to become positive. This is not reasonable and the equivalent model in Cunningham [26], pp106-110 is called 'biased biased'. Year dummies controlling for time fixed effects effectively function as a collider in our case. This is because when we control for time, we introduce a backdoor path for policy density and emission intensity that is spurious and distorts the nature of the causal relationship. Policy density tends to increase over similar time periods for group of countries that are part of political unions and common economic markets. In the EU, for example: "More than half of the national measures are EU regulations. In some cases, the ratio is such that EU regulations account for 80 percent of national climate change policies (Netherlands and Belgium)" [32]. If policy density increases at similar rates in specific years for groups of countries within our sample, then simple models of emission intensity reduction while controlling for yearly effects will understate the effect of policy density (e.g., the baseline parsimonious TWFE model in column 3-Table S5).

To counteract this, we first need to introduce the confounder discussed above that accounts for countries' development stage (column 4-Table S5). This confounder captures better underlying variation among countries within the same group and accounts for additional nuances in the effect over policy density conditional on countries' economic status. We can observe that the coefficient for policy density in column 4 becomes negative, as one would expect, although it remains statistically non-significant. This model is still 'biased biased' because we haven't yet controlled for all backdoor paths opened due to the introduction of the new confounder variable (see above). Introducing the additional two confounders that account for share of services and share of imports, we cut off all backdoor paths and obtain a negative and statistically significant coefficient for policy density. The model in column 6-Table S5 is the one that Cunningham [26], p110 calls 'unbiased conditional'.

In terms of standard errors variation, we can observe that there is minimal variation between column 3 (0.000149) and column 6 (0.000154). When we introduce the final controls, we observe a small correction in the magnitude of the coefficient, and minimal change in its standard error variation (0.000163 in column 7 and 8, and 0.000169 in column 9).

Our sequential inclusion of controls mirrors the logic of a Frisch-Waugh-Lovell (FWL) decomposition, whereby we partial out the effect of other covariates to isolate the variation in policy density that is orthogonal to plausible confounders. The modest changes in the coefficient magnitude and standard errors in later model stages (columns 6–9 of Table S5 below) suggest that the control set reduces bias without introducing excessive multicollinearity or instability. This pattern is consistent with an "unbiased conditional" specification.

We provide additional sensitivity analysis in Table S6 that further supports our reasoning. Once we drop time fixed effects, the coefficient for policy density always remains negative and statistically significant. This indicates that the change observed in Table S5 is due to the discussed collider effect, and by mainly shutting off backdoor pathways, we obtain 'unbiased conditional' estimates. Introducing controls one by one in the parsimonious model without time fixed effects further confirms our expectations based on DAG formation presented in Figure S4a and confirms findings for model specification including time fixed effects. We can also see that the coefficients for policy density are in general larger in terms of magnitude when excluding time fixed effects in Table S6 than the corresponding ones in Table S5. The larger coefficients without time fixed effects represent overestimated effects that include spurious correlation from shared time trends. In other words, omitting time fixed effects would fail to control for unobserved variation in emission intensities from 2000 to 2022, leading to biased model estimates.

3.3 Secondary control variables & potential backdoor paths: business cycles & rule of law

We include a set of secondary controls in our DAG shown in Figure S4b, which are not directly related to policy density, but may potentially have a spurious influence on the identified direct treatment effect if left uncontrolled. These variables are not part of the main DAG pathways shown in Figure S4a. They help ensure robustness by capturing broader contextual variation not absorbed by our main controls or fixed effects.

More specifically, introducing GDP per capita may raise concerns about opening alternative backdoor paths related to cyclical fluctuation in the business cycle, and variation in policy-relevant institutions. To capture the former, we introduce the Hodrick-Prescott GDP filter that is commonly used to account for cyclical volatility in economic activity. To capture institutional 'quality', we include the World Bank's rule of law index from the Worldwide Governance Indicators (WGI) project. There are alternative indicators for measuring rule of law that are highly correlated [33]. Eskander and Fankhauser [31] use a basket of 6 indices to control for institutional quality including both rule of law and government effectiveness. Best, et al. [34] use government effectiveness in their models and similarly find it is not associated with policy density.

The rule of law index varies across countries and time [35]. Although institutions are typically slow-moving and path-dependent, the 2000–2022 period includes meaningful institutional shifts in several countries particularly within the BRIICS including through crises, regime changes, democratisation, autocratization, and reforms (see Figure S5). Considering this variation, we include Rule of Law as a control to account for institutional differences across our country sample that may impact the effectiveness with which climate policy density affects emission intensities (e.g., through changes to policy credibility, monitoring, and enforcement).

We expect both these secondary controls to be correlated with GDP per capita and emission intensity, and not to be significantly associated with policy density. This is indeed the case (see Table S7).

3.4 Secondary control variables: temperature variation

Finally, we introduce temperature anomalies from the long-term mean in our model to account for climate variability, given that it might not be fully captured by year fixed effects, which account only for linear time trends. Recent data from NASA (https://svs.gsfc.nasa.gov/5452) show an increase in both the frequency and intensity of temperature anomalies over the last decade, with the warming trend accelerating at different rates across countries.

Temperature variation is exogenous to policy density (the treatment variable) and helps capture anomalies that could be associated with emission intensity fluctuations. It is not plausibly influenced by policy density, and its inclusion strengthens the robustness of our estimates without introducing endogeneity.

to ensure the robustness of our research design, so we follow the reviewer's suggestion to regress temperature variation on policy density (Table S9, column 1). As expected, results appear statistically significant, which we interpret as a model misspecification. To correct for accelerating trends and potential heteroskedasticity (particularly relevant given increasing temperature variability across countries in recent years), we log-transform temperature variation. This log-linearised model confirms that the coefficient for temperature variation is statistically non-significant, as expected, thereby supporting the exogeneity of this control.

To further support our argument, we re-estimate the model using as dependent variable the log of policy density and as independent variable (i) temperature variation (Table S9, column 3), and (ii) the log of temperature variation (Table S9, column 4). The absent of significant evidence in both models confirms that once either or both variables are log-transformed, temperature variation shows no systematic association with policy density.

To further test for robustness, we examine whether the inclusion of temperature variation affects identification of the treatment effect. Specifically, we compare the coefficient for policy density in the full model with temperature variation to the corresponding coefficient in the reduced model without temperature variation using an F-test of coefficient equality. We estimate χ^2 statistic equal to 0.70 with *p*-value 0.40. This means that the test fails to reject the null hypothesis of no difference, suggesting that including temperature variation in the model does not significantly alter the estimated treatment effect. This supports our decision to include it as a control without compromising causal identification. This is also consistent with the precedents on which we build and so facilitates comparability of our model estimates with prior studies [6,31].

3.5 Secondary control variables & fixed effects

To demonstrate our secondary controls are distinct from the unit (country) fixed effect, we computed and report both the overall standard deviation and the within-unit standard deviation (i.e., fixed-effects-residualised) following Mummolo and Peterson [36]. This allows us to examine the proportion of meaningful variation that is actually used for identification in the fixed effects models. Table S8 follows the structure used in [36] to show within-unit variation for Rule of Law. While reduced, it remains meaningful (retaining 17% of its total variance), supporting our choice to include it in our models. Similarly, variables controlling for economic structure (GDP per capita and its squared term, services share, and imports share) retain on average 20% of their variation once we account for fixed effects. The treatment variable,

policy density, retains just under half of its variation, highlighting its suitability for our research design. As expected, secondary controls, Hodrick-Prescott filter and temperature variation retain over two-thirds of their variance, consistent with their role in capturing cyclical and exogenous fluctuations.

Table S5. Sensitivity analysis with sequential addition of controls to the baseline model

**VARIABLES**	**(1)** **LOG (CO2/GDP)**	**(2)** **LOG (CO2/GDP)**	**(3)** **LOG (CO2/GDP)**	**(4)** **LOG (CO2/GDP)**	**(5)** **LOG (CO2/GDP)**	**(6)** **LOG (CO2/GDP)**	**(7)** **LOG (CO2/GDP)**	**(8)** **LOG (CO2/GDP)**	**(9)** **LOG (CO2/GDP)**
Policy density)	-0.00127***	-0.00450***	0.000479***	-0.000177	-0.000247*	-0.000454***	-0.000514***	-0.000505***	-0.000554***
	(0.000388)	(0.000184)	(0.000149)	(0.000150)	(0.000148)	(0.000154)	(0.000163)	(0.000163)	(0.000169)
**CONTROLS**									
GDP per capita (log)				3.907***	4.258***	3.889***	3.866***	3.822***	3.735***
				(0.251)	(0.280)	(0.252)	(0.287)	(0.285)	(0.288)
GDP per capita (log) squared				-0.211***	-0.229***	-0.210***	-0.207***	-0.204***	-0.200***
				(0.0131)	(0.0147)	(0.0134)	(0.0151)	(0.0150)	(0.0153)
Services share of GDP					-0.00856***	-0.00948***	-0.00871***	-0.00896***	-0.00782***
					(0.00211)	(0.00211)	(0.00218)	(0.00221)	(0.00220)
Imports share of GDP						-0.00435***	-0.00434***	-0.00435***	-0.00410***
						(0.000611)	(0.000635)	(0.000637)	(0.000647)
Rule of law							-0.0424	-0.0436	-0.0374
							(0.0298)	(0.0298)	(0.0301)
Hodrick-Prescott GDP filter								-0.330	-0.354
								(0.254)	(0.249)
Temperature variation									-0.0176**
									(0.00792)
Constant	-1.544***	-1.411***	-1.616***	-19.25***	-20.43***	-18.41***	-18.52***	-18.34***	-17.94***
	(0.0213)	(0.00849)	(0.00723)	(1.199)	(1.288)	(1.143)	(1.329)	(1.323)	(1.318)
FE - Country	NO	YES	YES	YES	YES	YES	YES	YES	YES
FE - Year	NO	NO	YES	YES	YES	YES	YES	YES	YES
Observations	1,027	1,027	1,027	1,027	1,017	1,017	974	974	941
R-squared	0.013	0.890	0.954	0.964	0.965	0.967	0.967	0.967	0.968

Notes: Dependent variable is the log of emission intensity. Robust standard errors are reported in parentheses. *** p<0.01, ** p<0.05, * p<0.1

Table S6. Sensitivity analysis with sequential addition of controls to the baseline model excluding time fixed effects

VARIABLES	(1) LOG (CO2/GDP)	(2) LOG (CO2/GDP)	(3) LOG (CO2/GDP)	(4) LOG (CO2/GDP)	(5) LOG (CO2/GDP)	(6) LOG (CO2/GDP)	(7) LOG (CO2/GDP)	(8) LOG (CO2/GDP)
Policy density	-0.00127***	-0.00450***	-0.00272***	-0.00237***	-0.00220***	-0.00221***	-0.00217***	-0.00196***
	(0.000388)	(0.000184)	(0.000165)	(0.000155)	(0.000141)	(0.000155)	(0.000154)	(0.000153)
**CONTROLS**								
GDP per capita (log)			6.324***	6.710***	5.580***	5.690***	5.727***	5.267***
			(0.313)	(0.314)	(0.283)	(0.303)	(0.303)	(0.299)
GDP per capita (log) squared			-0.347***	-0.364***	-0.303***	-0.309***	-0.312***	-0.287***
			(0.0160)	(0.0158)	(0.0148)	(0.0157)	(0.0158)	(0.0156)
Services share of GDP				-0.0177***	-0.0175***	-0.0180***	-0.0163***	-0.0146***
				(0.00225)	(0.00223)	(0.00238)	(0.00237)	(0.00228)
Imports share of GDP					-0.00662***	-0.00647***	-0.00669***	-0.00593***
					(0.000690)	(0.000716)	(0.000709)	(0.000652)
Rule of law						0.0516*	0.0539*	0.0345
						(0.0296)	(0.0296)	(0.0297)
Hodrick-Prescott GDP filter							0.665***	0.715***
							(0.206)	(0.202)
Temperature variation								-0.0503***
								(0.00763)
Constant	-1.544***	-1.411***	-29.42***	-30.55***	-25.22***	-25.75***	-25.90***	-23.88***
	(0.0213)	(0.00849)	(1.526)	(1.520)	(1.313)	(1.413)	(1.412)	(1.387)
FE - Country	NO	YES	YES	YES	YES	YES	YES	YES
FE - Year	NO	NO	NO	NO	NO	NO	NO	NO
Observations	1,027	1,027	1,027	1,017	1,017	974	974	941
R-squared	0.013	0.890	0.944	0.949	0.955	0.955	0.956	0.960

Notes: Dependent variable is the log of emission intensity. Robust standard errors are reported in parentheses. *** p<0.01, ** p<0.05, * p<0.1

Table S7. Sensitivity analysis: testing for associations between treatment variable (policy density) and secondary control variables.

VARIABLES	(1) Policy density	(2) Policy density	(3) GDP per capita	(4) GDP per capita
Hodrick-Prescott GDP filter	0.612		1.606***	
	(42.58)		(0.278)	
Rule of law		-6.795		0.345***
		(6.513)		(0.0340)
Constant	41.17***	49.74***	10.51***	10.18***
	(0.645)	(6.317)	(0.00364)	(0.0334)
FE - Country	YES	YES	YES	YES
FE - Year	YES	YES	YES	YES
Observations	1,028	989	1,028	989
R-squared	0.797	0.807	0.969	0.974

Notes: Dependent variable in models (1)-(2) is policy density, and in models (3)-(4) is GDP per capita. Robust standard errors are reported in parentheses. *** p<0.01, ** p<0.05, * p<0.1

Table S8. Standard deviation of independent variables before and after controlling for fixed effects

	(1) Coefficient	(2) SD	(3) Within unit SD	(4) Δ%
Policy density	-0.000554	44.95	20.24	-54.99
GDP per capita (log)	3.735	0.64	0.12	-81.83
GDP per capita (log) squared	-0.200	13.12	2.25	-82.87
Services share to GDP	-0.00782	7.71	1.67	-78.31
Imports share to GDP	-0.00410	25.86	5.36	-79.27
Rule of Law	-0.0374	0.83	0.13	-83.80
Hodrick-Prescott GDP filter	-0.352	0.019	0.013	-33.45
Temperature variation	-0.0176	0.52	0.40	-23.70

Notes: Column 1 shows the coefficients estimated in our base policy density model including all control variables. Column 2 shows the standard deviation of the independent variables for the full sample. Column 3 shows the within-unit standard deviation meaning that variables are standardised for country and year fixed effects[36]. Column 4 show the % difference (Δ) between standard deviations reported in columns 3 and 2.

Table S9. Sensitivity analysis: testing for associations between treatment variable (policy density) and control variable for temperature variation from long-term mean.

VARIABLES	(1) Policy density	(2) Policy density	(3) LOG (Policy density)	(4) LOG (Policy density)
Temperature variation	-4.775***		-0.0285	
	(1.469)		(0.0206)	
Temperature variation (log)		-0.783		0.00529
		(0.799)		(0.0118)
Constant	40.03***	44.71***	3.106***	3.325***
	(0.686)	(1.193)	(0.0102)	(0.0174)
FE - Country	YES	YES	YES	YES
FE - Year	YES	YES	YES	YES
Observations	989	620	968	611
R-squared	0.797	0.845	0.945	0.950

Notes: The dependent variable in columns 1 and 2 is policy density; in columns 3 and 4, it is the log of policy density. Robust standard errors are reported in parentheses. *** p<0.01, ** p<0.05, * p<0.1.

Figure S5. Variation in Rule of Law index 2000-2022 in BRIICS, with UK as comparison.

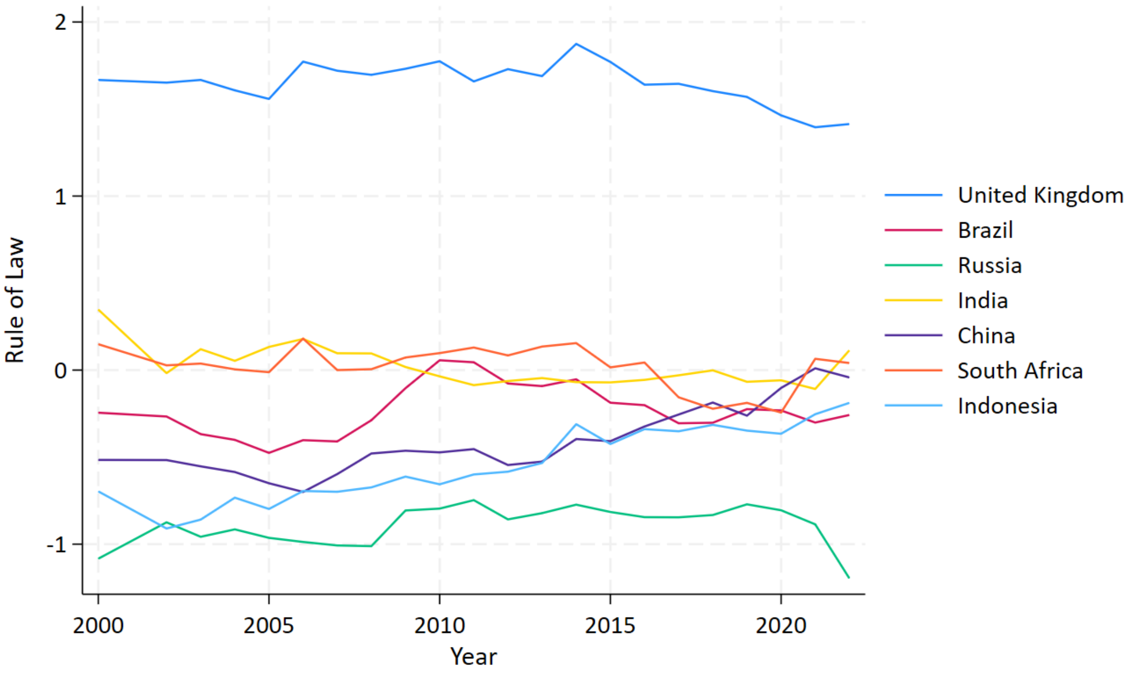

4 Policy density models: interpreting effect sizes

Our treatment variable, policy density, is a count variable that captures the accumulation of discrete policies. Consequently we prefer to present effect sizes in terms of a one-unit change, following [36]: "*Some might wish to convey treatment effects in the common scenario of a one-unit shift. This may be especially useful if the treatment being studied is dichotomous or a count variable that takes only integer values …*"

This is also consistent with prior studies that express effect sizes in relation to unit increases in policy density [6,31,37].

However, standardising coefficients using the within-group standard deviation can improve interpretability, particularly when comparing effect sizes across variables measured on different scales. We implement the standardisation procedure proposed by [36]. We residualise each predictor with respect to country and year fixed effects, calculate the within-unit standard deviation of the residuals, and use these to standardise the independent variables before re-estimating the model.

Results of this re-estimated model are presented in Table S10 below. We find that a 1 standard deviation increase in policy density (equivalent to 20 polices, see Table S8) is associated with a 1.1% reduction in emission intensity. As shown in Table S18, the standardised effect of policy density is comparable in magnitude to that of key confounders (GDP per capita, services share, imports share), and substantially larger than that of secondary controls (rule of law, Hodrick-Prescott filter, temperature variation).

Table S10. Standardised coefficients using the within-group standard deviation

VARIABLES	(1)<br>Original Coefficients<br>(Base Model)	(2)<br>Standardised Coefficients<br>(within SD)
Total policy density	-0.000554	-0.0112
GDP per capita (log)	3.735	0.4369
GDP per capita (log) squared	-0.200	-0.4493
Services share to GDP	-0.00782	-0.0131
Imports share to GDP	-0.00410	-0.0220
Rule of law	-0.0374	-0.0050
Hodrick-Prescott GDP filter	-0.352	-0.0046
Temperature variation	-0.0176	-0.0070

Column 1 shows the coefficients for the model specification in the base policy density model (main text model 1 in Table 1). Column 2 shows the standardised coefficients using the within-group standard deviation as proposed by [38].

5 Policy density models: robustness checks

5.1 Replicating Eskander and Fankhauser (2020)

We directly replicate the model of Eskander and Fankhauser [6] with both short-term and long-term policy density variables but applied to our smaller country sample, later time period, and larger policy sample. Full results provided below in Table S11. The results of our base model (model 1 in main text) were very similar.

Table S11. Replicating Eskander and Fankhauser (2020)

VARIABLES	(1) Log(CO2/GDP)
(L1) Total policy density	-0.000515***
	(0.000183)
(L1) Policy density last 3 years	-0.00127**
	(0.000545)
**CONTROLS**	
(L1) Rule of law	-0.0115
	(0.0314)
(L1) Hodrick-Prescott GDP filter	-0.0525
	(0.256)
(L1) GDP per capita log	3.917***
	(0.318)
(L1) GDP per capita log squared	-0.207***
	(0.0166)
(L1) Imports share to GDP	-0.00459***
	(0.000698)
(L1) Services share to GDP	-0.00857***
	(0.00221)
(L1) Temperature variation	-0.00557
	(0.00812)
Constant	-19.07***
	(1.511)
Observations	893
Years	2000-2023
R-squared	0.968
within R-squared	0.2394
RMSE	0.093

Notes: Dependent variable is the log of emission intensity. Robust standard errors are reported in parentheses.
*** p<0.01, ** p<0.05, * p<0.1

5.2 Pre-2000 policies

The policy density variable included in our analysis measures the cumulative number of policies implemented from 2000 onwards. Initial policy densities in all countries are set to zero in 2000. Although most policies have been implemented since then, some countries including Australia, Denmark, Finland, Korea, and Norway had introduced significant numbers of policies before 2000.

As a robustness check, we incorporate in our policy density variable the cumulative number of policies implemented before 2000 and re-estimate the baseline regression model. Results provided in Table S12, column 1 show that the size and statistical significance of all coefficients in the re-estimated model remain the same.

Table S12. Robustness checks of our baseline model

VARIABLES	(1)<br>Including pre-2000 policies<br>LOG<br>(CO2/GDP)	(2)<br>Excluding China<br>LOG<br>(CO2/GDP)
Policy density	-0.000554***	-0.000527***
	(0.000169)	(0.000170)
**CONTROLS**		
Rule of law	-0.0374	-0.0328
	(0.0301)	(0.0304)
Hodrick-Prescott GDP filter	-0.354	-0.375
	(0.249)	(0.250)
GDP per capita log	3.735***	3.630***
	(0.288)	(0.284)
GDP per capita log squared	-0.200***	-0.194***
	(0.0153)	(0.0152)
Imports share to GDP	-0.00410***	-0.00399***
	(0.000647)	(0.000652)
Services share to GDP	-0.00782***	-0.00729***
	(0.00220)	(0.00220)
Temperature variation	-0.0176**	-0.0184**
	(0.00792)	(0.00798)
Constant	-17.94***	-17.53***
	(1.318)	(1.291)
Observations	941	919
Years	2000-2022	2000-2022
R-squared	0.968	0.964
within R-squared	0.2728	0.2616
RMSE	0.0919	0.0921

Notes: Dependent variable is the log of emission intensity. Robust standard errors are reported in parentheses.
*** p<0.01, ** p<0.05, * p<0.1

5.3 Excluding China

Given our policy density variable is based on the IEA Policies and Measures Database (PMD), it has a different distribution to the same variable used in Eskander & Fankhauser (2020) which is based on the Climate Change Laws of the World (CCLW) database. This can be clearly observed in the case of China for which the IEA PMD reports at least 179 policies for the period 2000 - 2016 compared to only 5 legislations reported in the Climate Change Laws of the World database over the same time-period.

A similar pattern of differences between the two datasets can be observed for the rest of the BRIICS. More specifically, the IEA PMD reports 32 polices for Brazil, 32 Russia, 80 for India, 53 for South Africa, and 63 for Indonesia. In contrast, the CCLW reports 18 policies for Brazil, 12 for Russia, 9 for India, 7 for South Africa, and 18 for Indonesia.

We check whether our results change when we remove China from our sample. Results provided in Table S12, column 2 show that the coefficient for policy density remains negative and statistically significant.

5.4 Excluding Costa Rica

We do not include Costa Rica which joined the OECD in 2021. We ran robustness checks on our base model with Costa Rica included but found low variation in key variables affected the stability of results (e.g., Costa Rica has no policies in the industry sector).

5.5 One year lagged policy density

We perform an additional robustness test on our base model in which all independent variables are lagged by one year (Table S13, column 1), similar to [6]. All the coefficients in the model with lagged (t-1) parameters are very similar to those in the base model, but with a slightly larger effect for policy density on emission intensity.

Table S13. Independent variables are lagged by one year.

	**one year lagged**
	**(1)**
**VARIABLES**	**LOG(CO2/GDP)**
(L1) Policy density	-0.000559***
	(0.000170)
**CONTROLS**	
(L1) Rule of law	-0.0187
	(0.0300)
(L1) Hodrick-Prescott GDP filter	-0.0150
	(0.252)
(L1) GDP per capita log	3.740***
	(0.290)
(L1) GDP per capita log squared	-0.200***
	(0.0154)
(L1) Imports share to GDP	-0.00483***
	(0.000677)
(L1) Services share to GDP	-0.00880***
	(0.00216)
(L1) Temperature variation	-0.00160
	(0.00786)
Constant	-17.60***
	(1.389)
Observations	936
Years	2000-2022
R-squared	0.967
within R-squared	0.2537
RMSE	0.0942

Notes: Dependent variable is the log of emission intensity. Robust standard errors are reported in parentheses.
*** p<0.01, ** p<0.05, * p<0.1

5.6 Shannon Index diversity measures and 2020 Covid impact

The Shannon Index (SNI) is an alternative measure of diversity to the Herfindahl-Hirschman Index (HHI). The SNI is more commonly used in ecology but also applied in economics, e.g. employment distribution among industrial sectors [39]. The SNI takes negative values, and is unbounded, so 0 means absolute specialisation and *-n* represent a lower level of specialisation. In absolute terms, a higher value of the SNI indicates higher diversity, i.e., lower concentration.

In our models a negative coefficient on SNI is interpreted in the same way as a negative coefficient on HHI because the SNI is the opposite in two ways: negative not positive scale; higher magnitude value = lower concentration not higher concentration.

We construct the SNI for each country i by taking for each year t the sum of the product of the share of policy instrument types s_{jt} (or sectors covered) within total policy density and the natural logarithm of this share ($ln(s_{jt})$). By using total policy density, we control for the cumulative (long-term) effect of specialisation on changes in fossil CO_2 emissions.

$$Shannon_{it} = -\sum_{j=1}^{n} s_{jt} ln(s_{jt})$$

We report full model results using the SNI instead of the HHI in Table S14. Coefficients for the SNI are negative and significant as expected.

The SNI is less sensitive than the HHI to the anomalous share of sectoral emissions in 2020 due to the Covid impact on transport sector emissions. This 2020 anomaly causes the coefficient for our HHI variable measuring policy sectoral coverage weighted by sectoral emissions to become non-significant for the period 2000-2022.

We use the SNI results to interpret the loss in significance in our HHI diversity measure of emission-weighted policy sectoral coverage. Over the period 2000-2019, the emission-weighted HHI diversity measure had a significant negative coefficient, in line with expectations: policy portfolios weighted towards high emitting sectors are more effective. However, over the period to 2022, this coefficient becomes non-significant. Our interpretation is that the sectoral shares of emissions in 2020 deviates from the longer-term trend, for example, due to the drop in transport sector emissions under lockdown-related travel restrictions. Our main diversity measure, the HHI, is sensitive to anomalous changes in sectoral shares. This causes the HHI diversity measure of emission-weighted policy sectoral coverage to drop in significance (shown in Table S15).

The alternative SNI diversity measure is less sensitive to anomalous changes in sectoral shares, and remains negative and statistically significant both over the 2000-2019 period, and the full 2000-2022 period (shown in Table S14).

Otherwise, the major impact of Covid-related disruptions in 2020 (and to a lesser extent in 2021) affect both total CO_2 emissions and GDP in our dependent variable, so our models of emission intensity are robust to the Covid discontinuity [40].

Table S14. Augmented models with Shannon Index diversity measures

**VARIABLES**	**(1)**<br>**Robustness check LOG (CO2/GDP)**	**(2)**<br>**Robustness check LOG (CO2/GDP)**	**(3)**<br>**Robustness check LOG (CO2/GDP)**	**(4)**<br>**Robustness check LOG (CO2/GDP)**
Policy density	-0.000650***	-0.000686***	-0.000496***	-0.000534***
	(0.000169)	(0.000164)	(0.000168)	(0.000165)
Shannon 6 instrument types	-0.0586***			
	(0.0201)			
Shannon 3 instrument categories		-0.0656**		
		(0.0256)		
Shannon sectoral - emissions weighted			-0.387***	
			(0.122)	
Shannon sectoral				-0.0700***
**CONTROLS**				
Rule of law	-0.0553*	-0.0553*	-0.0588**	-0.0537*
	(0.0291)	(0.0294)	(0.0298)	(0.0295)
Hodrick-Prescott GDP filter	-0.388	-0.432*	-0.304	-0.321
	(0.242)	(0.242)	(0.241)	(0.242)
GDP per capita log	3.662***	3.756***	3.827***	3.878***
	(0.302)	(0.296)	(0.287)	(0.286)
GDP per capita log squared	-0.195***	-0.200***	-0.204***	-0.207***
	(0.0160)	(0.0157)	(0.0152)	(0.0152)
Imports share to GDP	-0.00389***	-0.00384***	-0.00353***	-0.00362***
	(0.000627)	(0.000627)	(0.000639)	(0.000628)
Services share to GDP	-0.00667***	-0.00682***	-0.00700***	-0.00678***
	(0.00215)	(0.00217)	(0.00214)	(0.00215)
Temperature variation	-0.0231***	-0.0228***	-0.0195**	-0.0205***
	(0.00784)	(0.00782)	(0.00773)	(0.00777)
Constant	-17.84***	-18.31***	-18.65***	-18.87***
	(1.396)	(1.362)	(1.323)	(1.318)
Observations	919	919	923	923
Years	2000-2022	2000-2022	2000-2022	2000-2022
R-squared	0.969	0.969	0.969	0.969
within R-squared	0.28	0.2784	0.2787	0.2747
RMSE	0.0894	0.0895	0.0893	0.0895

Notes: Dependent variable is the log of emission intensity. Robust standard errors are reported in parentheses. *** p<0.01, ** p<0.05, * p<0.1

Table S15. Augmented models with Herfindahl-Hirschman Index diversity measures

**VARIABLES**	**(1)** **Model (2) in main text LOG (CO2/GDP)**	**(2)** **LOG (CO2/GDP)**	**(3)** **Model (3a) in main text LOG (CO2/GDP)**	**(4)** **Model (3b) in main text LOG (CO2/GDP)**	**(5)** **Model (3c) in main text LOG (CO2/GDP)**
Policy density	-0.000688***	-0.000702***	-0.000594***	-0.000673***	-0.000562***
	(0.000166)	(0.000162)	(0.000164)	(0.000161)	(0.000182)
HHI 6 instrument types	-0.114***				
	(0.0407)				
HHI 3 instrument categories		-0.109***			
		(0.0418)			
HHI sectoral - emissions weighted				-0.195	-0.252**
				(0.128)	(0.109)
HHI sectoral			-0.124**		
			(0.0513)		
**CONTROLS**					
Rule of law	-0.0547*	-0.0545*	-0.0589**	-0.0530*	-0.0170
	(0.0292)	(0.0293)	(0.0299)	(0.0305)	(0.0305)
Hodrick-Prescott GDP filter	-0.407*	-0.437*	-0.334	-0.379	-0.372*
	(0.242)	(0.242)	(0.243)	(0.244)	(0.220)
GDP per capita log	3.730***	3.754***	3.847***	3.851***	2.945***
	(0.298)	(0.297)	(0.290)	(0.300)	(0.307)
GDP per capita log squared	-0.199***	-0.200***	-0.205***	-0.204***	-0.160***
	(0.0157)	(0.0157)	(0.0153)	(0.0158)	(0.0171)
Imports share to GDP	-0.00386***	-0.00384***	-0.00373***	-0.00372***	-0.00264***
	(0.000633)	(0.000627)	(0.000623)	(0.000623)	(0.000673)
Services share to GDP	-0.00673***	-0.00685***	-0.00679***	-0.00697***	-0.00206
	(0.00215)	(0.00217)	(0.00215)	(0.00218)	(0.00227)
Temperature variation	-0.0231***	-0.0229***	-0.0206***	-0.0218***	-0.0152**
	(0.00781)	(0.00781)	(0.00778)	(0.00779)	(0.00648)
Constant	-18.08***	-18.20***	-18.63***	-18.72***	-14.50***
	(1.378)	(1.368)	(1.335)	(1.386)	(1.344)
Observations	919	919	923	923	795
Years	2000-2022	2000-2022	2000-2022	2000-2022	2000-2019
R-squared	0.969	0.969	0.969	0.969	0.975
within R-squared	0.2797	0.2786	0.272	0.2678	0.1915
RMSE	0.0895	0.0895	0.0897	0.0899	0.0788

Notes: Dependent variable is the log of emission intensity. Robust standard errors are reported in parentheses. *** p<0.01, ** p<0.05, * p<0.1

5.7 Absolute emissions as dependent variable

Ultimately climate policy has to reduce absolute levels of emissions. We use emission intensity as our dependent variable following the precedent in similar attribution studies [6] because it accounts for changes in emissions driven by a country's overall economic performance and size. (GDP and CO_2 are correlated at 92% in our sample). Using emission intensity rather than absolute emissions therefore helps reduce confounding factors when interpreting the effect of climate policy.

We run additional models reported in full in Table S16 for which we use the natural logarithm of absolute CO_2 emissions as the dependent variable instead of emissions intensity. We start by comparing our base model (Table 4 in main text, column 1) with a new model specification with ln CO_2 as the dependent variable (column 1, Table S16). The independent and control variables remain the same across both models. Policy density remains negative and statistically significant with a smaller (in absolute terms) coefficient compared to the base model. This is reasonable from an econometric perspective, as this model is mis-specified due to the absence of controls for economic activity, the primary driver of emissions. To address this misspecification, we include the natural logarithm of GDP as an additional control variable (column 2, Table S16). Again, results remain consistent, with the coefficient for total policy density slightly larger in magnitude than in the baseline model.

For the interaction effect between policy density and absolute emission-reduction targets on absolute levels of CO_2 emissions, we add this in (column 3, Table S16). The coefficient for the interaction effect remains strongly significant, similar to in our base model with emission intensity as the dependent variable (main text, and Table S3).

As additional sensitivity tests, we replace the natural logarithm of GDP with the natural logarithm of population as an alternative control variable in our models (columns 4 & 5, Table S16). Again, results remain remarkably consistent. The coefficients for policy density and the interaction effect with long-term targets are slightly larger in magnitude than the models with GDP as a control variable.

Overall, we find that policy density is associated with more rapid emission reductions regardless of whether emission intensity or absolute emissions are specified as the dependent variable in our models.

Table S16. Models with absolute emissions (CO2) as dependent variable

VARIABLES	(1)<br>LOG (CO2)	(2)<br>LOG (CO2)	(3)<br>LOG (CO2)	(4)<br>LOG (CO2)	(5)<br>LOG (CO2)
Policy density	-0.000452**	-0.000581***	-6.94e-05	-0.000599***	-4.65e-05
	(0.000187)	(0.000169)	(0.000183)	(0.000180)	(0.000196)
Absolute with 1990 baseline emission targets (dummy)			0.0142		0.0151
			(0.0212)		(0.0209)
Total policy density * Absolute with 1990 baseline emission targets			-0.00164***		-0.00177***
			(0.000219)		(0.000219)
**CONTROLS**					
Rule of law	-0.105***	-0.0279	-0.0523*	-0.0401	-0.0632**
	(0.0357)	(0.0289)	(0.0285)	(0.0288)	(0.0284)
Hodrick-Prescott GDP filter	-0.0782	-0.384	-0.254	-0.265	-0.149
	(0.280)	(0.248)	(0.242)	(0.256)	(0.248)
GDP per capita log	5.167***	3.599***	3.351***	4.739***	4.248***
	(0.284)	(0.320)	(0.280)	(0.292)	(0.254)
GDP per capita log squared	-0.224***	-0.198***	-0.179***	-0.200***	-0.179***
	(0.0151)	(0.0162)	(0.0141)	(0.0156)	(0.0135)
Imports share to GDP	-0.00469***	-0.00381***	-0.00321***	-0.00378***	-0.00313***
	(0.000788)	(0.000650)	(0.000628)	(0.000687)	(0.000657)
Services share to GDP	-0.00478**	-0.00783***	-0.00727***	-0.00699***	-0.00657***
	(0.00239)	(0.00219)	(0.00211)	(0.00214)	(0.00206)
Temperature variation	-0.0263***	-0.0177**	-0.0170**	-0.0191**	-0.0180**
	(0.00852)	(0.00791)	(0.00759)	(0.00807)	(0.00768)
GDP log		1.093***	0.899***		
		(0.0753)	(0.0739)		
Population log				0.983***	0.806***
				(0.0916)	(0.0906)
Constant	-17.09***	-10.50***	-8.767***	-18.17***	-14.84***
	(1.299)	(1.442)	(1.262)	(1.336)	(1.191)
Observations	941	941	941	941	941
Years	2000-2022	2000-2022	2000-2022	2000-2022	2000-2022
R-squared	0.997	0.997	0.998	0.997	0.998
F test (for Model B2 residuals)			36.24		40.1
F test *P* value			0		0
within R-squared	0.5577	0.6513	0.6738	0.6431	0.67
RMSE	0.5577	0.0909	0.088	0.092	0.0885

Notes: Dependent variable is the log of CO2 emissions. Robust standard errors are reported in parentheses. *** p<0.01, ** p<0.05, * p<0.1

5.8 Policy density by instrument type

Our study aim and approach is concerned with the cumulative effect of climate policy portfolios, including through the interaction with targets and governmental organisations. Although our models report clear and strong findings in each of these portfolio elements, our approach is not designed to identify the most effective policies *within* each national portfolio.

Our detailed policy vignettes for select countries provide a narrative account of policy accumulation that include discussions of effectiveness given each country's institutional context. The vignettes are summarised in Fig 3 in main text, and are explained in full in SI2 with supporting literature.

To explore policy effectiveness further, we run additional models for policy density by instrument type in order to see whether we could identify which instrument type was more effective in general terms (as opposed to in specific countries). We run a separate model including controls for policies coded in one of the three categories of instrument type:

- *regulatory*: includes legal interventions, monitoring, and regulations
- *economic*: includes market-based and direct provision
- *voluntary*: includes planning, informational, and other voluntary

Full results are shown in Table S17. Given that our dependent variable is emission intensity (CO_2/GDP), we did not expect to find a clear effect as the independent variable comprises only a subset of all policies (coded as being of a particular instrument type). However, two of the three models show negative and significant coefficients for the policy density by instrument type variable. The largest coefficient in absolute magnitude is for economic instruments, around twice that for regulatory and voluntary instruments.

The null result for regulations is surprising. Our interpretation is that regulations as an instrument type span a wide range from binding performance and emission standards to weaker reporting, safety, or compliance type regulations. Such regulations include, for example, safety and performance requirements for heaters, air conditioners or refrigerators in buildings (such as the regulation "GB 4706.13-2014 on Safety on Household and Similar Electrical Appliances" adopted in China in 2016 or the regulation "SI 994-1 on Safety and Performance Requirements of Air Conditioners" adopted in Israel in 2009), mandatory reporting requirements for energy efficiency measures (such as a regulation adopted in the Netherlands in 2019) or guidelines for estimating greenhouse gas emissions (such as a regulation adopted in Australia in 2017 that provides technical guidelines for estimating greenhouse gas emissions in energy installations). This increases the heteroskedasticity (noise) within our coding of regulatory instrument types.

Our country vignettes in SI2 further show that the majority of regulations relate to the buildings and transport sectors, where emission reductions have been relatively low, especially when compared to emission reductions in the energy-supply sector.

We are unable to control for the differing stringency of regulations within our policy sample.

Table S17. Models with policy density by instrument type as independent variable

VARIABLES	(1)<br>Log(CO2/GDP)	(2)<br>Log(CO2/GDP)	(3)<br>Log(CO2/GDP)
Policy density (regulatory instruments)	-0.000359		
	(0.000348)		
Policy density (economic instruments)		-0.00150***	
		(0.000297)	
Policy density (soft instruments)			-0.000771*
			(0.000448)
**CONTROLS**			
Rule of law	-0.0327	-0.0438	-0.0344
	(0.0303)	(0.0299)	(0.0304)
Hodrick-Prescott GDP filter	-0.377	-0.330	-0.377
	(0.252)	(0.248)	(0.251)
GDP per capita log	3.607***	3.668***	3.637***
	(0.290)	(0.287)	(0.291)
GDP per capita log squared	-0.194***	-0.197***	-0.195***
	(0.0154)	(0.0153)	(0.0155)
Imports share to GDP	-0.00389***	-0.00422***	-0.00394***
	(0.000650)	(0.000641)	(0.000645)
Services share to GDP	-0.00835***	-0.00762***	-0.00830***
	(0.00220)	(0.00217)	(0.00219)
Temperature variation	-0.0163**	-0.0176**	-0.0170**
	(0.00791)	(0.00789)	(0.00792)
Constant	-17.28***	-17.53***	-17.46***
	(1.328)	(1.301)	(1.338)
Observations	941	941	941
Years	2000-2022	2000-2022	2000-2022
R-squared	0.967	0.968	0.967
within R-squared	0.2654	0.2842	0.2665
RMSE	0.0923	0.0912	0.0923

Notes: Dependent variable is the log of emission intensity. Robust standard errors are reported in parentheses. *** p<0.01, ** p<0.05, * p<0.1

5.9 Emission intensity by sector

We account for the concentration of policies on higher emitting sectors in our emissions-weighted diversity measure of policy sectoral coverage. We further explore the effect of variation in countries' sectoral emission shares by running additional models for each sector (except energy industry) using sectoral CO_2 emissions normalised by total GDP as the dependent variable, and sectoral policy counts as the independent variable. For each model, we also test sectoral policy stringency (using the sectoral CAPMF index) as an alternative independent variable. We report these sectoral model results in full in Table S18.

This approach is limited in that the GDP normalisation is for the whole economy not the sector (for which sufficient time series data were not available to match our country x time panel). However, all the sectoral model results show negative and significant coefficients for policy density and for the alternative policy stringency variable as expected, and consistent with the main economy-wide results. The one exception is that policy density in the buildings sector model is negative but not significant. As the alternative specification using policy stringency does show a significant coefficient, our interpretation is that this result is due to reduced heterogeneity as some smaller countries have very few buildings sector policies.

Table S18. Models with emission intensity for each sector as dependent variables

	Manufacturing and construction		Transport		Buildings		Electricity and heat production	
	(1)	(2)	(3)	(4)	(5)	(6)	(7)	(8)
**VARIABLES**	**LOG(CO2/GDP)**	**LOG(CO2/GDP)**	**LOG(CO2/GDP)**	**LOG(CO2/GDP)**	**LOG(CO2/GDP)**	**LOG(CO2/GDP)**	**LOG(CO2/GDP)**	**LOG(CO2/GDP)**
Policy density (sector specific)	-0.00693***		-0.00550***		-0.000810		-0.00399***	
	(0.00168)		(0.000468)		(0.000616)		(0.00118)	
Policy stringency (sector specific)		-0.0151***		-0.0146***		-0.0406***		-0.0306**
		(0.00458)		(0.00524)		(0.00681)		(0.0132)
**CONTROLS**								
Rule of law	-0.0716	-0.0656	-0.0704***	-0.0216	-0.0527	-0.0856*	0.0496	0.0175
	(0.0501)	(0.0522)	(0.0244)	(0.0284)	(0.0535)	(0.0519)	(0.0654)	(0.0674)
Hodrick-Prescott GDP filter	0.741**	0.676**	0.460***	0.363*	-0.288	-0.537*	0.670	0.526
	(0.308)	(0.315)	(0.165)	(0.186)	(0.313)	(0.318)	(0.488)	(0.481)
GDP per capita log	4.499***	4.234***	2.284***	2.964***	5.085***	3.805***	6.276***	5.509***
	(0.439)	(0.430)	(0.287)	(0.333)	(0.586)	(0.601)	(0.497)	(0.635)
GDP per capita log squared	-0.243***	-0.230***	-0.115***	-0.151***	-0.287***	-0.218***	-0.324***	-0.288***
	(0.0228)	(0.0222)	(0.0150)	(0.0170)	(0.0297)	(0.0305)	(0.0257)	(0.0330)
Imports share to GDP	-0.00732***	-0.00660***	-0.00274***	-0.00203**	-0.000555	0.000206	-0.0144***	-0.0143***
	(0.00135)	(0.00143)	(0.000820)	(0.000859)	(0.00140)	(0.00138)	(0.00206)	(0.00205)
Services share to GDP	-0.0166***	-0.0154***	0.00147	-0.00484**	-0.0204***	-0.0140***	-0.0130**	-0.0202***
	(0.00299)	(0.00326)	(0.00209)	(0.00228)	(0.00381)	(0.00401)	(0.00589)	(0.00588)
Temperature variation	0.00412	0.00948	-0.00709	-0.00974	-0.0653***	-0.0623***	-0.102***	-0.103***
	(0.0114)	(0.0115)	(0.00650)	(0.00709)	(0.0128)	(0.0129)	(0.0190)	(0.0190)
Observations	919	876	919	876	917	874	919	876
Years	2000-2022	2000-2022	2000-2022	2000-2022	2000-2022	2000-2022	2000-2022	2000-2022
R-squared	0.998	0.998	0.999	0.999	0.998	0.998	0.994	0.994
within R-squared	0.5642	0.5562	0.5841	0.5261	0.5957	0.6082	0.5487	0.5772
RMSE	0.1724	0.1769	0.083	0.0893	0.1836	0.184	0.2528	0.2462

Notes: Dependent variable is the log of emission intensity. Robust standard errors are reported in parentheses. *** $p<0.01$, ** $p<0.05$, * $p<0.1$

5.10 Combinations of individual variables

In our models we introduce additional policy portfolio elements separately alongside policy density. We do not report a full combined model with all our independent variables together due to collinearity problems from overspecification that leads to: unstable coefficient estimates and difficulty in determining the individual effects of each variable; loss of efficiency, reducing the precision of the estimated coefficients and increasing standard errors; and overfitting.

However, we do run further tests to see how complete a model we can specify without compromising model quality and interpretability. In Table S19, we report full combined models with our main policy density variable, one of the four policy portfolio variables (diversity of instrument type and sectoral coverage), and all five dummy variables (for absolute and relative long-term), for energy and/or climate ministries, and for IEA and EU-EFTA membership. However, these models do not include the interaction terms between dummies and policy density.

All coefficient signs and magnitudes are in line with those of the more parsimonious models reported in the main text. Across all models, the coefficient for total policy density remains strongly significant. The same holds for each of the policy portfolio variables that continue to perform well in their respective combined model. Significance of the energy and/or climate ministry dummy drops away, as does the EU-EFTA membership (which is highly correlated with IEA membership). However, overall, these combined models give us further confidence in the results reported in main text despite their overspecification.

Table S19. Combined models incorporating combinations of independent variables

VARIABLES	(1)<br>LOG(CO2/GDP)	(2)<br>LOG(CO2/GDP)	(3)<br>LOG (CO2/GDP)	(4)<br>LOG(CO2/GDP)
Policy density	-0.000799***	-0.000832***	-0.000784***	-0.000714***
	(0.000177)	(0.000171)	(0.000169)	(0.000173)
HHI 6 instrument categories	-0.110***			
	(0.0415)			
HHI 3 instrument types		-0.0871**		
		(0.0424)		
HHI sectoral emissions weighted			-0.306***	
			(0.106)	
HHI sectoral				-0.142***
				(0.0498)
Absolute with 1990 baseline emission targets (dummy)	-0.0610***	-0.0611***	-0.0701***	-0.0670***
	(0.0164)	(0.0167)	(0.0161)	(0.0165)
Relative emissions targets (dummy)	-0.0318	-0.0304	-0.0303	-0.0289
	(0.0232)	(0.0227)	(0.0231)	(0.0225)
Energy and/or climate ministry (dummy)	-0.0108	-0.00949	-0.00857	-0.00544
	(0.0103)	(0.0102)	(0.0101)	(0.0101)
IEA (dummy)	-0.0941***	-0.0900**	-0.103***	-0.0991***
	(0.0353)	(0.0355)	(0.0365)	(0.0366)
EU-EFTA (dummy)	-0.00994	-0.0133	-0.0100	-0.0174
	(0.0249)	(0.0249)	(0.0247)	(0.0256)
**CONTROLS**				
Rule of law	-0.0577**	-0.0582**	-0.0526*	-0.0595**
	(0.0278)	(0.0282)	(0.0285)	(0.0279)
Hodrick-Prescott GDP filter	-0.436*	-0.461*	-0.402*	-0.349
	(0.239)	(0.240)	(0.239)	(0.240)
GDP per capita log	3.768***	3.802***	3.880***	3.881***
	(0.349)	(0.349)	(0.347)	(0.338)
GDP per capita log squared	-0.198***	-0.200***	-0.204***	-0.204***
	(0.0180)	(0.0181)	(0.0180)	(0.0175)
Imports share to GDP	-0.00360***	-0.00361***	-0.00344***	-0.00345***
	(0.000659)	(0.000656)	(0.000648)	(0.000648)
Services share to GDP	-0.00658***	-0.00675***	-0.00683***	-0.00677***
	(0.00223)	(0.00226)	(0.00227)	(0.00223)
Temperature variation	-0.0234***	-0.0231***	-0.0227***	-0.0209***
	(0.00739)	(0.00741)	(0.00733)	(0.00731)
Constant	-18.40***	-18.58***	-18.99***	-18.92***
	(1.668)	(1.661)	(1.654)	(1.611)
Observations	919	919	923	923
Years	2000-2022	2000-2022	2000-2022	2000-2022
R-squared	0.970	0.970	0.970	0.970
within R-squared	0.3045	0.3019	0.2967	0.2994

RMSE	0.0882	0.0883	0.0884	0.0882

Notes: Dependent variable is the log of emission intensity. Robust standard errors are reported in parentheses. *** p<0.01, ** p<0.05, * p<0.1

5.11 Comparison with policy stringency models

We use the OECD's Climate Actions and Policies Measurement Framework (CAPMF) as an alternative independent variable to policy density for robustness testing of our main model, despite the mismatch of policy and country samples (no policy stringency data for Brazil and the US). We take the mean of the sectoral policy stringency indices from CAPMF weighted by each sector's share of total emissions. This is similar to our procedure for constructing emission-weighted diversity indices in our testing of policy sectoral coverage. We show the full results in Table S20.

Table S20. Model with policy stringency as independent variable

VARIABLES	(1)<br>Model (1) in main text<br>Log(CO2/GDP)	(2)<br>Policy stringency<br>Log(CO2/GDP)
Policy density	-0.000554***	
	(0.000169)	
Policy stringency index		-0.130***
		(0.0395)
**CONTROLS**		
Rule of law	-0.0374	-0.0549*
	(0.0301)	(0.0310)
Hodrick-Prescott GDP filter	-0.354	-0.449*
	(0.249)	(0.247)
GDP per capita log	3.735***	3.427***
	(0.288)	(0.301)
GDP per capita log squared	-0.200***	-0.183***
	(0.0153)	(0.0161)
Imports share to GDP	-0.00410***	-0.00364***
	(0.000647)	(0.000622)
Services share to GDP	-0.00782***	-0.00910***
	(0.00220)	(0.00218)
Temperature variation	-0.0176**	-0.0141*
	(0.00792)	(0.00803)
Constant	-17.94***	-16.42***
	(1.318)	(1.364)
Observations	941	898
Years	2000-2022	2000-2022
R-squared	0.968	0.969
within R-squared	0.2728	0.2801
RMSE	0.0919	0.09

Notes: Dependent variable is the log of emission intensity. Robust standard errors are reported in parentheses. *** p<0.01, ** p<0.05, * p<0.1

6 Endogeneity & causality

Generally we frame our analysis as showing associations between policy portfolio variables and emission intensity reductions, with literature supporting the inference that policies are antecedent. However we recognise that the term ‘attribution’ - the field of climate policy science to which our study contributes - does also imply causation. In the applied economics and political science literature, causal claims are only possible with Difference-in-Differences (DiD) methods [41]. In our work, we follow precedent [6] in positioning our approach as an attribution study.

We have multiple lines of reasoning to support our attribution of emission intensity reductions to climate policy portfolios rather than vice versa. These are: (1) lagged models, (2) endogeneity tests, (3) Granger non-causality tests, (4) fixed effects, (5) weak correlations with covariates, (6) supporting literature and policy vignettes, (7) our choice of econometric model. We discuss each in turn.

6.1 Lagged models

We examine the lagged effect of policy density on emission intensity. A significant lagged effect indicates that an increase in the stock of policies drives emission intensity reduction, rather than the reverse. We report a 3 year lagged model in Table S21 showing a negative and significant coefficient for the policy density variable in line with expectations. We get broadly similar results for 1, 2, and 4 year lags.

Table S21. Base model with lagged effect of policy density

	model (1) in main text	Lagged model
	(1)	(2)
**VARIABLES**	**Log(CO2/GDP)**	**Log(CO2/GDP)**
Policy density	-0.000554***	
	(0.000169)	
Policy density - Lag 3 years		-0.000548***
		(0.000182)
**CONTROLS**		
Rule of law	-0.0374	-0.0312
	(0.0301)	(0.0327)
Hodrick-Prescott GDP filter	-0.354	-0.402
	(0.249)	(0.253)
GDP per capita log	3.735***	3.860***
	(0.288)	(0.332)
GDP per capita log squared	-0.200***	-0.204***
	(0.0153)	(0.0173)
Imports share to GDP	-0.00410***	-0.00362***
	(0.000647)	(0.000680)
Services share to GDP	-0.00782***	-0.00801***
	(0.00220)	(0.00241)
Temperature variation	-0.0176**	-0.0216***
	(0.00792)	(0.00813)
Constant	-17.94***	-18.82***
	(1.318)	(1.562)
Observations	941	855
Years	2000-2022	2000-2022
R-squared	0.968	0.969
within R-squared	0.2728	0.2459
RMSE	0.0919	0.0903

Notes: Dependent variable is the log of emission intensity. Robust standard errors are reported in parentheses. *** p<0.01, ** p<0.05, * p<0.1

However, we recognise that policy density does not change much over some time periods in some countries, so the lagged policy density variable could absorb the effects of other variables. In a further model reported in Table S13 we include a 1 year lag on all controls as

well as the policy density variable. Below we show that our controls are only weakly correlated with our policy density variable.

6.2 Endogeneity tests

We ran standard endogeneity tests used in applied econometrics, specifically the Durbin-Wu-Hausman test. Full results are included in Table S21.

Table S22. Durbin-Wu-Hausman endogeneity test for policy density

VARIABLES	Model E1<br>(1)<br>Log(CO2/GDP)	Model E2<br>(2)<br>Policy density	Model E3<br>(3)<br>LOG(CO2/GDP)
Policy density	-0.000554***		-0.000436
	(0.000169)		(0.000569)
Policy stringency		15.57***	
		(1.385)	
Model E2 residuals			1.11e-05
			(0.000617)
**CONTROLS**			
Rule of law	-0.0374	-8.407*	-0.0490
	(0.0301)	(4.521)	(0.0309)
Hodrick-Prescott GDP filter	-0.354	31.41	-0.456*
	(0.249)	(36.77)	(0.251)
GDP per capita log	3.735***	423.9***	3.593***
	(0.288)	(52.95)	(0.385)
GDP per capita log squared	-0.200***	-20.36***	-0.192***
	(0.0153)	(2.754)	(0.0199)
Imports share to GDP	-0.00410***	-0.444***	-0.00393***
	(0.000647)	(0.119)	(0.000696)
Services share to GDP	-0.00782***	1.268***	-0.00870***
	(0.00220)	(0.414)	(0.00231)
Temperature variation	-0.0176**	-3.016**	-0.0154*
	(0.00792)	(1.195)	(0.00828)
Constant	-17.94***	-2,252***	-17.30***
	(1.318)	(261.7)	(1.837)
Observations	941	898	898
Years	2000-2022	2000-2022	2000-2022
R-squared	0.968	0.861	0.969
F test (for Model B2 residuals)			0
F test P value			0.9857
Within R-squared	0.2728	0.3113	0.273
RMSE	0.0919	15.3462	0.0905

Notes: Dependent variable is the log of emission intensity. Robust standard errors are reported in parentheses. *** p<0.01, ** p<0.05, * p<0.1

As an instrument for policy density, we use the sectoral stringency index (OECD CAPMF) which we know from literature controls for a similar effect to policy density on emission intensity, also indicated by the high correlation between the two variables (71%) in our panel.

We start with model E1 specified as:

$$y_i = \beta_0 + \beta_1 x_1 + \beta_2 Z + u_i \qquad \text{E1}$$

in which y_i is the dependent variable, x_1 is the variable that we want to test for endogeneity, and Z is a vector of exogenous variables. Following Wooldridge (2003, p. 483-484), the next step involves model E2 in which we regress the variable for which we are uncertain whether it is an endogenous predictor (i.e., x_1), with an additional exogenous variable i.e., the instrument variable x_2, and the vector of exogenous variables Z incorporated in model E1:

$$x_1 = \pi_0 + \pi_1 x_2 + \beta_2 Z + v_i \qquad \text{E2}$$

The second step involves regressing model E3 in which we effectively estimate the original model E1, while also incorporating the residuals v_i from model E2:

$$y_\mathrm{i} = \beta_0 + \beta_1 x_1 + \beta_2 Z + \delta v_i + u_i \qquad \text{E3}$$

We then test the null hypothesis H_0 that the coefficient $\delta = 0$, which essentially means that x_1 is not endogenous as the two error terms are not correlated. This in indeed the case. (The alternative hypothesis H_0 under which $\delta \neq 0$ indicates that x_1 is endogenous).

In our case we start with the base model E1 for policy density then run model E2 in which emission intensity is replaced by policy density as the dependent variable, while simultaneously introducing the OECD CAPMF sectoral stringency index as an independent variable. We save the residuals from model E2 and add them in to the base model E3 as an additional regression. We observe that the coefficient for the model E2 residuals does not reject the null hypothesis that the coefficient δ=0. As a final test, we perform an f-test on the coefficient for the model E2 residuals that fails to reject the null. This further shows that the specific regressor is not endogenous, giving us further evidence to reject the reverse causal explanation that emission intensity results in higher policy density.

6.3 Granger non-causality tests

As an additional sensitivity analysis of the relationship between total policy density and emission intensity, we specify Granger non-causality following Dumitrescu and Hurlin [42] as:

$$Y_{it} = \alpha_i + \sum_{k=1}^{K} \gamma_{ik} Y_{i,t-k} + \sum_{k=1}^{K} \beta_{ik} X_{i,t-k} + \varepsilon_{it} \qquad \text{C1}$$

in which Y_{it} is our dependent variable, i.e. natural log of emission intensity, and X_{it} is independent variable, i.e. total policy density, for country i and year t. We assume the lag order k to be identical for all countries i, and the panel to be balanced.

The Dumitrescu-Hurlin (2012) panel Granger non-causality test, represented in Equation C1, considers the heterogeneity of causal relationships. The key idea is that if past values of total policy density ($X_{i,t-k}$) are significant predictors of the current value of emission intensity reduction (Y_{it}) when past values of emission intensity ($Y_{i,t-k}$) are incorporated in model C1, then higher total policy density (X_{it}) has causal influence on emission intensity reduction

(Y_{it}). The null hypothesis (H_0) assumes no causal relationships between total policy density and emission intensity reduction for any of the cross-sectional units (H_0: $\beta_i = 0, (i = 1, \ldots, N)$). The test computes an average Wald statistic, averaged across all cross-sectional units (countries i), that can be used to test the alternative hypothesis that X_{it} Granger-causes Y_{it} for at least a subset of countries, if not for all (H_1: $\beta_i = 0, (i = 1, \ldots N_1);\ \beta_i \neq 0, (i = N_1 + 1, N_1 + 2, \ldots, N)$).

Results of our testing are summarised in Table S22. We first perform the test reported in Equation C1, which, by default, includes a lag operator of k=1. Similar to Section 6.1, we re-perform the test using a lag operator of order k =3 for both the autoregressive parameter γ_{ik} and the regression coefficients slopes β_{ik}. This allows us to test whether a three-year lagged effect of total policy density Granger-causes emission intensity reduction at current year t. The null hypothesis of Granger non-causality is rejected at 1% statistical significance level, a result that holds when we use a lag operator of order 3. We get similar results for lag operators equal to 2 and 4. So, we find robust statistical evidence of causal relationship between total policy density and emission intensity reduction for at least a subset of countries.

Table S23. Dumitrescu-Hurlin (2012) test on whether policy density Granger-causes emission intensity reduction.

	Lag 1			Lag 3		
	W-stat	Zbar-stat	P-value	W-stat	Zbar-stat	P-value
Policy density	6.5810	25.8779	0.0000	10.3218	19.6010	0.0000

Notes: Null hypothesis that total policy density does not Granger-cause reduction in the natural logarithm of emissions intensity. Rejection of the null indicates there is Granger causality. Lag 1 indicates the default option of a lag operator k of order 1 in model C1, while Lag 3 the option of a lag operator k of order 3.

We note that although the Dumitrescu-Hurlin test, like most statistical tests of causality, is useful for detecting potential temporal causal relationships between independent and dependent variables, it has limitations in establishing reverse causality for which arguments based on prior hypotheses and causal reasoning are important.

6.4 Fixed effects

Empirically, policy density is affected by differing policy traditions across countries, which is captured in our models by the country fixed effects. These include time-invariant differences in socioeconomic context, political culture, institutional quality, and resource endowment between countries. Institutions are typically slow-moving and path-dependent, but over the 2000–2022 period we have observed meaningful institutional shifts in several countries particularly within the BRIICS including through crises, regime changes, democratisation, autocratization, and reforms. As these institutional shifts may impact the effectiveness with which climate policy density affects emission intensities (e.g., through changes to policy credibility, monitoring, and enforcement, we include the World Bank’s rule of law index as a control in our models.

Policy density also increases in all countries over time (shown in Fig S3A-C) due to increasing issue salience and prioritisation as well as other secular trends (e.g. economic development and strengthening policy capacity). This is captured in our models by year fixed effects. Both the country and year fixed effects help avoid our findings being the result of spurious correlations.

6.5 Weak correlation between policy density & control variables

There is generally low or very low correlation between policy density and the control variables used in our models over the period 2000-2022. The highest correlation is 22.8% for services share of GDP; all others are below 20% (temperature variation 19.5%, imports share of GDP -16.6%, GDPpercap log 14.0%, rule of law 0.08%, Hodrick-Prescott GDP filter -0.01%). These are all substantially lower than the correlation with the OECD CAMPF sectoral stringency index (71%), which we use as an instrument to test for endogeneity. In general we find that policy stocks are not correlated with general economic development or other variables and dynamics.

6.6 Supporting literature and policy vignettes

Consistent with the literature, we are confident that policy density is plausibly exogenous, and that our analysis goes beyond a descriptive assessment. All the climate policy portfolio elements we include in our models are designed to reduce emissions in line with policy priorities post-Kyoto Protocol (1997) in many OECD countries, and post-Paris Agreement (2015) in all OECD+BRIICS countries. Our policy vignettes (see SI2) indicate that the implementation of new policies increasing the total stock is antecedent to observed emission intensity reductions, rather than the other way round.

We align with the statistical attribution framework for 'global' policy impacts established by Eskander and Fankhauser [6] in the climate policy attribution literature, extending their testing of policy density (cumulative policy stocks) to additionally assess the effect of policy stringency (see also Nachtigall, et al. [43]), policy instrument types, policy sectoral coverage, targets and policy-relevant organisations.

Here, we summarise relevant policy attribution literature to show how both our attribution approach and our estimated emission reductions attributable to climate policy portfolios are consistent within this literature.

In Chapter 14 of the IPCC's WG3 Sixth Assessment Report, Babiker, et al. [44] define policy attribution as: "the extent to which emission-relevant outcomes (including the emission intensity of GDP) charted for countries as well as sectors and technologies may be reasonably attributed to policies implemented prior to the observed changes."

Most ex-post policy impact assessments are for specific policy instruments in particular contexts [45]. Very few studies assess collective or 'global' impacts of policy stocks comprising multiple instrument types. Attribution methodologies used to identify the effect of mitigation policies controlling for confounding factors like socioeconomic conditions, fossil fuel prices, and trade-related policies, include "statistical attribution methodologies, including experimental and quasi-experimental design, instrumental variable approaches, and simple correlational methods" [44].

In their review of both 'global' and instrument-specific attribution literature, Hoppe, et al. [45] estimate a plausible range of emission reduction of 2-7 GtCO_2e/yr compared to a no-policy counterfactual, equivalent to 4-5% of total GHG emissions in 2020. Evidence from multiple studies span an attribution range of 1.3-5.9 GtCO_2/yr for different country samples and time periods:

- Eskander and Fankhauser [6] attribute reductions of 5.9 GtCO_2/yr (in 2016), or 38 GtCO2-eq. cumulatively, to climate laws (1999-2016, 133 countries);

- Maamoun [46] attribute reductions of 1.3 $GtCO_2$/yr (in 2012), equivalent to -7%/yr, to climate policies pursuant to Kyoto Protocol emission-reduction targets (2005-2012, 39 countries with in UNFCCC Annex B);
- Babiker, et al. [44] attribute reductions of 1.8-3 $GtCO_2$/yr to multiple policy instruments including energy-efficiency programmes and renewables diffusion (unspecified time horizon & country sample);
- Grubb, et al. [47] attribute reductions of 4-5 $GtCO_2$/yr (in 2019) to the cumulative impact of policies (2010-2019, global);
- UNFCCC [48] attribute projected reductions of ~3.8 $GtCO_2$/yr to the 38% of >2500 implemented policies and measures for which estimates are available (36 countries in UNFCCC Annex 1);
- Hoppe, et al. [45] draw on IRENA data to attribute emission reductions of 1.3-2.5 $GtCO_2$/yr to renewable energy policy.

Major attribution studies since the IPCC assessment, including those for specific policy instruments (carbon pricing) or targeted sectors (renewable energy), are:

- Stechemesser, et al. [41] attribute reductions of 0.6-1.8 $GtCO_2$ from discontinuous breaks in emission trends to 69 sets of policies in combination (2000-2020, 41 countries);
- Nachtigall, et al. [43] attribute emission reductions of 28% to climate policies compared to a no-policy counterfactual, emphasising the increasing stringency of policy portfolios over time (2000-2021, 48 countries);
- Eskander and Fankhauser [31] attribute emission intensity reductions over the long-term (>3 years) of 0.84–2.78% (territorial or production-based) or 0.91–2.61% (consumption-based) to each new climate law introduced (1996–2018, 111 countries);
- Döbbeling-Hildebrandt, et al. [49] meta-analyse 80 *ex post* evaluation studies of 21 carbon pricing schemes worldwide to generalise a de-biased effect of this specific policy instrument to the order of -4% to -15% emission reductions.

Most notably, Stechemesser, et al. [41] advanced ‘global’ attribution methodology through their use of a difference-in-differences (DiD) approach to evaluate the effect of specific policies or combinations of policies in 41 countries over the period 2000-2020. They focus on a specific set of 69 structural breaks (discontinuities) in emission trends. They attribute total emission reductions of 0.6-1.8 $GtCO_2$ to identifiable combinations of policies implemented in the 2 years prior to the structural break. that can be attributed to a set of 69 policy-attributed structural breaks in emission trends (41 countries, 2000-2020).

The country samples and time periods in Stechemesser, et al. [41] and our study are very similar, but there are important differences that help explain why their estimated emission reductions attributable to policy are an order of magnitude lower.

First, their aim is to assess policy impacts on major near-term reductions in emissions; their dependent variable is structural breaks - statistically identifiable discontinuities - in sectoral emission trends. Our aim is to assess policy portfolios’ cumulatively incremental impact on emission reductions; our dependent variable is continuous economy-wide emission trends. Discontinuities will be subsumed within these aggregate trends.

Second, their method uses difference-in-differences (DiD) testing to attribute emission discontinuities to specific combinations of policy instruments introduced or tightened in the preceding 2 years in the corresponding sector. The DiD approach allows them to draw robust causal inferences on attribution. Our method uses two-way fixed effect models to identify associations between cumulative policy portfolio elements and emission reductions. Our method does not allow causal inference, so we provide multiple other lines of evidence

supporting our attribution claim. The cumulative focus of our policy portfolio attribution allows us to test and demonstrate the interacting effect of framework targets and governmental organisations over the longer-term: an additional reason why our attributed emission reductions are larger.

6.7 Our choice of econometric model

Our choice of the two-way fixed effects (TWFE) estimator is grounded in evidence from the applied econometrics literature that emphasises its capability in establishing causality. Influential studies in applied economics [21] and in political science [50] underscore the effectiveness of fixed effects models for identifying causal relationships in panel data. Comprehensive overviews of the econometric properties of the TWFE estimator elaborate on its strengths and limitations in the context of causal inference with panel datasets [22,51].

We make these arguments to support our decision to use the TWFE estimator, incorporating cross-sectional and time dummies, given our study aim of assessing the overall impact of policy density on emission intensity. With all the controls in our model, we are confident the estimated effect on emission intensity is clearly attributable to changes in policy density.

As noted, the main alternative method which allows for robust causal claims is the Difference-in-Difference (DiD) model used by Stechemesser, et al. [41] to estimate the emission impact of specific policies or policy combinations. Given we examine the effect of accumulating policy stocks on emissions intensity, a similar approach focusing on distinct individual policies would not work. Directly comparing individual policies across countries would invoke concerns over robustness, given the fundamental differences in the policies' design, which are tailored to each country's specific characteristics and needs. Such comparisons would fall under the umbrella term of 'bad comparison' in which dissimilar policies are evaluated against each other. Baker, et al. [52] argue that when staggered timing of treatment effects (i.e. policies introduced at different dates) is combined with treatment effect heterogeneity (i.e. varying types of policies across different countries), DiD estimates become biased and consequently misleading. This is particularly true in the case of dynamic treatment effects [53]. For these reasons we do not consider DiD methods appropriate for our study of national climate policy portfolios.

7 Supplementary References

1 Tosun, J. Investigating Ministry Names for Comparative Policy Analysis: Lessons from Energy Governance. *Journal of Comparative Policy Analysis: Research and Practice* **20**, 324-335 (2018). https://doi.org:10.1080/13876988.2018.1467430

2 Evans, N. & Duwe, M. Climate governance systems in Europe: the role of national advisory bodies. (Ecologic Institute & IDDRI, Berlin & Paris, 2021).

3 Averchenkova, A., Fankhauser, S. & Finnegan, J. J. The influence of climate change advisory bodies on political debates: evidence from the UK Committee on Climate Change. *Climate Policy* **21**, 1218-1233 (2021). https://doi.org:10.1080/14693062.2021.1878008

4 Weaver, S., Lötjönen, S. & Ollikainen, M. Overview of national climate change advisory councils. (University of Helsinki, Helsinki, Finland, 2019).

5 Tosun, J. & Mišić, M. Post-Communist Countries' Participation in Global Forums on Climate Action. *Problems of Post-Communism* **69**, 380-395 (2022). https://doi.org:10.1080/10758216.2021.1994423

6 Eskander, S. M. S. U. & Fankhauser, S. Reduction in greenhouse gas emissions from national climate legislation. *Nature Climate Change* **10**, 750-756 (2020). https://doi.org:10.1038/s41558-020-0831-z

7 Viola, E. & Basso, L. Brazilian Energy-Climate Policy and Politics towards Low Carbon Development. *Global Society* **29**, 427-446 (2015). https://doi.org:10.1080/13600826.2015.1028904

8 Pischke, E. C., Solomon, B. D. & Wellstead, A. M. A historical analysis of US climate change policy in the Pan-American context. *Journal of Environmental Studies and Sciences* **8**, 225-232 (2018). https://doi.org:10.1007/s13412-018-0476-7

9 Valdmaa, K. Development of the environmental taxes and charges system in Estonia: international convergence mechanisms and local factors. *Policy Studies* **35**, 339-356 (2014). https://doi.org:10.1080/01442872.2013.875152

10 Dudley, H., Jordan, A. & Lorenzoni, I. Advising national climate policy makers: A longitudinal analysis of the UK Climate Change Committee. *Global Environmental Change* **76**, 102589 (2022). https://doi.org:https://doi.org/10.1016/j.gloenvcha.2022.102589

11 Tosun, J. & Rinscheid, A. What drives engagement in the Clean Energy Ministerial? An assessment of domestic-level factors. *Journal of European Public Policy* **30**, 469-487 (2023). https://doi.org:10.1080/13501763.2021.2014936

12 Moore, B., Oberthür, S., Duwe, M., Kögel, N., Evans, N., Homeyer, I. v., Kulovesi, K., Kampman, B., Hilke, A., Mähönen, M. & Varis, K. Transformative procedural climate governance: Mechanisms, functions, and assessment criteria. . (Vrije Universiteit Brussel, Brussels, Belgium, 2023).

13 IEA. *Policies & measures database*. (International Energy Agency, 2023).

14 Patt, A., Rajamani, L., Bhandari, P., Boncheva, A. I., Caparrós, A., Djemouai, K., Kubota, I., Peel, J., Sari, A. P., Sprinz, D. F. & Wettestad, J. in *Climate Change 2022: Mitigation of Climate Change. Working Group III contribution to the Sixth Assessment*

Report of the Intergovernmental Panel on Climate Change (IPCC) (eds P. R. Shukla, J. Skea, R. Slade, A. Al Khourdajie, R. van Diemen, D. McCollum, M. Pathak, S. Some, P. Vyas, R. Fradera, M. Belkacemi, A. Hasija, G. Lisboa, S. Luz, & J. Malley) Ch. 14, 1451-1546 (Intergovernmental Panel on Climate Change (IPCC), 2022).

15 Climate Change Laws of the World. (Grantham Research Institute on Climate Change and the Environment,, London, UK, 2024).

16 Dubash, N. K., Mitchell, C., Boasson, E. L., Borbor-Cordova, M. J., Fifita, S., Haites, E., Jaccard, M., Jotzo, F., Naidoo, S., Romero-Lankao, P., Shlapak, M., Shen, W. & Wu, L. in *Climate Change 2022: Mitigation of Climate Change. Working Group III contribution to the Sixth Assessment Report of the Intergovernmental Panel on Climate Change (IPCC)* (eds P. R. Shukla, J. Skea, R. Slade, A. Al Khourdajie, R. van Diemen, D. McCollum, M. Pathak, S. Some, P. Vyas, R. Fradera, M. Belkacemi, A. Hasija, G. Lisboa, S. Luz, & J. Malley) Ch. 13, 1355-1450 (Intergovernmental Panel on Climate Change (IPCC), 2022).

17 Schaub, S., Tosun, J. & Jordan, A. Climate Action through Policy Expansion and/or Dismantling: Country-Comparative Insights. *Journal of Comparative Policy Analysis: Research and Practice* (2024). https://doi.org:https://doi.org/10.1080/13876988.2024.2369640

18 Schaub, S., Tosun, J., Jordan, A. & Enguer, J. Climate Policy Ambition: Exploring A Policy Density Perspective. *Politics and Governance* **10**, 13 (2022). https://doi.org:10.17645/pag.v10i3.5347

19 IEA. *Net Zero by 2050: A Roadmap for the Global Electricity Sector*. (International Energy Agency, 2021).

20 Tosun, J., Heinz-Fischer, C. & Luo, R. Who takes the lead? A disaggregate analysis of the EU's engagement in the Clean Energy Ministerial and Mission Innovation. *Journal of Cleaner Production* **382**, 135240 (2023). https://doi.org:https://doi.org/10.1016/j.jclepro.2022.135240

21 Angrist, J. D. & Pischke, J.-S. The Credibility Revolution in Empirical Economics: How Better Research Design Is Taking the Con out of Econometrics. *Journal of Economic Perspectives* **24**, 3–30 (2010). https://doi.org:10.1257/jep.24.2.3

22 Wooldridge, J. M. Two-Way Fixed Effects, the Two-Way Mundlak Regression, and Difference-in-Differences Estimators. *Available at SSRN: https://ssrn.com/abstract=3906345* (2021).

23 Cinelli, C. & Hazlett, C. Making Sense of Sensitivity: Extending Omitted Variable Bias. *Journal of the Royal Statistical Society Series B: Statistical Methodology* **82**, 39-67 (2019). https://doi.org:10.1111/rssb.12348

24 Celli, V. Causal mediation analysis in economics: Objectives, assumptions, models. *Journal of Economic Surveys* **36**, 214-234 (2022). https://doi.org:https://doi.org/10.1111/joes.12452

25 Cinelli, C., Forney, A. & Pearl, J. A Crash Course in Good and Bad Controls. *Sociological Methods & Research* **53**, 1071-1104 (2024). https://doi.org:10.1177/00491241221099552

26 Cunningham, S. *Causal inference: The mixtape*. (Yale university press, 2021).

27 Pearl, J. *Causality*. 2 edn, (Cambridge University Press, 2009).

28 Herrendorf, B., Rogerson, R. & Valentinyi, Á. in *Handbook of Economic Growth* Vol. 2 (eds Philippe Aghion & Steven N. Durlauf) 855-941 (Elsevier, 2014).

29 Rodrik, D. Institutions for high-quality growth: What they are and how to acquire them. *Studies in Comparative International Development* **35**, 3-31 (2000). https://doi.org:10.1007/BF02699764

30 Rodrik, D. What Do Trade Agreements Really Do? *Journal of Economic Perspectives* **32**, 73–90 (2018). https://doi.org:10.1257/jep.32.2.73

31 Eskander, S. M. S. U. & Fankhauser, S. The Impact of Climate Legislation on Trade-Related Carbon Emissions 1996–2018. *Environmental and Resource Economics* **85**, 167-194 (2023). https://doi.org:10.1007/s10640-023-00762-w

32 Jahn, D. The stringency and potential impact of climate laws and policies in the European Union and the 21OECD countries. *npj Climate Action* **3**, 90 (2024). https://doi.org:10.1038/s44168-024-00175-5

33 Versteeg, M. & Ginsburg, T. Measuring the Rule of Law: A Comparison of Indicators. *Law & Social Inquiry* **42**, 100-137 (2017). https://doi.org:10.1111/lsi.12175

34 Best, R., Burke, P. J. & Jotzo, F. Carbon Pricing Efficacy: Cross-Country Evidence. *Environmental and Resource Economics* **77**, 69-94 (2020). https://doi.org:10.1007/s10640-020-00436-x

35 Acemoglu, D. & Robinson, J. A. The Political Economy of the Kuznets Curve. *Review of Development Economics* **6**, 183-203 (2002). https://doi.org:https://doi.org/10.1111/1467-9361.00149

36 Mummolo, J. & Peterson, E. Improving the Interpretation of Fixed Effects Regression Results. *Political Science Research and Methods* **6**, 829-835 (2018). https://doi.org:10.1017/psrm.2017.44

37 Nachtigall, D., Lutz, L., Rodríguez, M. C., Haščič, I. & Pizarro, R. The climate actions and policies measurement framework: A structured and harmonised climate policy database to monitor countries' mitigation action., (OECD, Paris, France, 2022).

38 AAAS. Beyond Technology: Strengthening Energy Policy through Social Science. (American Academy of Arts & Sciences (AAAS), Cambridge, MA, 2011).

39 Attaran, M. Industrial diversity and economic performance in U.S. areas. *The Annals of Regional Science* **20**, 44-54 (1986). https://doi.org:10.1007/BF01287240

40 Le Quéré, C., Peters, G. P., Friedlingstein, P., Andrew, R. M., Canadell, J. G., Davis, S. J., Jackson, R. B. & Jones, M. W. Fossil CO2 emissions in the post-COVID-19 era. *Nature Climate Change* **11**, 197-199 (2021). https://doi.org:10.1038/s41558-021-01001-0

41 Stechemesser, A., Koch, N., Mark, E., Dilger, E., Klösel, P., Menicacci, L., Nachtigall, D., Pretis, F., Ritter, N., Schwarz, M., Vossen, H. & Wenzel, A. Climate policies that

achieved major emission reductions: Global evidence from two decades. *Science* **385**, 884-892 (2024). https://doi.org:doi:10.1126/science.adl6547

42 Dumitrescu, E.-I. & Hurlin, C. Testing for Granger non-causality in heterogeneous panels. *Economic Modelling* **29**, 1450-1460 (2012). https://doi.org:https://doi.org/10.1016/j.econmod.2012.02.014

43 Nachtigall, D., Lutz, L., Cárdenas Rodríguez, M., D'Arcangelo, F. M., Haščič, I., Kruse, T. & Pizarro, R. The Climate Actions and Policies Measurement Framework: A Database to Monitor and Assess Countries' Mitigation Action. *Environmental and Resource Economics* **87**, 191-217 (2024). https://doi.org:10.1007/s10640-023-00821-2

44 Babiker, M., Bertoldi, P., Bataille, C., Creutzig, F., Dubash, N. K., Grubb, M., Haites, E., Hinder, B., Hoppe, J., Kim, Y., Nemet, G. F., Patt, A., Saheb, Y. & Slade, R. in *Climate Change 2022: Mitigation of Climate Change. Working Group III contribution to the Sixth Assessment Report of the Intergovernmental Panel on Climate Change (IPCC)* (eds P. R. Shukla, J. Skea, R. Slade, A. Al Khourdajie, R. van Diemen, D. McCollum, M. Pathak, S. Some, P. Vyas, R. Fradera, M. Belkacemi, A. Hasija, G. Lisboa, S. Luz, & J. Malley) Ch. 14, 1479-1481 (Intergovernmental Panel on Climate Change (IPCC), 2022).

45 Hoppe, J., Hinder, B., Rafaty, R., Patt, A. & Grubb, M. Three Decades of Climate Mitigation Policy: What Has It Delivered? *Annual Review of Environment and Resources* **48**, 615-650 (2023). https://doi.org:10.1146/annurev-environ-112321-103821

46 Maamoun, N. The Kyoto protocol: Empirical evidence of a hidden success. *Journal of Environmental Economics and Management* **95**, 227-256 (2019). https://doi.org:https://doi.org/10.1016/j.jeem.2019.04.001

47 Grubb, M., Okereke, C., Arima, J., Bosetti, V., Chen, Y., Edmonds, J., Gupta, S., Köberle, A., Kverndokk, S., Malik, A. & Sulistiawati, L. in *Climate Change 2022: Mitigation of Climate Change. Working Group III contribution to the Sixth Assessment Report of the Intergovernmental Panel on Climate Change (IPCC)* (eds Priyadarshi R Shukla, Jim Skea, Raphael Slade, A Al Khourdajie, R Van Diemen, D McCollum, M Pathak, S Some, P Vyas, R Fradera, M Belkacemi, A Hasija, G Lisboa, S Luz, & J Malley) Ch. Chapter 1, (Intergovernmental Panel on Climate Change (IPCC), 2022).

48 UNFCCC. Subsidiary Body for Implementation, Compilation and Synthesis of fourth biennial reports of Parties included in Annex I to the Convention., (UN Framework Convention on Climate Change, Bonn, Germany, 2020).

49 Döbbeling-Hildebrandt, N., Miersch, K., Khanna, T. M., Bachelet, M., Bruns, S. B., Callaghan, M., Edenhofer, O., Flachsland, C., Forster, P. M., Kalkuhl, M., Koch, N., Lamb, W. F., Ohlendorf, N., Steckel, J. C. & Minx, J. C. Systematic review and meta-analysis of ex-post evaluations on the effectiveness of carbon pricing. *Nature Communications* **15**, 4147 (2024). https://doi.org:10.1038/s41467-024-48512-w

50 Imai, K. & Kim, I. S. When Should We Use Unit Fixed Effects Regression Models for Causal Inference with Longitudinal Data? *American Journal of Political Science* **63**, 467-490 (2019). https://doi.org:https://doi.org/10.1111/ajps.12417

51 Millimet, D. & Bellemare, M. F. Fixed Effects and Causal Inference. *IZA Discussion Paper No. 16202* (2021).

52 Baker, A. C., Larcker, D. F. & Wang, C. C. Y. How much should we trust staggered difference-in-differences estimates? *Journal of Financial Economics* **144**, 370-395 (2022). https://doi.org:https://doi.org/10.1016/j.jfineco.2022.01.004

53 Sun, L. & Abraham, S. Estimating dynamic treatment effects in event studies with heterogeneous treatment effects. *Journal of Econometrics* **225**, 175-199 (2021). https://doi.org:https://doi.org/10.1016/j.jeconom.2020.09.006

Climate policy portfolios that accelerate emission reductions

Theodoros Arvanitopoulos § [1,2]
Simon Bulian § [3,4]
Charlie Wilson § * [5,6]
Andrew J. Jordan [7]
Jale Tosun [3,4,8]
Nicholas Vasilakos [9]

§ equally contributing authors
* corresponding author (charlie.wilson@eci.ox.ac.uk)

[1] Cardiff University, Cardiff Business School
[2] London School of Economics, Hellenic Observatory
[3] Heidelberg University, Institute of Political Science
[4] Heidelberg University, Heidelberg Center for the Environment
[5] University of Oxford, Environmental Change Institute
[6] International Institute for Applied Systems Analysis (IIASA)
[7] University of East Anglia (UEA), Tyndall Centre for Climate Change Research
[8] University of Oslo, Department of Political Science
[9] University of East Anglia (UEA), Norwich Business School

SUPPLEMENTARY INFORMATION 2 (SI2): CLIMATE POLICY VIGNETTES OF SELECT COUNTRIES

1 COUNTRY SELECTION STRATEGY

2 EFFECT OF CLIMATE POLICY DENSITY

2.1 United States of America

2.2 China

2.3 Brazil

3 EFFECT OF POLICY SECTORAL COVERAGE

3.1 Israel

3.2 Russia

3.3 Norway

4 EFFECT OF POLICY INSTRUMENT TYPE

4.1 Estonia

4.2 Israel

4.3 Indonesia

5 EFFECT OF EMISSION REDUCTION TARGETS

5.1 The United States of America

5.2 Mexico

6 EFFECT OF ENERGY MINISTRIES

6.1 United States of America

7 EFFECT OF INDEPENDENT ADVISORY BODIES

7.1 United Kingdom

8 SUPPLEMENTARY REFERENCES

1 Country selection strategy

We selected countries for policy vignettes to help interpret the results of the econometric analysis (Table S24). We grouped countries into terciles (Fast, Medium, Slow) on the dependent variable: emission intensity reductions. We grouped countries into terciles (High, Medium, Low) on each of the independent variables: climate policy density, policy sectoral coverage (diversity index), policy instrument type (diversity index). We then selected countries in the High group on the independent variable and the Fast group on the dependent variable (for examples) or the Slow group on the dependent variable (for counter-examples).

Table S24. Summary of country selection.

To illustrate policy density effect	To illustrate policy sectoral coverage effect	To illustrate policy instrument type effect [1]	To illustrate targets and institutions interaction effect
USA<br>(High policy density, Fast emission intensity reduction)	ISRAEL<br>(High energy sector specialisation, Fast emission intensity reduction)	ESTONIA<br>(High economic instrument type specialisation, Fast emission intensity reduction)	USA<br>(High policy density and absolute emissions targets and energy ministry, Fast emission intensity reduction)
CHINA<br>(High + fast catchup in policy density, Medium emission intensity reduction)	RUSSIA<br>(High buildings sector specialisation, Fast emission intensity reduction)	ISRAEL<br>(High regulatory instrument type specialisation, Fast emission intensity reductions)	UK<br>(High policy density and independent body, Fast emission intensity reduction)
BRAZIL<br>(Low policy density, Slow emission intensity reduction)	NORWAY<br>(Low sectoral specialisation, Slow emission intensity reduction)	INDONESIA<br>(Low instrument type specialisation, Slow emission intensity reduction)	MEXICO<br>(High policy density, no absolute emissions targets, Slow emissions intensity reduction)

[1] Voluntary instruments comprise 23% of the sample so are not included.

2 Effect of climate policy density

Our econometric analysis indicates a positive association between the overall climate policy density of a country and its carbon emission intensity. Below, we illustrate this pattern with selected country vignettes. For this purpose, we portray two countries with High climate policy density and relatively Fast emission intensity reduction (USA and China) and one country with Low climate policy density and relatively Slow emission intensity reduction (Brazil).

2.1 United States of America

In the USA, increasing policy density has coincided with relatively Fast carbon emission intensity reductions.

Figure S6. Instrument types and changes in emission intensity per sector in the United States. *Carbon emission intensity is calculated as sectoral emissions from direct fossil fuel use only divided by whole economy GDP (blue & red dots, upper x-axis). The lower x-axis shows the number of adopted climate policies (climate policy density) by different types of policy instrument (grey-scale bars) in 2022.*

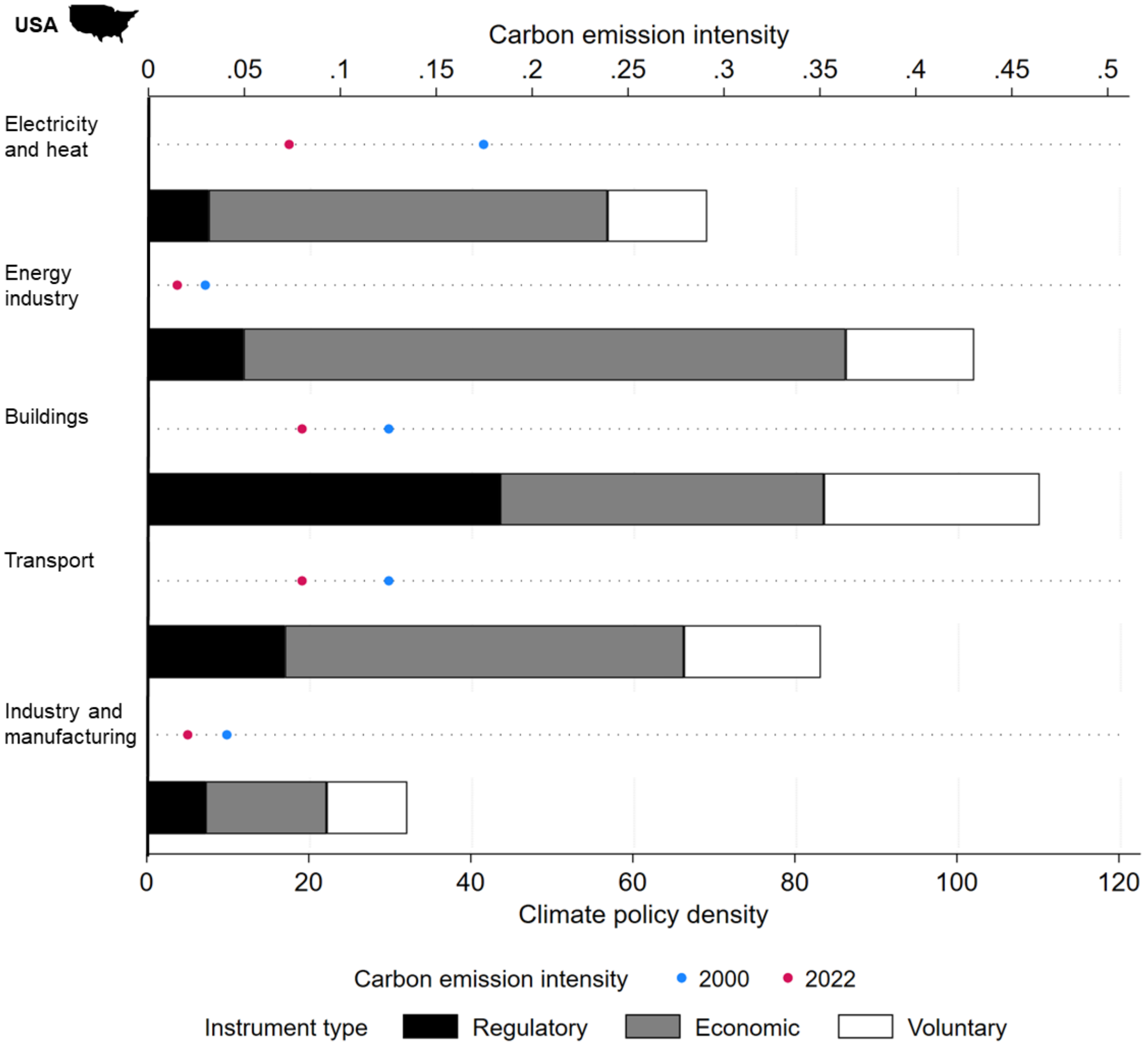

Note: Based on policy data from the IEA Policies and Measures Database (PMD) and carbon emission data from the IEA CO2 Emissions From Fuel Combustion Database.

Historically, the electricity and heat production sectors have contributed most to the USA's GHG emissions, followed by transport and buildings. The country was especially successful in significantly decreasing emissions from electricity and heat. The decrease can be traced back to a significant reduction in the combustion of coal and oil and a strong uptake of natural gas and renewable energy [1].

Figure S7. Historical development of the United States' climate policy portfolio. *Carbon emission intensity is calculated as sectoral emissions from direct fossil fuel use only divided by whole economy GDP (red line, right-hand y-axis). Climate policy density is calculated as cumulative number of adopted climate policies (blue line, left-hand y-axis).*

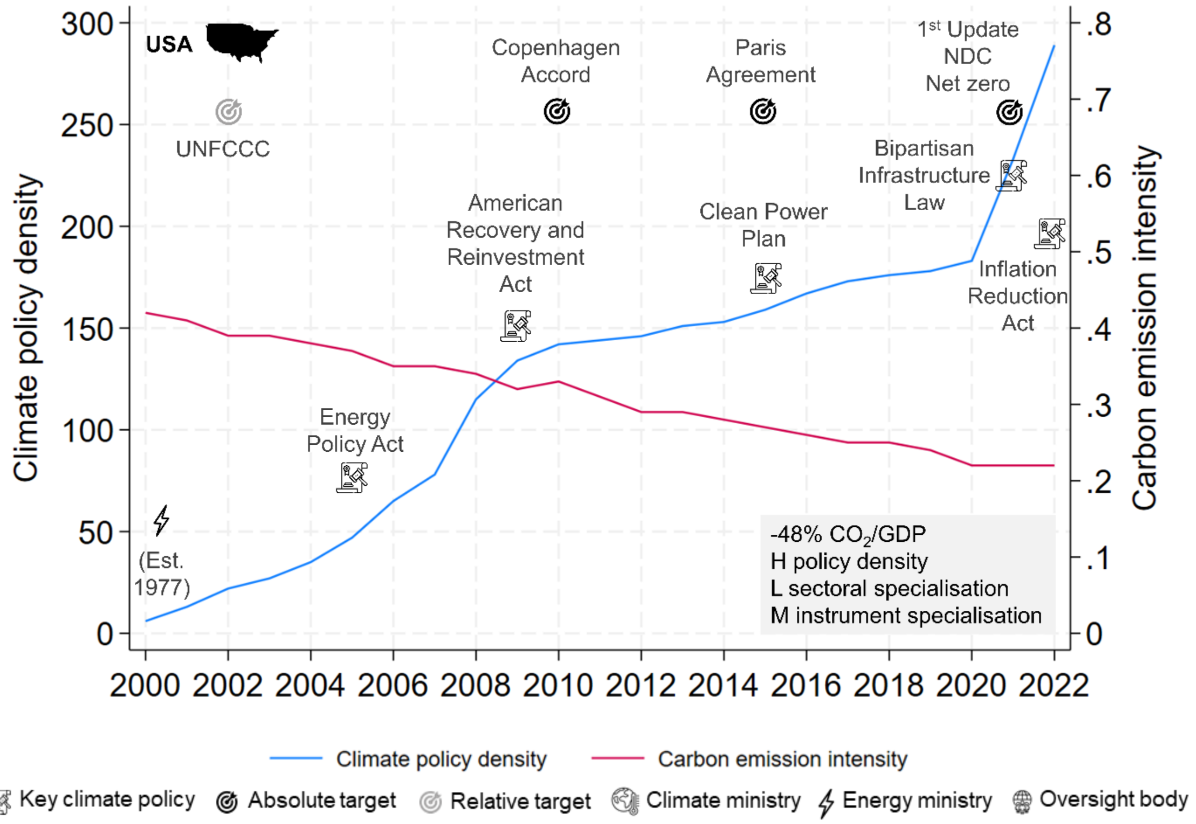

Note: Based on policy data from the IEA Policies and Measures Database (PMD) and carbon emission data from the IEA CO2 Emissions From Fuel Combustion Database. Data on targets, energy ministries and advisory bodies retrieved from various sources (see Table S1 in SI1: Data & Models).

Key federal climate policies in the USA since 2000 include the 2005 Energy Policy Act and the 2009 American Recovery and Reinvestment Act. These two have been identified as key policies fostering renewable energy production and improving energy efficiency [2,3]. The Clean Power Plan adopted in 2015 represents a further key federal climate policy. It triggered the adoption of state-level policies that promoted the expansion of renewable energy and reduced emissions from coal and oil power plants [3,4]. The USA greatly expanded their climate policy under president Joe Biden since 2021. Key legislations include the Bipartisan Infrastructure Law (BIL) from 2021 and the Inflation Reduction Act (IRA) adopted in 2022. Together, these acts have mobilized the highest level of climate investment in US history and mostly aim at expanding renewable energy [5].

Overall, the USA exemplifies the positive association between the adoption of energy related climate policies, a reduction in energy generation from oil and coal, an expansion of renewable energy and equivalent carbon emission reductions in the electricity and heat sector.

2.2 China

China's carbon emission intensity has been in steady decline since 2005. This trend has coincided with a sharp increase in climate policy adoption. According to the IEA, China had adopted 245 climate policies and measures in 2022, a number which is only exceeded by the USA.

Figure S8. Instrument types and changes in emission intensity per sector in China. *Carbon emission intensity is calculated as sectoral emissions from direct fossil fuel use only divided by whole economy GDP (blue & red dots, upper x-axis). The lower x-axis shows the number of adopted climate policies (climate policy density) by different types of policy instrument (grey-scale bars) in 2022.*

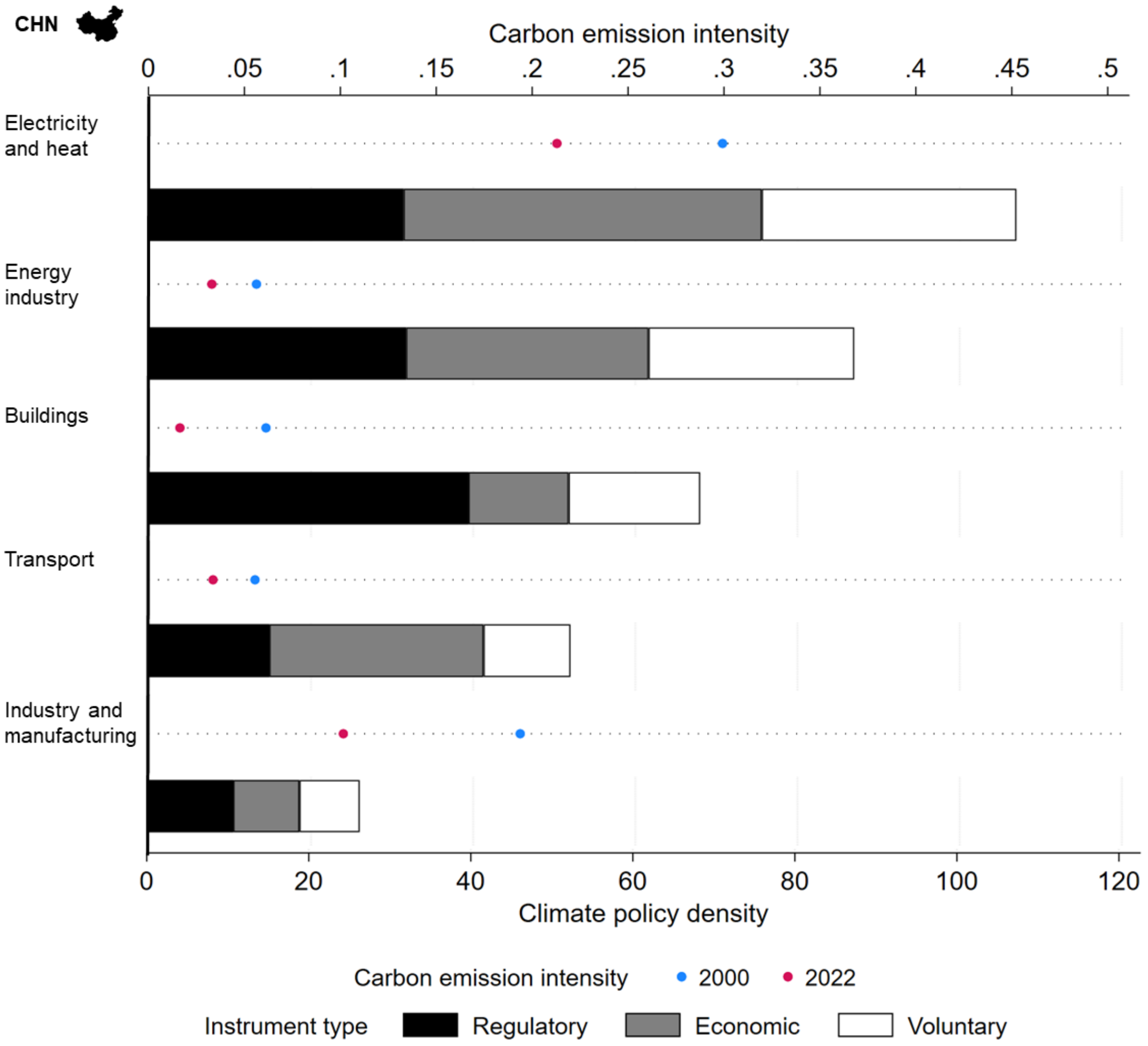

Note: Based on policy data from the IEA Policies and Measures Database (PMD) and carbon emission data from the IEA CO2 Emissions From Fuel Combustion Database.

While China was successful in improving its carbon emission intensity, its greenhouse gas emissions have grown by more than 75% since 2000 due to its strong economic growth and energy demand. Historically, emissions from electricity and heat have contributed the largest share, followed by manufacturing and construction. Combustion of coal has been the largest driver of emissions from the energy sector. While energy generation from coal grew in

absolute terms, its relative share decreased significantly due to a significant expansion of renewable energies [6].

Figure S9. Historical development of China's climate policy portfolio. *Carbon emission intensity is calculated as sectoral emissions from direct fossil fuel use only divided by whole economy GDP (red line, right-hand y-axis). Climate policy density is calculated as cumulative number of adopted climate policies (blue line, left-hand y-axis).*

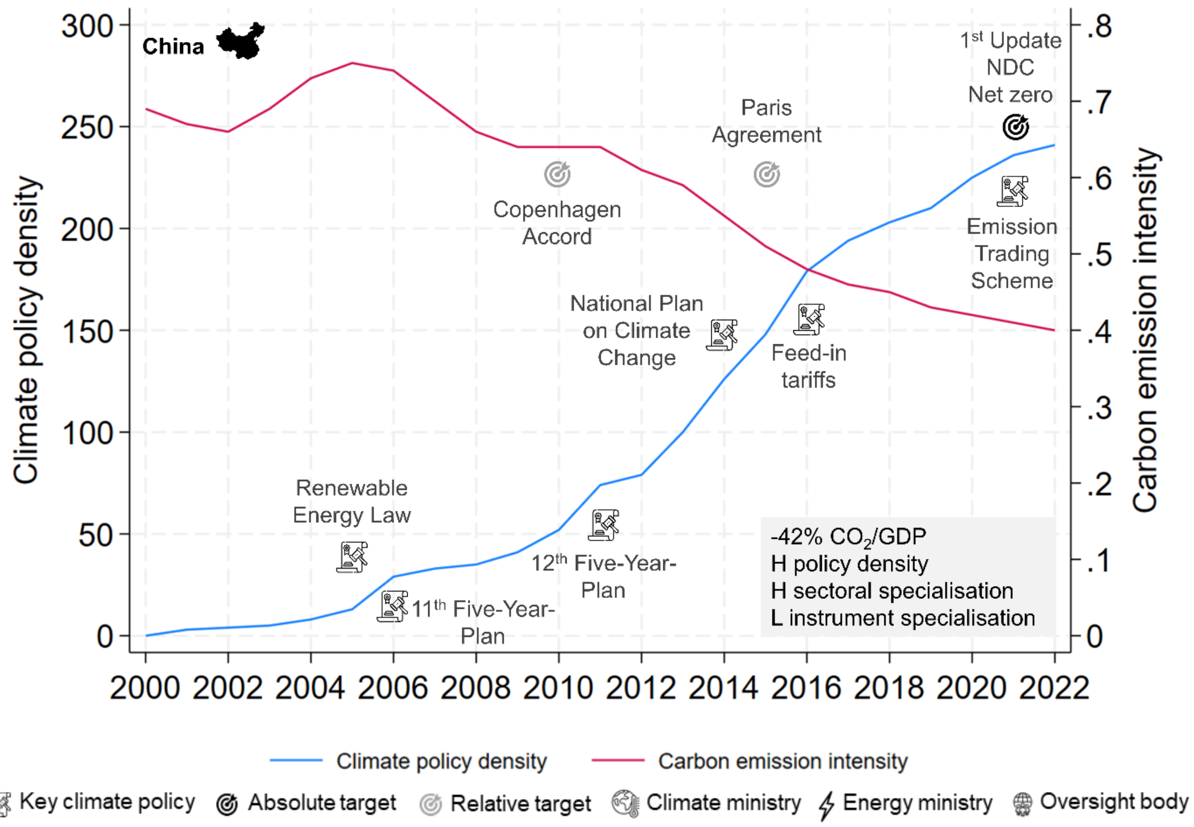

Note: Based on policy data from the IEA Policies and Measures Database (PMD) and carbon emission data from the IEA CO2 Emissions From Fuel Combustion Database. Data on targets, energy ministries and advisory bodies retrieved from various sources (see Table S1 in SI1: Data & Models).

China's policy portfolio has a broad sectoral coverage [7]. After ratifying the Kyoto Protocol in 2002, China adopted a Renewable Energy Law in 2005, which included renewable energy targets and established feed-in-tariffs for renewable energy. The 11th Five-Year-Plan (2006-2010) added the first national target for improving energy efficiency, including instructions for subnational political elites to implement measures to achieve them. In response to the financial crisis in 2008, China adopted an economic stimulus, which included financial support for the solar power manufacturing industry. Climate policy ambition increased with the 12th Five-Year-Plan (2011-2015), which included an emission reduction target and led to the publication of a climate work plan and the National Plan on Climate Change (2014-2020). In 2015, China submitted an Intended-Determined Contribution (INDC) to the UNFCCC, which included several more specific climate targets. After ratifying the Paris Agreement, China implemented several measures to increase renewable energy, such as feed-in-tariffs in 2016. Since 2021, China has an emission trading scheme for the energy sector in place and aims to achieve carbon neutrality before 2060 [7]. China's ambition to reduce its carbon dioxide emissions will be challenging to achieve given its equally ambitious industrial development goals; it is telling that its climate policies have been criticised for not

significantly reducing GHG emissions fast enough to achieve the goals set by the Paris Agreement [8,9].

Overall, our analysis suggests that Chinese policy efforts have led to decreased carbon emission intensity despite the strong growth in absolute emissions. This applies especially for the energy sector. The Chinese government has implemented several policies to reduce emissions in this sector, which has contributed to improved efficiency and a reduction of carbon dioxide emissions from the energy sector [10].

2.3 Brazil

Climate mitigation in Brazil has been characterised by a comparatively Slow increase in climate policy density and simultaneously Slow reduction in emission intensity.

Figure S10. Instrument types and changes in emission intensity per sector in Brazil. *Carbon emission intensity is calculated as sectoral emissions from direct fossil fuel use only divided by whole economy GDP (blue & red dots, upper x-axis). The lower x-axis shows the number of adopted climate policies (climate policy density) by different types of policy instrument (grey-scale bars) in 2022.*

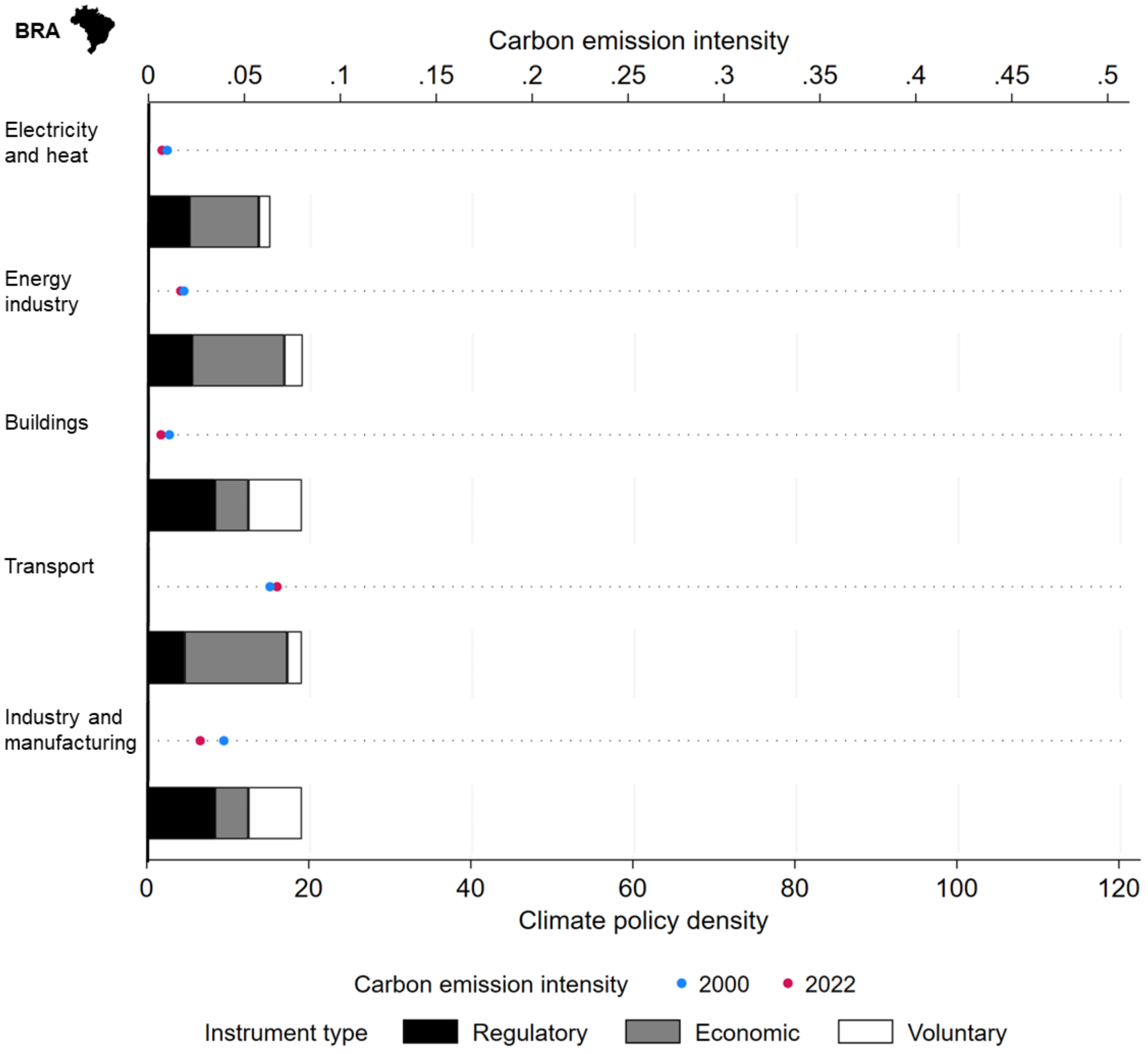

Note: Based on policy data from the IEA Policies and Measures Database (PMD) and carbon emission data from the IEA CO2 Emissions From Fuel Combustion Database.

Greenhouse gas emissions from mostly all sectors in Brazil have increased over the last decades. The main driver of carbon emissions has been deforestation [11]. By some margin, emissions from transport represent the second largest source of greenhouse gas emissions and have also increased over time. In addition, energy consumption in Brazil grew significantly in the last two decades. Especially the combustion of oil, amplified by the discovery of deep indigenous oil reserves in 2011, has contributed to increasing emissions from the electricity and heat sector [11].

Figure S11. Historical development of Brazil's climate policy portfolio. *Carbon emission intensity is calculated as sectoral emissions from direct fossil fuel use only divided by whole economy GDP (red line, right-hand y-axis). Climate policy density is calculated as cumulative number of adopted climate policies (blue line, left-hand y-axis).*

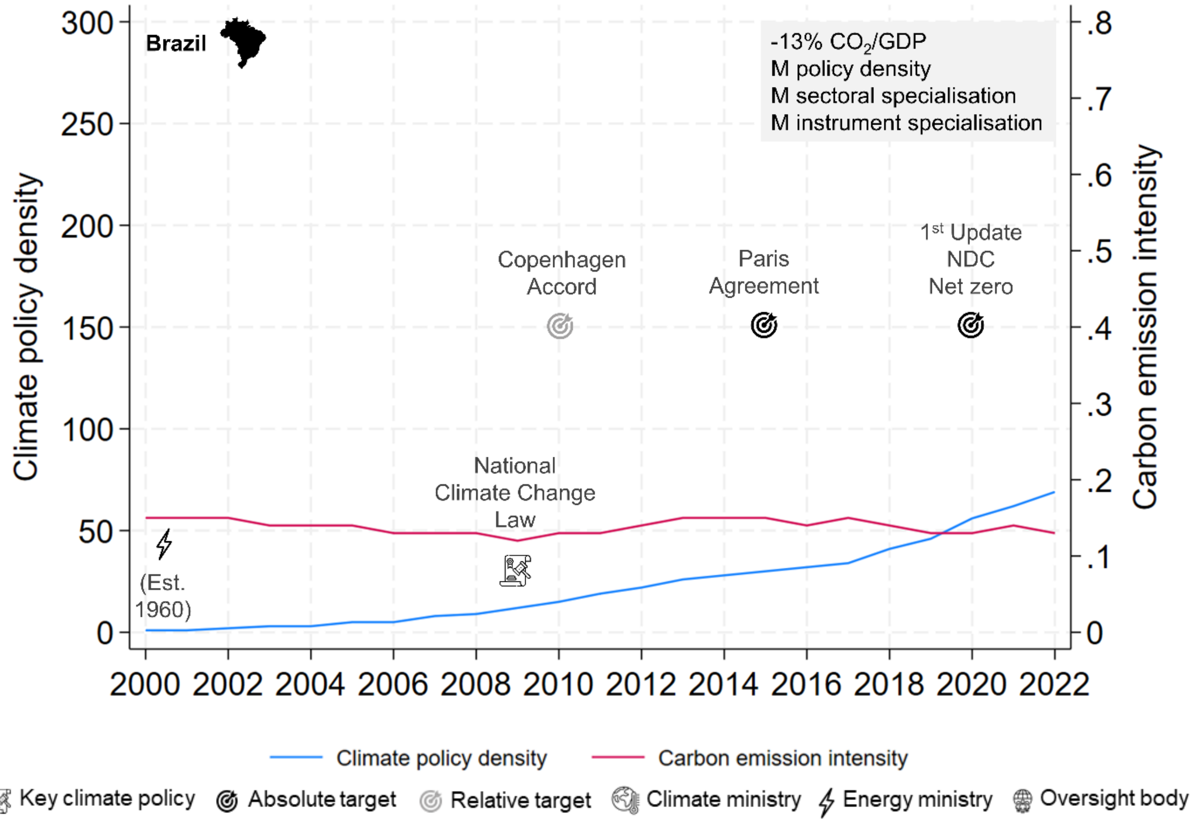

Note: Based on policy data from the IEA Policies and Measures Database (PMD) and carbon emission data from the IEA CO2 Emissions From Fuel Combustion Database. Data on targets, energy ministries and advisory bodies retrieved from various sources (see Table S1 in SI1: Data & Models).

With regard to policy efforts to mitigate climate change, Brazil has experienced different phases since 2000. In the beginning of the 21st century, Brazil witnessed increasing policy efforts to reduce carbon emissions under the first Lula administration [11]. Most prominently, it adopted the National Climate Change Law in 2009, which represents the country's flagship climate legislation. These efforts mostly addressed emissions from deforestation and neglected other sectors [3]. Nevertheless, they are largely responsible for the reduction in carbon emission intensity until 2009 [11]. After 2011, Brazil experienced a sharp U turn in its policy efforts. Under the Rousseff and Temer administrations, existing climate policy was significantly weakened [12]. The situation worsened under Bolsonaro's presidency between

2016 and 2022, which saw no further expansion of climate policy portfolios and a weakening of the institutional and legal framework for reducing deforestation [12].

Overall, Brazil represents an exemplary case where a Slow increase in climate policy density coincided with no reduction in carbon emission intensity. This applies especially for the 2010s, where the country dropped its policy efforts to halt deforestation (Brazil's largest driver of carbon emissions) and neglected to adopt ambitious policies to reduce emissions from transport or energy.

3 Effect of policy sectoral coverage

Our econometric analysis indicates that countries that concentrated their climate policies on specific sectors experienced relatively Fast reductions in carbon emission intensity. In the following, we present three exemplar cases for this pattern. In two cases, High policy specialisation on one sector correlates with relatively Fast carbon emission intensity reduction (Israel and Russia). In one case (Norway), Low sector specialisation is associated with relatively Slow emission intensity reduction.

3.1 Israel

Although Israel adopted significantly fewer climate policies compared to other states (30 policies by 2022), its carbon emission intensity has decreased since 2000. This seemingly contradictory finding can be explained by the country's focus on the adoption of climate policies targeting the energy sector, which has produced the largest share of carbon emissions. Throughout the study period, more than 50% of the country's climate policies addressed electricity and heat production. The increase of such policies over time coincided with a marked reduction in carbon emission intensity in the energy sector.

Historically, the electricity and heat sector has contributed the largest share of carbon dioxide emissions in Israel, followed by some margin by emissions from transport. Combustion of oil and coal have mostly driven the emissions. The discovery of large natural gas reserves in Israel in 2009 led to a strong increase in energy generation from gas and a subsequent reduction in coal combustion. Renewable energy has contributed only a minor share to the overall energy mix so far.

Policies adopted in Israel to reduce carbon emissions in the energy sector mostly include those supporting the uptake of renewable energy. They included adopting a subsidy for electricity generation from wind and photo-voltaic in 2004, introducing a feed-in-tariff for solar and wind power in 2008 and launching a solar tendering round in 2017. Whereas the first two created financial incentives to invest in renewable energy, the later reduced bureaucratic burdens [13]. The Israeli government proposed introducing a carbon tax in 2021, but has not yet implemented the policy measure [14].

The renewable energy production sector has indeed increased in recent years, mostly with regard to solar energy [13]. Nevertheless, the share of renewable energy generation in Israel still remains very Low compared to other OECD countries: only 5% of electricity was produced by renewables in 2021 [14]. In fact, the decrease in carbon emissions from the energy sector can mostly be explained by the replacement of coal combustion by natural gas. The discovery of indigenous natural gas also hampered renewable energy expansion which has only recently gained new momentum [14,15].

Figure S12. Instrument types and changes in emission intensity per sector in Israel. *Carbon emission intensity is calculated as sectoral emissions from direct fossil fuel use only divided by whole economy GDP (blue & red dots, upper x-axis). The lower x-axis shows the number of adopted climate policies (climate policy density) by different types of policy instrument (grey-scale bars) in 2022.*

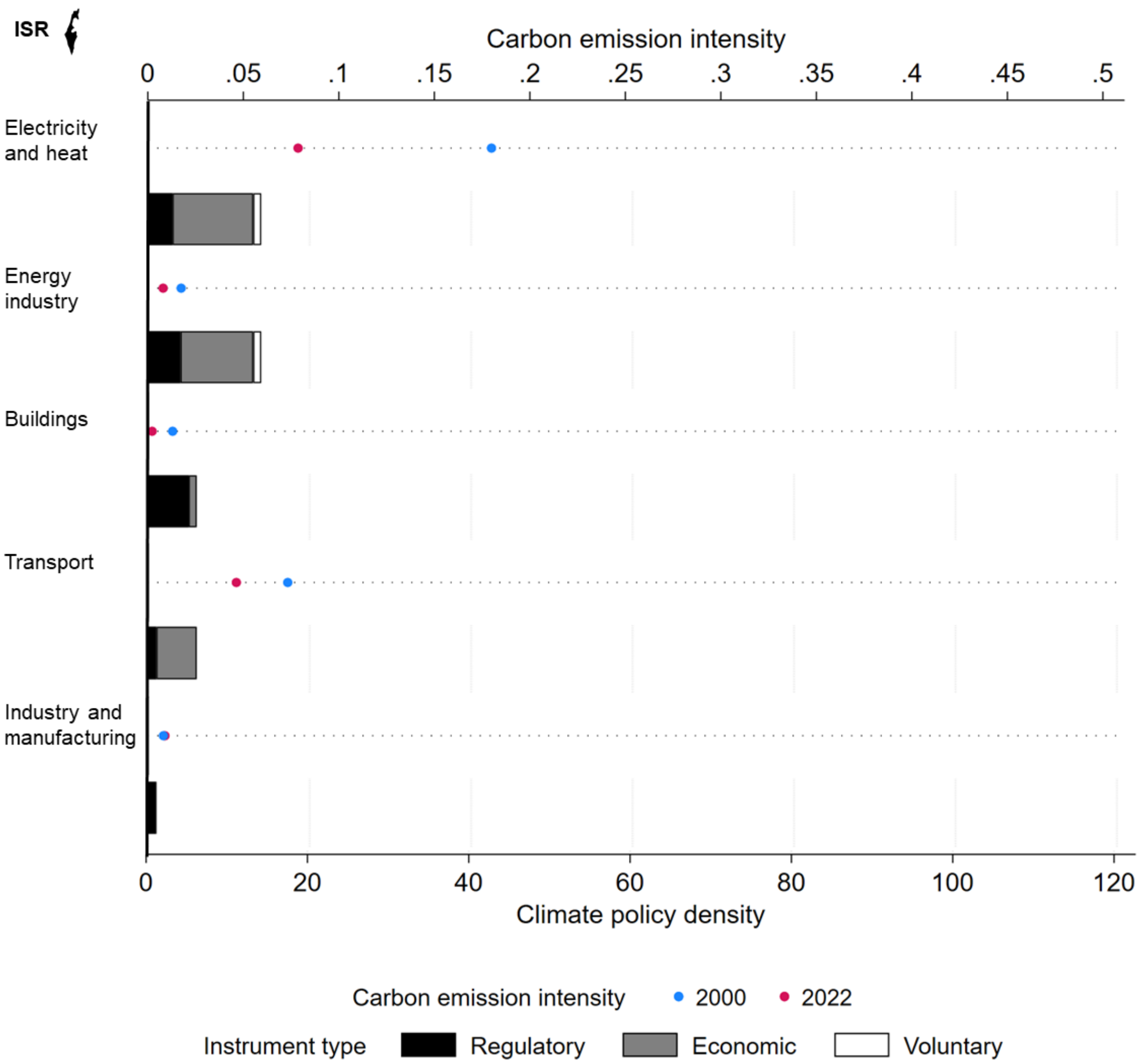

Note: Based on policy data from the IEA Policies and Measures Database (PMD) and carbon emission data from the IEA CO2 Emissions From Fuel Combustion Database.

Figure S13. Historical development of Israel's climate policy portfolio. *Carbon emission intensity is calculated as sectoral emissions from direct fossil fuel use only divided by whole economy GDP (red line, right-hand y-axis). Climate policy density is calculated as cumulative number of adopted climate policies (blue line, left-hand y-axis).*

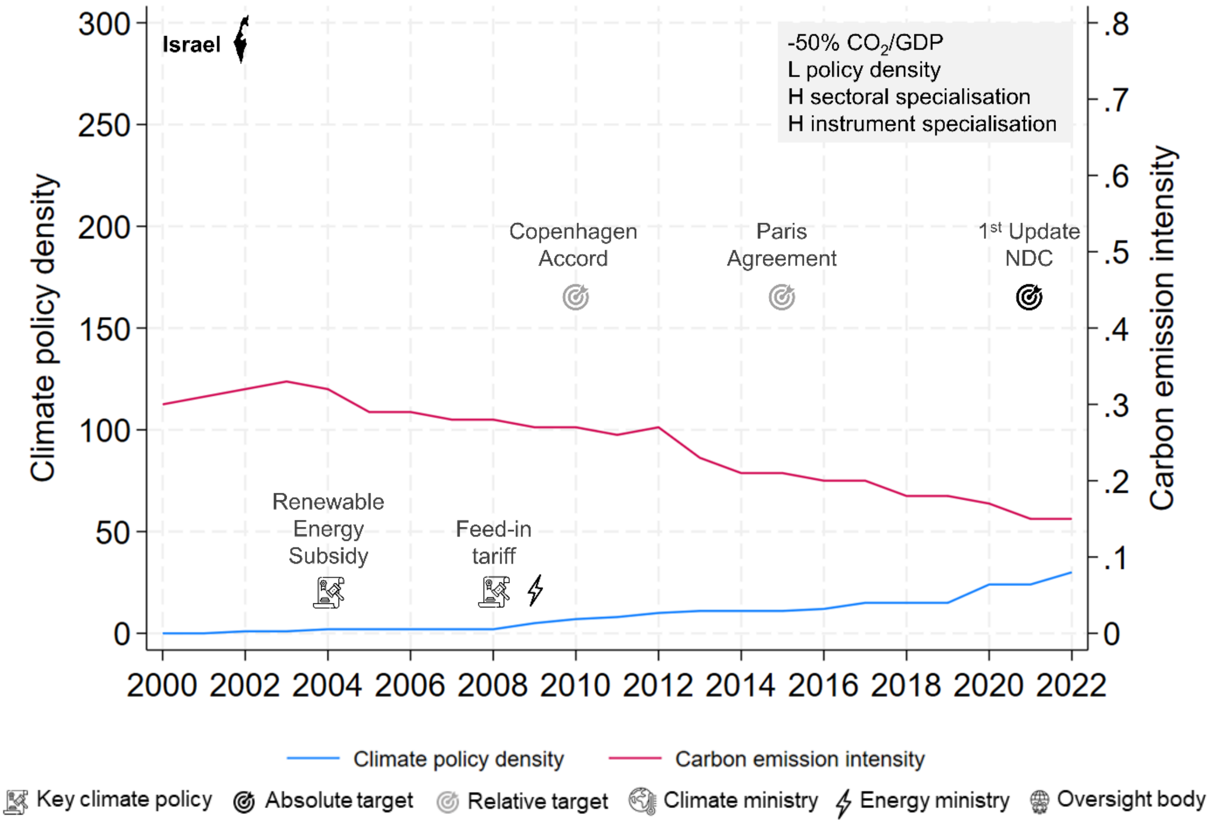

Note: Based on policy data from the IEA Policies and Measures Database (PMD) and carbon emission data from the IEA CO2 Emissions From Fuel Combustion Database. Data on targets, energy ministries and advisory bodies retrieved from various sources (see Table S1 in SI1: Data & Models).

Overall, Israel's decline in carbon emission intensity is largely a result of emission reduction from the energy sector, which has contributed the largest share of the country's carbon emissions. Israel's climate policies have focused on that sector primarily to promote the expansion of renewable energy. While energy generation from renewables has increased, the relatively strong decrease in emission intensity is mostly due to energy generation switching from coal to natural gas.

3.2 Russia

Russia provides a counter example of concentrated but misaligned policy coverage with >80% of all policies targeting the buildings sector while emissions predominantly came from the two upstream energy sectors (60% of 2000-2022 emissions). Building sector policies accumulated in the period 2008-2015 which also post-dates observed reductions in emission intensity. The country has a Low energy efficiency in almost all sectors and thus its level of carbon emission intensity is comparatively High.

Figure S14. Instrument types and changes in emission intensity per sector in Russia. *Carbon emission intensity is calculated as sectoral emissions from direct fossil fuel use only divided by whole economy GDP (blue & red dots, upper x-axis). The lower x-axis shows the number of adopted climate policies (climate policy density) by different types of policy instrument (grey-scale bars) in 2022.*

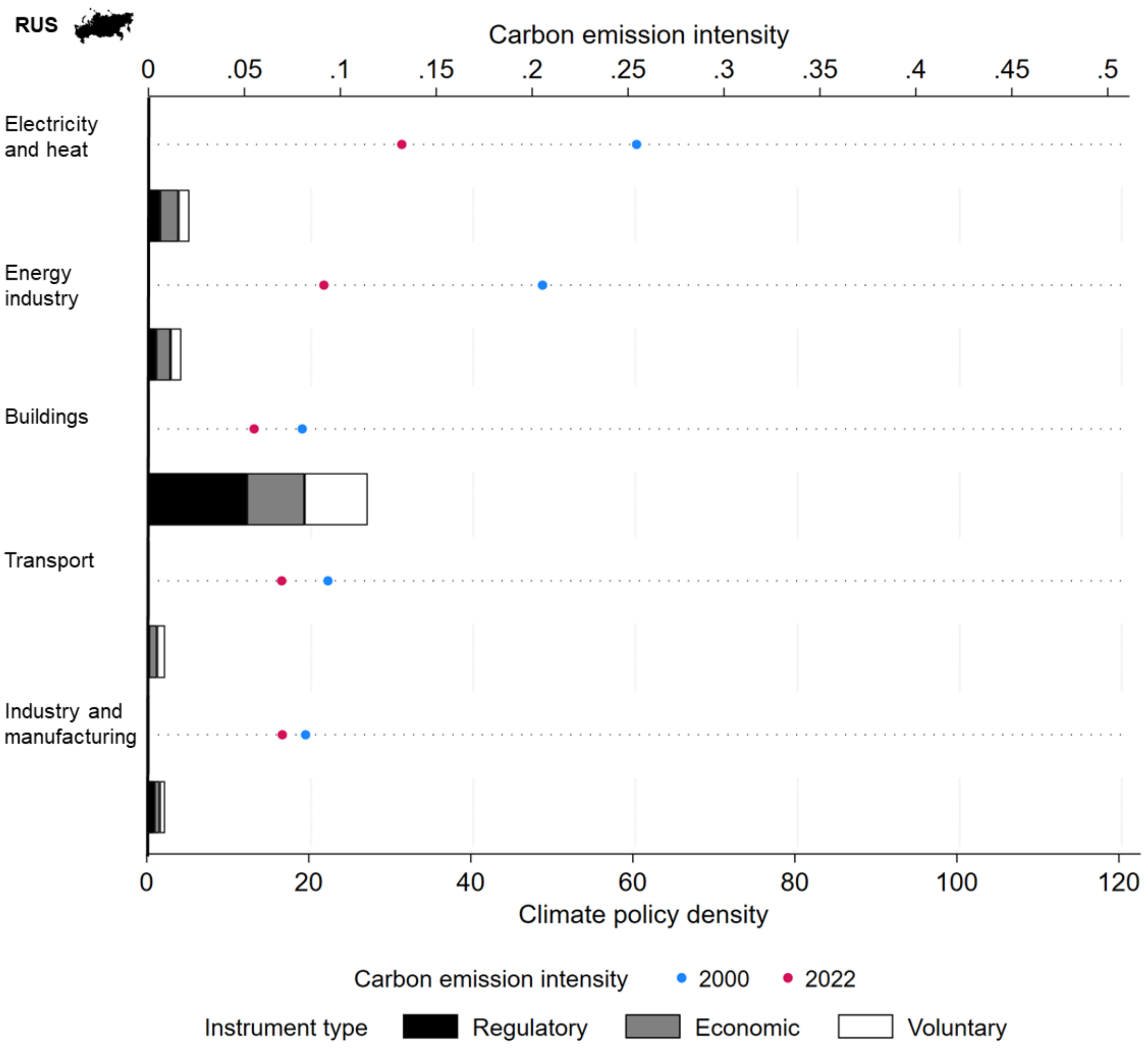

Note: Based on policy data from the IEA Policies and Measures Database (PMD) and carbon emission data from the IEA CO2 Emissions From Fuel Combustion Database.

Russia has one of the largest shares of global carbon emissions in the world, which is mostly due to the heavy utilization of fossil fuels for energy generation. Today, solar and wind energy account for less than 1% of electricity generation. Reduction in carbon emission intensity until 2009 is mostly due to the economic recession in the 1990s [16]. Since 2009, the level of emission intensity has stagnated [17].

Figure S15. Historical development of Russia's climate policy portfolio. *Carbon emission intensity is calculated as sectoral emissions from direct fossil fuel use only divided by whole economy GDP (red line, right-hand y-axis). Climate policy density is calculated as cumulative number of adopted climate policies (blue line, left-hand y-axis).*

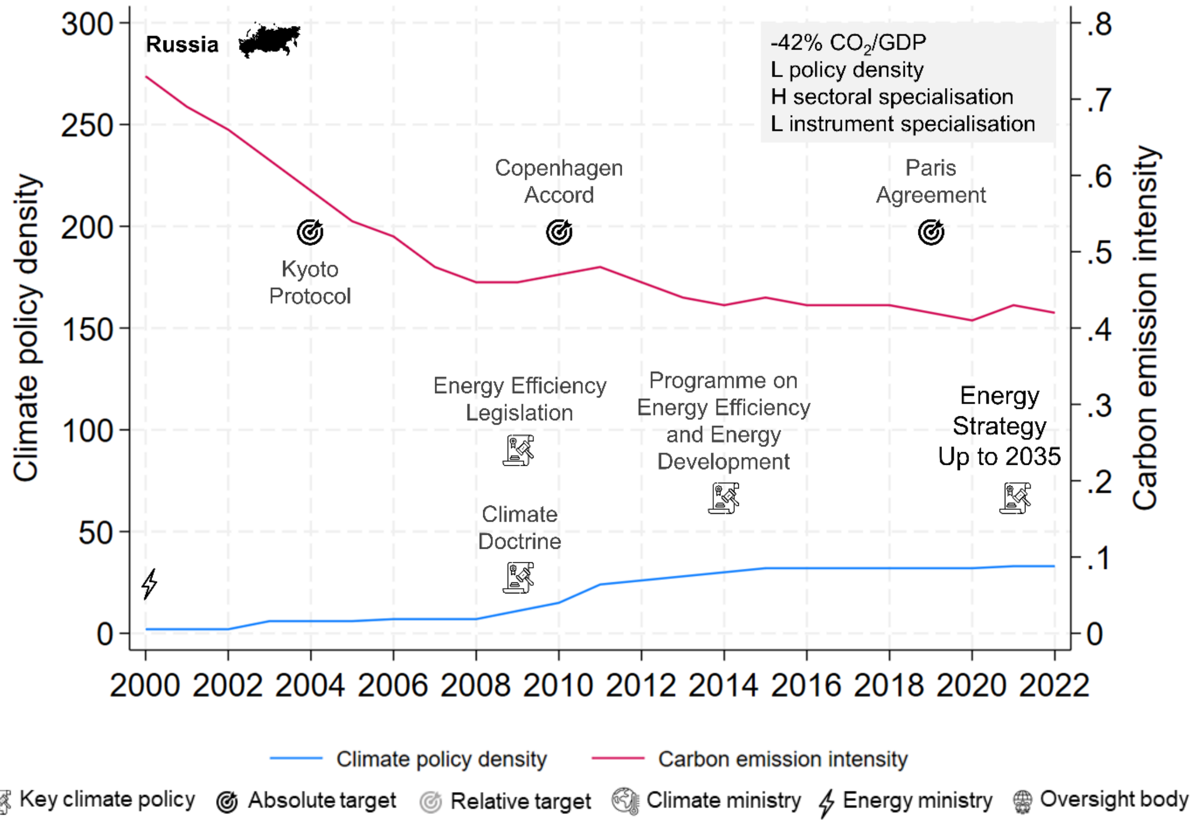

Note: Based on policy data from the IEA Policies and Measures Database (PMD) and carbon emission data from the IEA CO2 Emissions From Fuel Combustion Database. Data on targets, energy ministries and advisory bodies retrieved from various sources (see Table S1 in SI1: Data & Models).

Despite ratifying the Kyoto Protocol in 2004 and the Paris Agreement in 2019, Russia's policy efforts to decarbonize its economy have been very limited [17,18]. Key Russian climate policies have included the non-binding Climate Doctrine, adopted in 2009, which provided guidelines and set climate-related targets. In the same year, it adopted the Energy Efficiency Legislation, which aimed at energy saving and increasing energy efficiency. The legislation was updated in 2014 by the State Programme on Energy Efficiency and Energy Development, which aimed specifically to increase the efficiency of the use of Russian fuel and energy resources. In 2015, the Energy Ministry submitted a draft for the Russian Energy Strategy Up to 2035, which took six years to be finally adopted in 2021. During this time, its ambition to mitigate Russia's greenhouse gas emission had been significantly reduced. It now aims to increase the share of non-hydro renewable energies to 4.5% by 2024, which is very unlikely to be achieved [17,18].

Overall, carbon emission intensity has stagnated at a comparatively High level since 2009. Russia's climate policies have focused on emissions from buildings rather than the largest emitting sector – electricity and heat. In fact, Russia since 2009 represents an exemplary case where Low policy focus on the largest emitting sector coincides with comparatively Low emission intensity reduction.

3.3 Norway

Norway is a counter example of a country with broad and balanced sectoral coverage including both economy-wide policies (e.g. carbon tax) and sectoral policies (e.g. emissions trading in the industry sector, electric vehicle incentives in the transport sector). The country has experienced a comparatively Slow emission intensity reduction and a moderate increase of its climate policy portfolio, which has been characterised by a Low sectoral specialisation (i.e., policies have not focused on a specific sector, such as transport). However, the slow reduction in emission intensity has to be seen against the background of a comparatively low level of emission intensity already in 2000.

Figure S16. Instrument types and changes in emission intensity per sector in Norway. *Carbon emission intensity is calculated as sectoral emissions from direct fossil fuel use only divided by whole economy GDP (blue & red dots, upper x-axis). The lower x-axis shows the number of adopted climate policies (climate policy density) by different types of policy instrument (grey-scale bars) in 2022.*

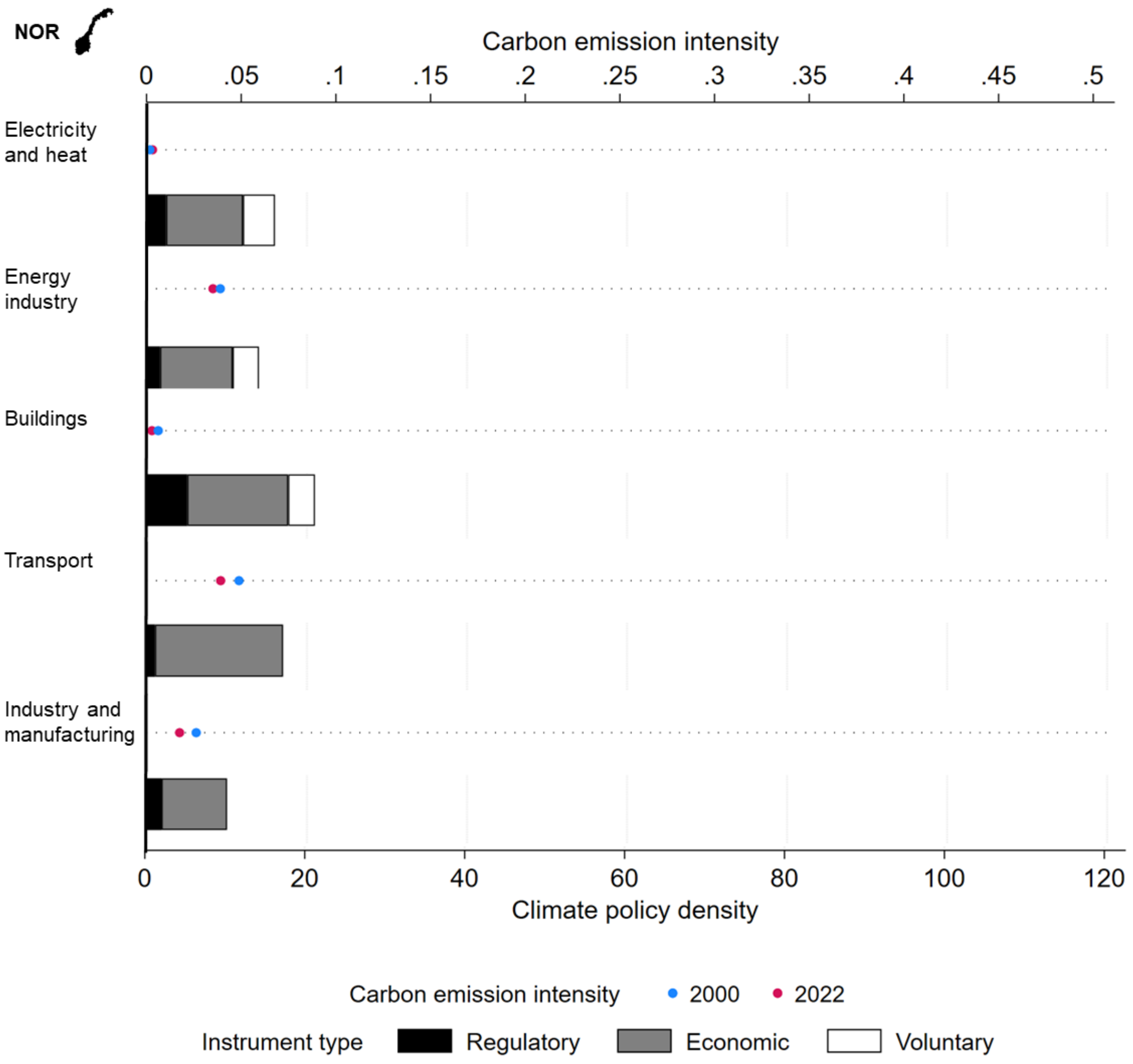

Note: Based on policy data from the IEA Policies and Measures Database (PMD) and carbon emission data from the IEA CO2 Emissions From Fuel Combustion Database.

Most of Norway's energy sector, especially electricity generation, is already heavily decarbonised due to the historically large share of hydropower. Thus, Norway did not experience the same need to target the energy sector than countries with a Higher dependency on energy generated from fossil fuels (e.g., Mexico and Indonesia). Further emission reduction in Norway must therefore mostly be achieved in other sectors, which already have a comparatively Low emission intensity [19]. The largest share of greenhouse gas emissions in the country stems from the oil and gas industry, transport and manufacturing [20].

Figure S17. Historical development of Norway's climate policy portfolio. *Carbon emission intensity is calculated as sectoral emissions from direct fossil fuel use only divided by whole economy GDP (red line, right-hand y-axis). Climate policy density is calculated as cumulative number of adopted climate policies (blue line, left-hand y-axis).*

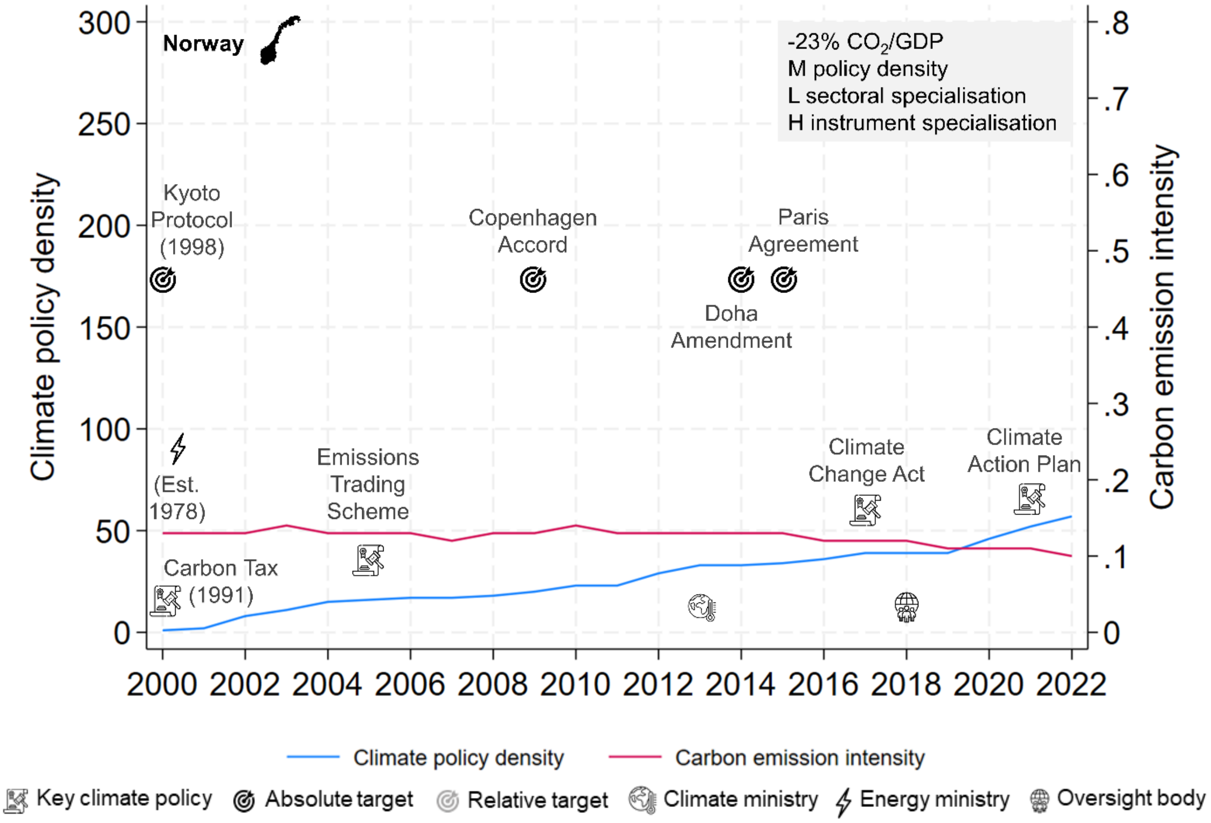

Note: Based on policy data from the IEA Policies and Measures Database (PMD) and carbon emission data from the IEA CO2 Emissions From Fuel Combustion Database. Data on targets, energy ministries and advisory bodies retrieved from various sources (see Table S1 in SI1: Data & Models).

Norway has historically been one of the world's climate policy leaders. It adopted a carbon tax as early as 1991 and significantly increased the carbon price in 2012. More recently, in its 2021 Climate Action Plan, Norway decided to gradually increase the carbon price in the years to 2030.

Norway also established an emission trading scheme in 2005, which was incorporated into the EU Emissions Trading Scheme (ETS) in 2008. To foster the uptake of renewable energy specifically, it created a green certificate system in cooperation with Sweden in 2012. Norway has also targeted the transport sector for two decades by incentivizing the purchase of electric vehicles and heavily taxing cars with internal combustion engines [21]. In 2017, Norway adopted the Climate Change Act, which commits Norway to reduce its greenhouse gas emissions by 55% by 2030 and around 90% by 2050. Since 2019, the country has further

committed to reduce emissions not covered by the ETS by 40% below 2005 levels by 2030 [19].

Overall, these efforts have resulted in a climate policy portfolio that equally targets the different sectors responsible for greenhouse gas emissions.

4 Effect of policy instrument type

Our econometric analysis indicates that countries that have specialised on policy instruments (i.e., have predominantly implemented a specific type of policy instrument) experienced relatively Fast carbon emission intensity. In the following, we present three representative cases for this pattern. In two cases, specialisation on a policy instrument coincided with relatively Fast emission intensity reduction (Estonia and Israel), and in one case, Low specialisation coincided with relatively Slow emission intensity reduction (Indonesia).

4.1 Estonia

Estonia achieved a significant decline in its carbon emission intensity in the period before 2020, which coincided with an increase in its climate policy density.

The country made comparatively High use of economic instruments, such as taxes and/or subsidies. Throughout the study period, more than half of its climate policies were economic policy instruments.

Among OECD countries, Estonia has a relatively carbon intensive economy due to its historically High dependency on oil shale for electricity generation, its High transport emissions and the large stock of old and energy inefficient buildings constructed in the Soviet era [22].

Since becoming an EU member state, Estonia has transposed several EU directives, such as the Energy Efficiency Directive and the Renewable Energy Directive, into national law. In response to the EU's 2020 climate and energy package and 2030 climate framework, Estonia has developed its own national climate strategy to put it on a pathway to achieving a low-carbon economy. Key national climate policies in Estonia include the 'General Principles of Climate Policy until 2050' adopted in 2017, the 2030 National Energy and Climate Plan (NECP) adopted in 2019, and most recently the Strategy Estonia 2035, adopted in 2021, which aims to achieve climate neutrality by 2050 as part of the EU's Long Term Strategy for Net Zero (LTS) [22].

Estonia was one of the first countries in the world to adopt a carbon tax in 2000 [23,24]. Further economic policy instruments include a feed-in tariff adopted in 2007 to promote the expansion of renewable energy, a subsidy program for electric vehicles between 2011 and 2014, and several subsidies to improve the energy efficiency of buildings [22,25]. In addition, Estonia is part of the EU Emission Trading System (ETS), which covers a large share of Estonia's carbon dioxide emissions, mostly from the energy sector [22]. However, Estonia still lacks effective carbon prices in comparison to other OECD countries and continues to subsidize the oil shale industry, which suggests that there is room to intensify the impact of economic instruments on carbon intensity in the future [22,25]. The reversal in emission intensity trends in 2021 seen in Fig S17 is associated with increased reliance on domestic oil shale in the post-Covid recovery, exacerbated by the energy market impact of Russia's invasion of Ukraine in 2022.

Figure S18. Instrument types and changes in emission intensity per sector in Estonia. *Carbon emission intensity is calculated as sectoral emissions from direct fossil fuel use only divided by whole economy GDP (blue & red dots, upper x-axis). The lower x-axis shows the number of adopted climate policies (climate policy density) by different types of policy instrument (grey-scale bars) in 2022.*

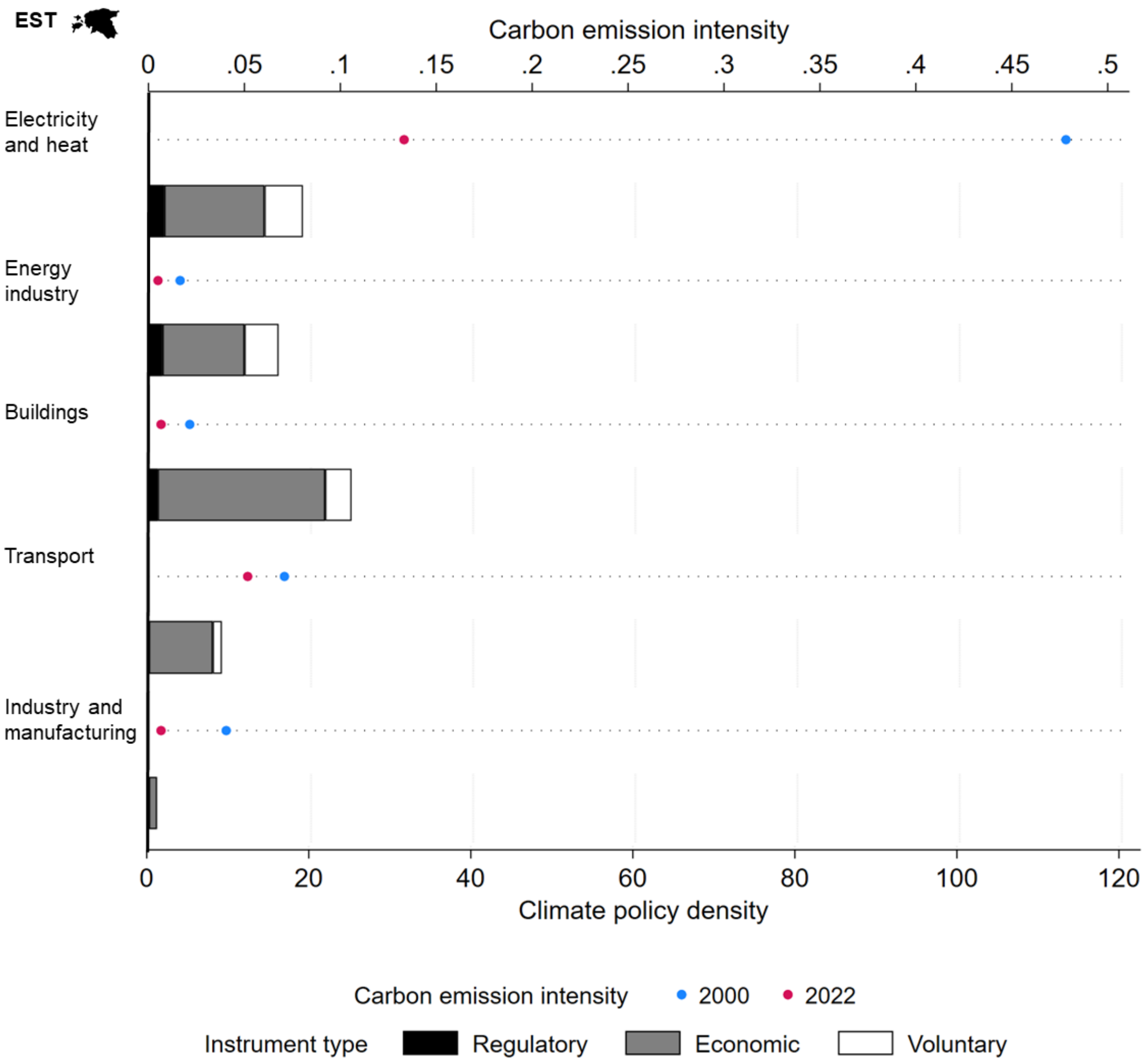

Note: Based on policy data from the IEA Policies and Measures Database (PMD) and carbon emission data from the IEA CO2 Emissions From Fuel Combustion Database.

Figure S19. Historical development of Estonia's climate policy portfolio. *Carbon emission intensity is calculated as sectoral emissions from direct fossil fuel use only divided by whole economy GDP (red line, right-hand y-axis). Climate policy density is calculated as cumulative number of adopted climate policies (blue line, left-hand y-axis).*

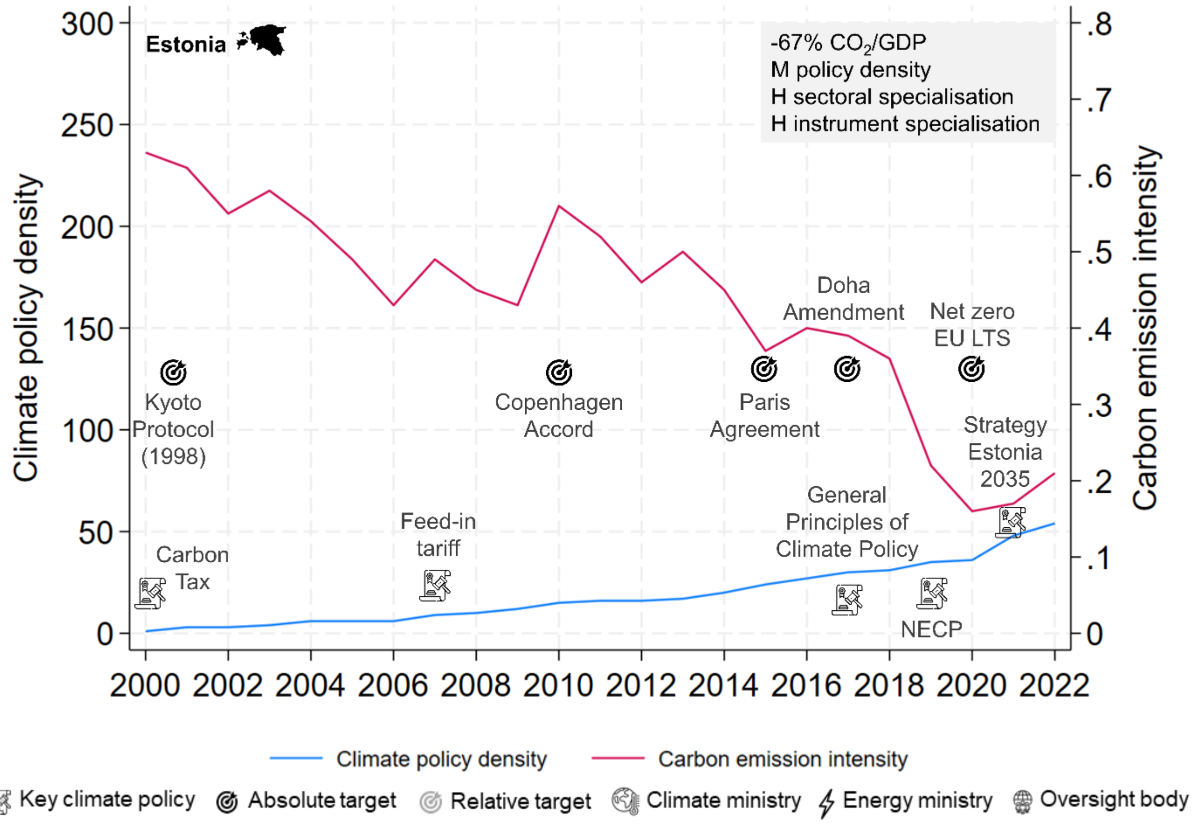

Note: Based on policy data from the IEA Policies and Measures Database (PMD) and carbon emission data from the IEA CO2 Emissions From Fuel Combustion Database. Data on targets, energy ministries and advisory bodies retrieved from various sources (see Table S1 in SI1: Data & Models).

Overall, Estonia has primarily employed economic instruments to mitigate climate change and their adoption coincides with a relatively Fast emission intensity reduction.

4.2 Israel

Israel represents a case where a comparatively strong focus on regulatory instruments has coincided with relatively Fast reduction in emission intensity. Since 2012, more than half of the country's policies employed regulatory instruments. For instance, this included setting up a net-metering regulation framework in 2013, adopting rules for solar energy auctions in 2017 and adopting a set of rules to enhance energy efficiency in buildings in 2011. Overall, these changes mostly served to facilitate the expansion of renewable energy.

However, renewable energies have only played a minor role in reducing emission intensity until 2022. In fact, emission intensity reduction has mostly been attributed to the replacement of coal energy generation by natural gas [15]. Renewable energies still compete with fossil fuels and will need further policy support [14,26].

4.3 Indonesia

The level of emission intensity has remained mostly stable in Indonesia, whereas the number of climate policies has increased steadily. Contrary to Estonia that specialised in a certain type of policy instrument, Indonesia has equally employed regulatory, economic and voluntary instruments.

Energy generation from coal has been identified as the strongest factor explaining the stagnation in carbon dioxide emissions. Enormous coal reserves and a powerful coal industry have inhibited efforts to reduce energy generation from coal and increase the uptake of renewables [27].

Figure S20. Instrument types and changes in emission intensity per sector in Indonesia. *Carbon emission intensity is calculated as sectoral emissions from direct fossil fuel use only divided by whole economy GDP (blue & red dots, upper x-axis). The lower x-axis shows the number of adopted climate policies (climate policy density) by different types of policy instrument (grey-scale bars) in 2022.*

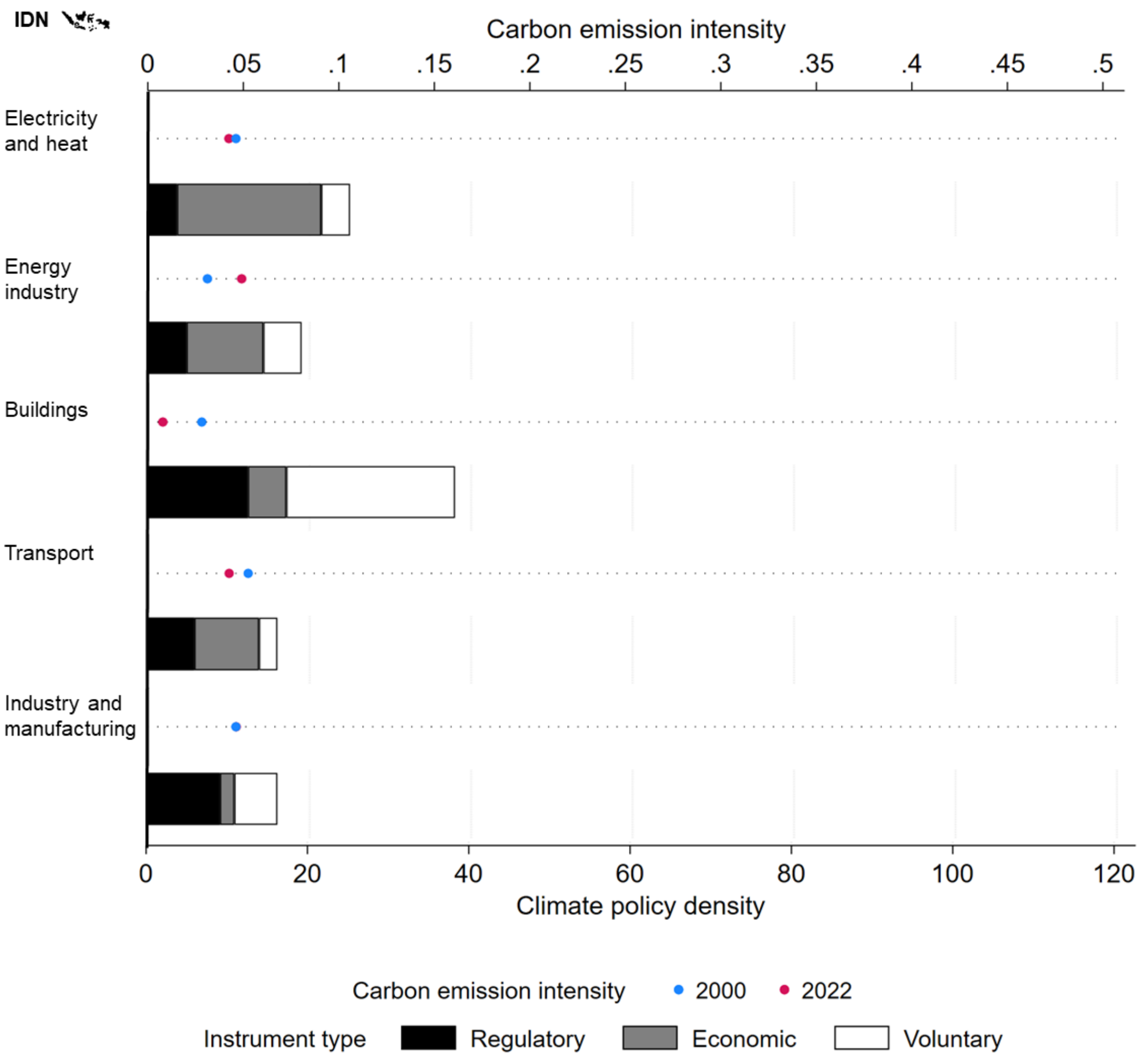

Note: Based on policy data from the IEA Policies and Measures Database (PMD) and carbon emission data from the IEA CO2 Emissions From Fuel Combustion Database.

Figure S21. Historical development of Indonesia's climate policy portfolio. *Carbon emission intensity is calculated as sectoral emissions from direct fossil fuel use only divided by whole economy GDP (red line, right-hand y-axis). Climate policy density is calculated as cumulative number of adopted climate policies (blue line, left-hand y-axis).*

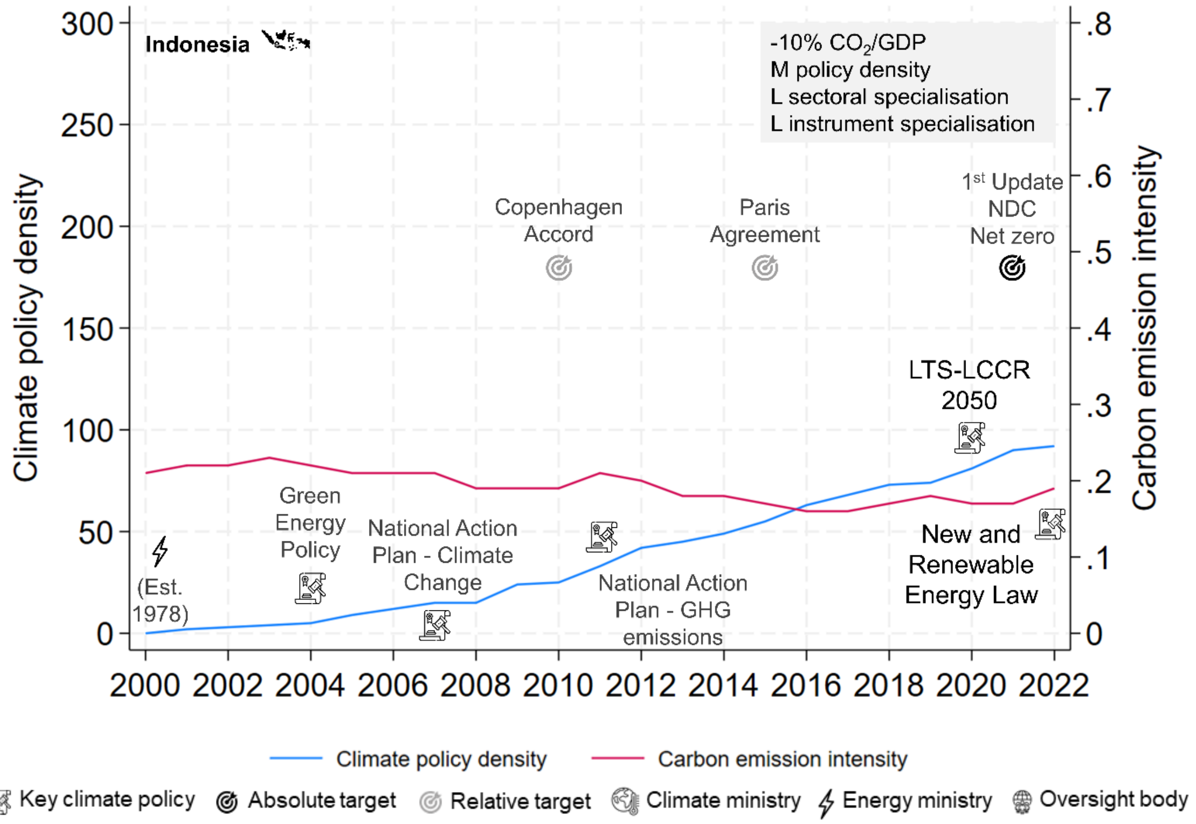

Note: Based on policy data from the IEA Policies and Measures Database (PMD) and carbon emission data from the IEA CO2 Emissions From Fuel Combustion Database. Data on targets, energy ministries and advisory bodies retrieved from various sources (see Table S1 in SI1: Data & Models).

Nevertheless, there have been policy efforts to foster the expansion of renewable energy, including feed-in-tariffs for several renewable energy sources such as solar or biogas since 2009. Key climate policies in Indonesia include the Green Energy Policy adopted in 2004, the National Action Plan Addressing Climate Change in 2007, the National Action Plan to reduce GHG emissions (RAN-GRK) in 2011, the Long-term Strategy on Low Carbon and Climate Resilience (LTS-LCCR) 2050 in 2020 and the New and Renewable Energy Law adopted in 2022 [28].[1]

Until now, the impact of these policies on renewable energy development has been Low [29]. Apart from the strong influence of the coal industry on government decision-making in Indonesia, other governance issues have been identified, such as weak central leadership in developing climate policies and conflicts between various government institutions [27,30].

Overall, Indonesia has developed a climate policy portfolio that, in contrast to other countries such as Estonia, does not specialise in targeting a high-emitting sector or a type of policy instrument. This and issues with policy implementation have coincided with stagnating emission intensity.

---

[1] See also https://climatepolicydatabase.org/countries/indonesia.

5 Effect of emission reduction targets

Our econometric analysis indicates that the positive association between policy density and emission intensity reduction is stronger if a country adopted absolute emission reduction targets. The two cases presented below are representative of this pattern. In the case of the USA, the presence of absolute emission reduction targets since 2010 coincides with a relatively strong association between climate policy density and emission intensity reduction. Whereas in Mexico such targets have been lacking and the relatively strong increase in climate policy density coincides with only relatively Slow emission intensity reduction.

5.1 The United States of America

The USA is an exemplary case for this pattern, at least for the second half of our study period. In the 2000s, the USA did not possess a legally-binding absolute emission reduction target. Under the Bush administration, the USA only adopted an emission intensity target of cutting GHG emissions intensity (ratio of GHG emissions to GDP) by 18 percent by 2012 in 2002. While a target was in place, it did not specify absolute emission reduction levels [31,32]. The situation changed under the Obama administration: in 2010, the USA adopted an absolute emission target of 17 percent below 2005 levels in 2020. In addition, sector specific targets were adopted, most prominently reducing emissions in the power sector of 32 percent below 2005 levels by 2030 within the Clean Power Plan in 2015 [33]. In 2021, the USA submitted a net zero target to the UNFCCC that aims to achieve net zero GHG emissions by 2050.

Overall, the presence of absolute emission reduction targets from 2010 coincides with the strong uptake in climate policies and simultaneous reduction in carbon emission intensity in the USA.

5.2 Mexico

In contrast to the USA, Mexico has only slowly reduced its carbon emission intensity despite a comparatively strong increase in its climate policy density. In fact, Mexico's CO_2 emissions grew over the last decade with transport and electricity generation being the largest emitters. The latter is strongly reliant on fossil fuels, mainly natural gas and oil, due to Mexico's long legacy of oil dependence. Only recently have renewables increased and then quite slowly [34,35].

On paper, Mexico has signalled a High level of policy ambition to reduce its carbon emissions. The country ratified the UNFCCC as early as 1993 and the Kyoto Protocol in 2000. It was the first developing economy to submit an intended Nationally Determined Contribution (NDC) and pledged to reduce its greenhouse gas emissions voluntarily [34,36]. With the 2012 General Law on Climate Change, Mexico was one of the first countries that adopted a climate change act, which included longer-term objectives for climate policy [34,36]. In 2015, Mexico adopted the Energy Transition Law, which is based on its climate change act and outlines the transition of the energy sector. It was modified in 2018 to accommodate for Mexico's NDCs submitted in 2015 [34].

Specifically on renewable energy, Mexico has adopted more policies than any other Latin American country. However, these policies do not aim to achieve specific renewable energy targets and mostly represent guidelines rather than policy requirements [32]. Only very recently has Mexico liberalised its heavily nationalised energy sector, which led to a faster uptake of renewable energy [35].

Figure S22. Instrument types and changes in emission intensity per sector in Mexico. *Carbon emission intensity is calculated as sectoral emissions from direct fossil fuel use only divided by whole economy GDP (blue & red dots, upper x-axis). The lower x-axis shows the number of adopted climate policies (climate policy density) by different types of policy instrument (grey-scale bars) in 2022.*

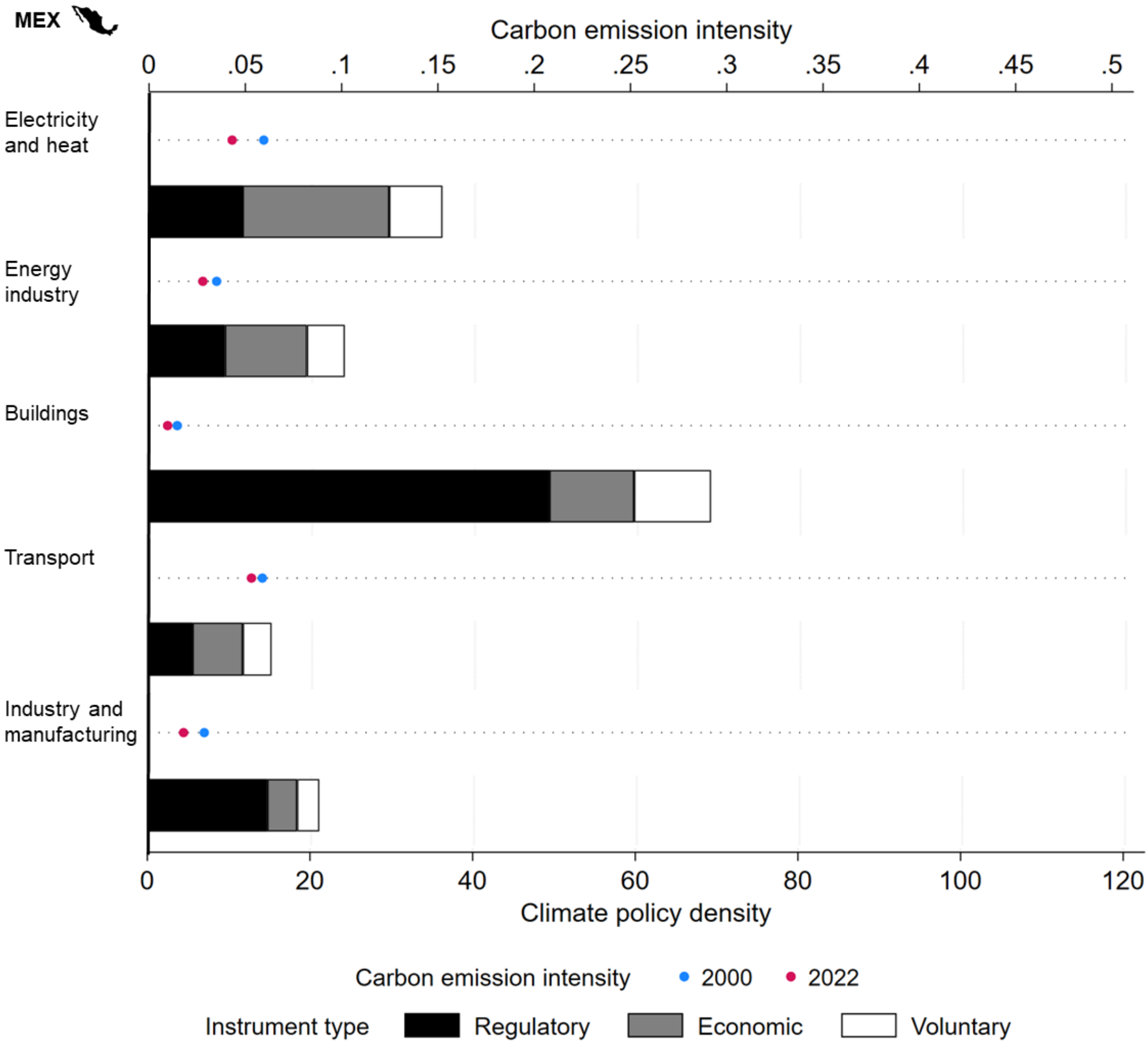

Note: Based on policy data from the IEA Policies and Measures Database (PMD) and carbon emission data from the IEA CO2 Emissions From Fuel Combustion Database.

In contrast to most other high-emitting countries, Mexico has only adopted relative emission reduction targets (i.e., emission reduction relative to certain baselines) and refrained from committing to specific absolute emission reduction targets. Originally, the General Law on Climate Change adopted in 2012 pledged to reduce greenhouse gas emissions by 30% with respect to a business as usual scenario by 2020. Updates to Mexico’s targets have further reduced its ambition, making policy less transparent and less specific in terms of individual sector contributions [36,37].

Figure S23. Historical development of Mexico's climate policy portfolio. *Carbon emission intensity is calculated as sectoral emissions from direct fossil fuel use only divided by whole economy GDP (red line, right-hand y-axis). Climate policy density is calculated as cumulative number of adopted climate policies (blue line, left-hand y-axis).*

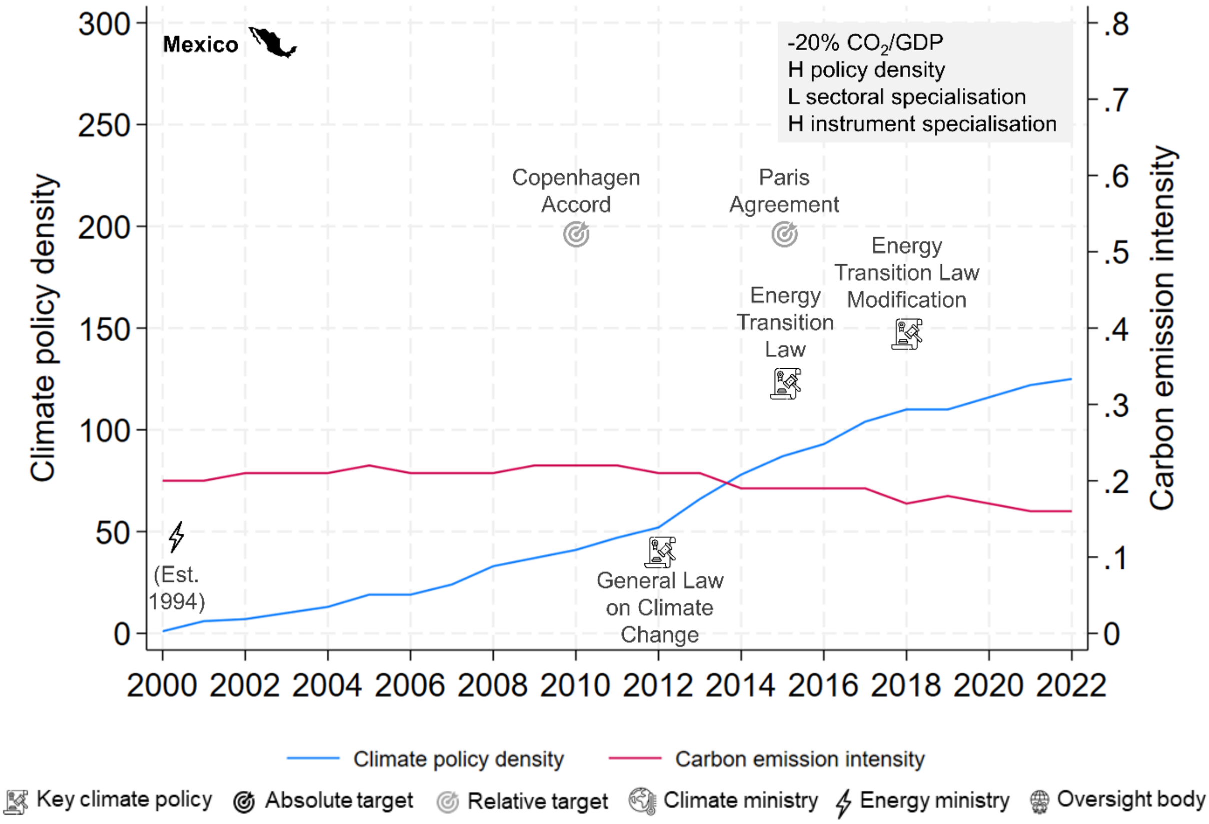

Note: Based on policy data from the IEA Policies and Measures Database (PMD) and carbon emission data from the IEA CO2 Emissions From Fuel Combustion Database. Data on targets, energy ministries and advisory bodies retrieved from various sources (see Table S1 in SI1: Data & Models).

Overall, the case of Mexico shows a mismatch between climate policy density and carbon emission reduction, which has been attributed to the failure to implement adopted climate policies due to shortcomings in the design of adopted policies, weak administrative capacity, and uneven political leadership [32,36].

6 Effect of energy ministries

Our econometric analysis indicates that the positive association between climate policy density and emission intensity reduction is stronger if a country has established a dedicated energy ministry.

6.1 United States of America

The USA established its Department of Energy already in 1977. There is some evidence that the ministry played a crucial role in the Obama administration's efforts to transform the energy sector and contributed to reducing electricity and heat production from coal and oil and improving energy efficiency [38]. It also played a key role in launching the Clean Energy

Ministerial as an intergovernmental platform to support the Obama administration's goal of promoting clean energy resources [39].

7 Effect of independent advisory bodies

Our econometric analysis indicates that the positive association between climate policy density and emission intensity reduction is stronger if a country has established an independent advisory body. The United Kingdom is an early example of this association.

7.1 United Kingdom

The United Kingdom (UK) is characterised by relatively Fast emission intensity reduction and a gradually increasing stock of climate policies. In 2008 the UK established an independent advisory body, the Committee on Climate Change (CCC), which has further advanced the UK's climate policy regime and its impact on emission reductions.

Figure S24. Historical development of the United Kingdom's climate policy portfolio. *Carbon emission intensity is calculated as sectoral emissions from direct fossil fuel use only divided by whole economy GDP (red line, right-hand y-axis). Climate policy density is calculated as cumulative number of adopted climate policies (blue line, left-hand y-axis).*

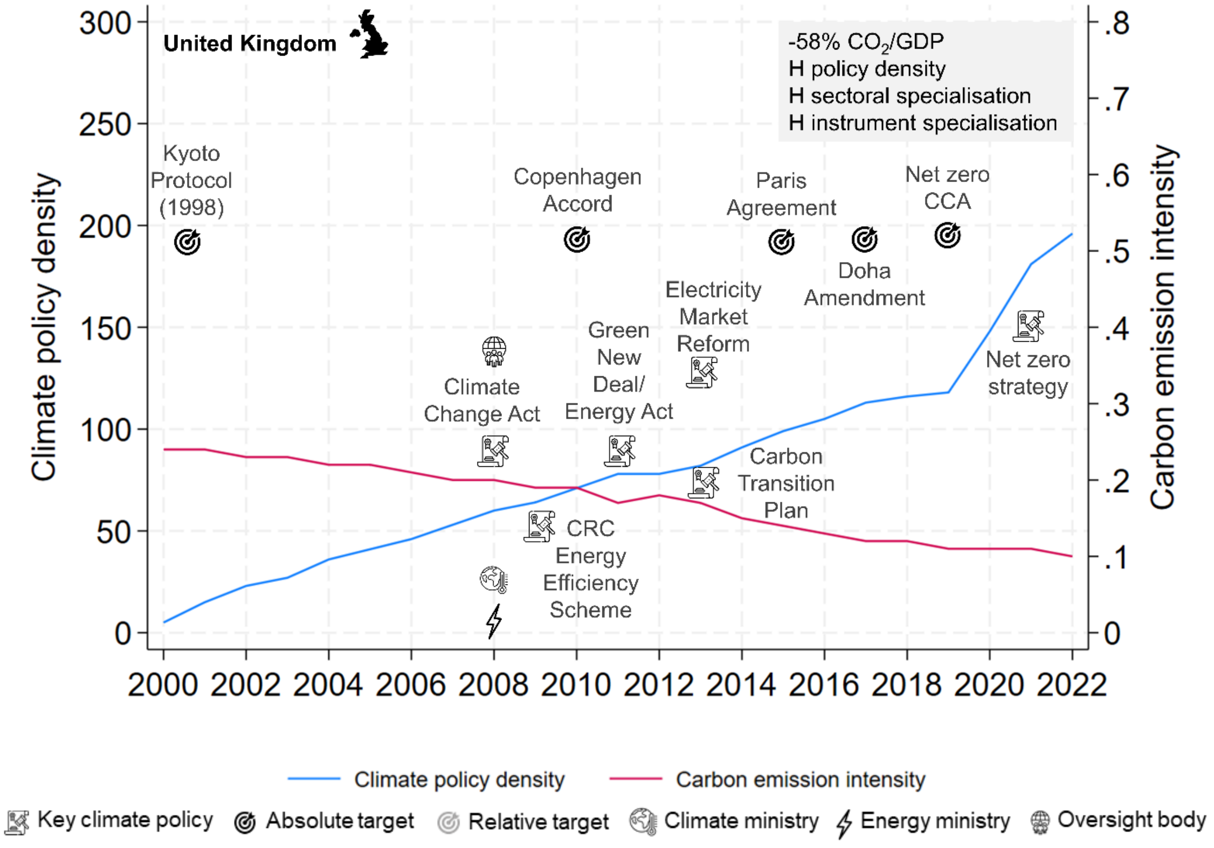

Note: Based on policy data from the IEA Policies and Measures Database (PMD) and carbon emission data from the IEA CO2 Emissions From Fuel Combustion Database. Data on targets, energy ministries and advisory bodies retrieved from various sources (see Table S1 in SI1: Data & Models).

The sharp decline in greenhouse gas emissions can be linked to one of the most stringent and ambitious climate policy regimes [40]. Its flagship is the Climate Change Act, which was adopted in 2008. This framework legislation included several innovative features, including legally-binding long-term mitigation targets, carbon budgets, an independent advisory body (the Committee on Climate Change), and regular and mandatory monitoring and reporting processes [40]. The Act had been designed to stimulate further policy action and hold decision-makers accountable, which has resulted in several key policy actions in the aftermath. This has included the Electricity Market Reform of 2013, which established a carbon pricing scheme that was mainly responsible for the rapid phase-out of coal. It further led to the Low Carbon Transition Plan, adopted in 2009. Energy efficiency in buildings had been addressed by further legislation, including the Green New Deal / Energy Act, adopted in 2011, and the CRC Energy Efficiency Scheme in 2013 [41]. The UK was part of the EU emissions trading system and then created its own scheme in 2021 after leaving the EU [42].

Figure S25. Instrument types and changes in emission intensity per sector in the United Kingdom. *Carbon emission intensity is calculated as sectoral emissions from direct fossil fuel use only divided by whole economy GDP (blue & red dots, upper x-axis). The lower x-axis shows the number of adopted climate policies (climate policy density) by different types of policy instrument (grey-scale bars) in 2022.*

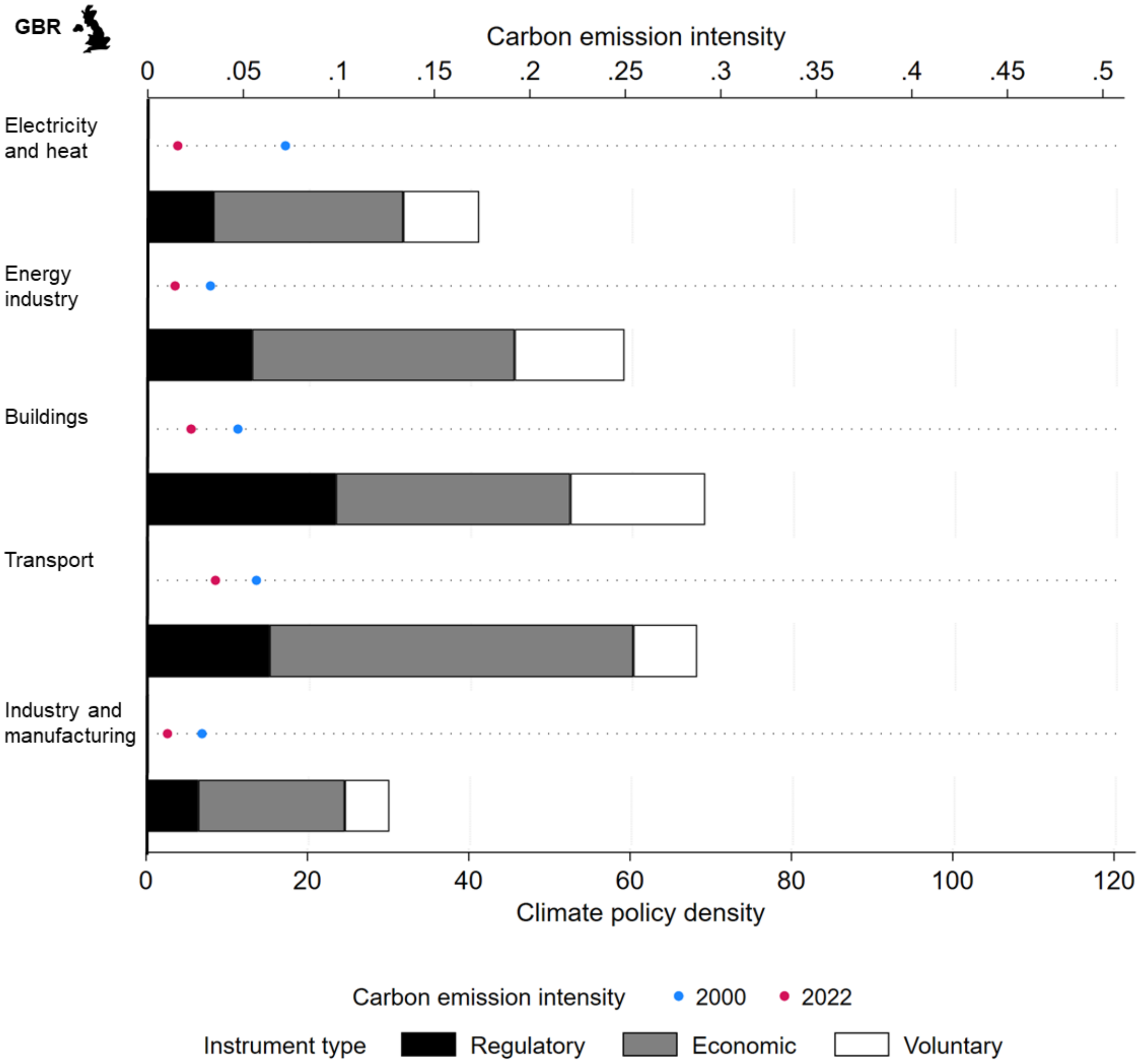

Note: Based on policy data from the IEA Policies and Measures Database (PMD) and carbon emission data from the IEA CO2 Emissions From Fuel Combustion Database.

The Committee on Climate Change (CCC) has supported the long-term emission reduction process [43]. It is staffed with technical experts independent of political parties, backed by a secretariat with expertise across relevant climate-related areas [43,44]. The CCC played a role in shaping the Electricity Market Reform. It also ensures the UK government is held to account for its progress in implementing climate policies [43].

The UK can be regarded as one of the most successful countries in reducing its greenhouse gas emissions. Most of the reductions have been achieved by the energy supply sector, due to a phase-out of coal and a significant expansion of nuclear and renewable energy [40]. Prior to 2016, energy had been the largest emitting sector. Due to the strong reductions, its absolute emissions have since become lower than those of the transport sector and those of the buildings sector.

Overall, the UK represents an illustrative example for a strong climate policy regime and strong reduction of emission intensity. Empirical research suggests that the Committee on Climate Change had a conducive effect on the government adopting and implementing ambitious climate policy.

8 Supplementary References

1. Jensen, L. & Grieger, G. United States climate change policies. State of play ahead of COP27. European Parliament, 2022.
2. Le Quéré, C. *et al.* Drivers of declining CO2 emissions in 18 developed economies. *Nat. Clim. Chang.* **9,** 213–217; 10.1038/s41558-019-0419-7 (2019).
3. Pischke, E. C., Solomon, B. D. & Wellstead, A. M. A historical analysis of US climate change policy in the Pan-American context. *J Environ Stud Sci* **8,** 225–232; 10.1007/s13412-018-0476-7 (2018).
4. Grant, D., Bergstrand, K. & Running, K. Effectiveness of US state policies in reducing CO2 emissions from power plants. *Nat. Clim. Chang.* **4,** 977–982; 10.1038/nclimate2385 (2014).
5. Smith, D. C. 'The' key 2024 election involving climate change and the energy transition: Biden vs Trump 2.0 and the matter of global trust in the US. *Journal of Energy & Natural Resources Law* **42,** 131–136; 10.1080/02646811.2024.2341542 (2024).
6. Erbach, G. & Jochheim, U. China's climate change policies. State of play ahead of COP27. European Parliamentary Research Service, 2022.
7. Sandalow, D. *et al. Guide to Chinese Climate Policy. 2022* (Oxford Institute of Energy Studies, 2022).
8. Climate Action Tracker. China. November 2022 Update, 2022.
9. Gallagher, K. S., Zhang, F., Orvis, R., Rissman, J. & Liu, Q. Assessing the Policy gaps for achieving China's climate targets in the Paris Agreement. *Nat Commun* **10,** 1256; 10.1038/s41467-019-09159-0 (2019).
10. Zhao, X., Yin, H. & Zhao, Y. Impact of environmental regulations on the efficiency and CO2 emissions of power plants in China. *Applied Energy* **149,** 238–247; 10.1016/j.apenergy.2015.03.112 (2015).
11. Viola, E. & Basso, L. Brazilian Energy-Climate Policy and Politics towards Low Carbon Development. *Global Society* **29,** 427–446; 10.1080/13600826.2015.1028904 (2015).

12. Kahlen, L. *et al.* Climate Governance in Brazil. Assessment of the government's ability and readiness to transform Brazil into a zero emissions society. NewClimate Institute; Climate Analytics, 2022.
13. Ashwarya, S. Israel's Renewable Energy Strategy. A Review of its Stated Goals, Current Status, and Future Prospects. *Perceptions* **26,** 321–340 (2021).
14. OECD. *OECD Environmental Performance Reviews. Israel 2023* (OECD Publishing, Paris, 2023).
15. Michaels, L. & Tal, A. Convergence and conflict with the 'National Interest': Why Israel abandoned its climate policy. *Energy Policy* **87,** 480–485; 10.1016/j.enpol.2015.09.040 (2015).
16. Li, R. & Jiang, R. Is carbon emission decline caused by economic decline? Empirical evidence from Russia. *Energy & Environment* **30,** 672–684; 10.1177/0958305X18802786 (2019).
17. Mitrova, T. & Melnikov, Y. Energy transition in Russia. *Energy Transit* **3,** 73–80; 10.1007/s41825-019-00016-8 (2019).
18. NewClimate Institute. Climate Action Tracker on Russia. November 2022 Update, 2022.
19. Farstad, F. M., Hermansen, E. A. T., Grasbekk, B. S., Brudevoll, K. & van Oort, B. Explaining radical policy change: Norwegian climate policy and the ban on cultivating peatlands. *Global Environmental Change* **74,** 102517; 10.1016/j.gloenvcha.2022.102517 (2022).
20. IEA. Energy Policy Review. Norway. International Energy Agency, 2022.
21. Ćetković, S. & Skjærseth, J. B. Creative and disruptive elements in Norway´s climate policy mix: the small-state perspective. *Environmental Politics* **28,** 1039–1060; 10.1080/09644016.2019.1625145 (2019).
22. Tatomir, S. Estonia's climate policy. challenges and opportunities. In *OECD Economic Surveys. Estonia 2022,* edited by OECD (OECD2022).
23. Andersen, M. S. The politics of carbon taxation: how varieties of policy style matter. *Environmental Politics* **28,** 1084–1104; 10.1080/09644016.2019.1625134 (2019).
24. Valdmaa, K. Development of the environmental taxes and charges system in Estonia: international convergence mechanisms and local factors. *Policy Studies* **35,** 339–356; 10.1080/01442872.2013.875152 (2014).
25. Kanger, L., Sovacool, B. K. & Noorkõiv, M. Six policy intervention points for sustainability transitions: A conceptual framework and a systematic literature review. *Research Policy* **49,** 104072; 10.1016/j.respol.2020.104072 (2020).
26. Ersoy, S. R., Terrapon-Pfaff, J. C., Abu Hamed, T. & Kádár, J. *Sustainable transformation of Israel's energy system. Development of phase model* (Friedrich-Ebert-Stiftung, Herzliya, 2021).
27. Jakob, M., Flachsland, C., Christoph Steckel, J. & Urpelainen, J. Actors, objectives, context: A framework of the political economy of energy and climate policy applied to India, Indonesia, and Vietnam. *Energy Research & Social Science* **70,** 101775; 10.1016/j.erss.2020.101775 (2020).
28. Nachmany, M. *et al.* The 2015 Global Climate Legislation Study. Climate Change Legislation in Indonesia. Grantham Institute, 2015.
29. NewClimate Institute. Climate Governance in Indonesia, 2021.

30. Resosudarmo, B. P., Ardiansyah, F. & Napitupulu, L. The Dynamics of Climate Change Governance in Indonesia. In *Climate Governance in the Developing World,* edited by D. Held, C. Roger & E.-M. Nag (Polity Press, Cambridge, 2013), pp. 72–90.

31. Abraham-Dukuma, M. C., Dioha, M. O., Okpaleke, F. N. & Bogado, N. Improving the climate change mitigation regime of major emitting countries: The case of South Africa, China, Germany and the United States of America. *Env Pol Gov* **32,** 43–55; 10.1002/eet.1961 (2022).

32. Pischke, E. C. *et al.* From Kyoto to Paris: Measuring renewable energy policy regimes in Argentina, Brazil, Canada, Mexico and the United States. *Energy Research & Social Science* **50,** 82–91; 10.1016/j.erss.2018.11.010 (2019).

33. Schreurs, M. A. The Paris Climate Agreement and the Three Largest Emitters: China, the United States, and the European Union. *PaG* **4,** 219–223; 10.17645/pag.v4i3.666 (2016).

34. Averchenkova, A. & Guzman Luna Sandra. Mexico's General Law on Climate Change. Key achievements and challenges ahead. Grantham Research Institute on Climate Change and the Environment and Centre for Climate Change Economics and Policy, London School of Economics and Political Science, 2018.

35. Natorski, M. & Solorio, I. Policy failures and energy transitions: the regulatory bricolage for the promotion of renewable energy in Mexico and Chile. *npj Clim. Action* **2**; 10.1038/s44168-023-00039-4 (2023).

36. Solorio, I. Leader on paper, laggard in practice: policy fragmentation and the multi-level paralysis in implementation of the Mexican Climate Act. *Climate Policy* **21,** 1175–1189; 10.1080/14693062.2021.1894084 (2021).

37. NewClimate Institute. Climate Action Tracker. Mexico. December 2022 Update. NewClimate Institute; Climate Analytics, 2022.

38. Nye, D. E. The United States and Alternative Energies since 1980: Technological Fix or Regime Change? *Theory, Culture & Society* **31,** 103–125; 10.1177/0263276414537314 (2014).

39. Tosun, J. & Rinscheid, A. The Clean Energy Ministerial: Motivation for and policy consequences of membership. *International Political Science Review* **42,** 114–129; 10.1177/0192512120942303 (2021).

40. Gransaull, G., Rhodes, E. & Fairbrother, M. Institutions for effective climate policymaking: Lessons from the case of the United Kingdom. *Energy Policy* **175,** 113484; 10.1016/j.enpol.2023.113484 (2023).

41. Rietig, K. & Laing, T. Policy Stability in Climate Governance: The case of the United Kingdom. *Env Pol Gov* **27,** 575–587; 10.1002/eet.1762 (2017).

42. Erbach, G. & Szczepanski, M. United Kingdom climate change policies. State of play ahead of COP27. European Parliament, 2022.

43. Averchenkova, A., Fankhauser, S. & Finnegan, J. J. The influence of climate change advisory bodies on political debates: evidence from the UK Committee on Climate Change. *Climate Policy* **21,** 1218–1233; 10.1080/14693062.2021.1878008 (2021).

44. Dudley, H., Jordan, A. & Lorenzoni, I. Advising national climate policy makers: A longitudinal analysis of the UK Climate Change Committee. *Global Environmental Change* **76,** 102589; 10.1016/j.gloenvcha.2022.102589 (2022).
